# From Nature to Synthesis and Vice Versa: Costic Acid Analogs with Acaricidal Activity Against the Bee Parasite *Varroa destructor*

**DOI:** 10.3390/plants15020310

**Published:** 2026-01-20

**Authors:** Eugenia Papastefanaki, Apostolos Spyros, Demosthenis Isaakidis, Maria Kallivretaki, Despoina Moraiti, Napoleon C. Stratigakis, Demetrios Ghanotakis, Haralambos E. Katerinopoulos

**Affiliations:** Department of Chemistry, University of Crete, Voutes Campus, 71003 Heraklion, Crete, Greece; chemp1133@edu.chemistry.uoc.gr (E.P.); aspyros@uoc.gr (A.S.); idimos@gmail.com (D.I.); kallivretakimaria99@gmail.com (M.K.); despoinamoraiti@hotmail.gr (D.M.); napoleonstr@uoc.gr (N.C.S.); ghanotakis@chemistry.uoc.gr (D.G.)

**Keywords:** *Inula helenium*, *Varroa destructor*, alantolactone, isoalantolactone, antioxidants, acaricides

## Abstract

The species *Inula helenium* belongs to the genus *Inula* (Asteraceae) and exhibits antibacterial and anti-inflammatory properties. It is used in respiratory and skin diseases. Its bioactivity is attributed to its eudesmanolide components, mainly to alantolactone and isoalantolactone. These components were isolated in high purity from the plant’s dried roots, either via multiple column chromatography separations or via repeated recrystallization. Two more eudesmanolides structurally similar to their parent compounds were isolated, namely 11,13-dihydro-alantolactone and 11,13-dihydro-isoalantolactone. The secondary metabolites and their derivatives were characterized in detail, for the first time, via NMR spectroscopy, GC-MS, and HRMS. Synthetic modification of the natural component structure was considered necessary for structure–activity relationship studies and biological tests. Thus, each compound was converted to its nitrile and then to the corresponding acid, or to its azide derivative and then corresponding amine. Antioxidant studies were conducted on the parent compounds, their derivatives, and the methanolic and hexane plant extracts using the DPPH radical method. The study revealed a strong antioxidant capacity of the methanolic extract. Acaricidal studies of both natural products and synthetic analogs against *Varroa destructor* and the comparison of their activity with the parent natural product costic acid, as well as one of its synthetic congeners, indicated that the “from nature to synthesis and vice versa” approach led to active compounds as well as to meaningful conclusions regarding the “pharmacophore” groups in the structural framework of the acaricides.

## 1. Introduction

### 1.1. The Genus Inula

The genus *Inula* belongs to the *Asteraceae* family, also known as Compositae. Over 590 different plants are reported to belong to the genus *Inula*, of which only 112 are confirmed and accepted (The Plant List Database). Of these, twenty (20) species are observed in China, nineteen (19) in the European area, and twenty-six (26) in areas of Turkey. It is native to Europe in both its Mediterranean and temperate zones, in temperate and tropical Asia (central-southern Asia), and tropical Africa (southeast Africa). Also, some species of the genus have acclimatized in North America and Australia, as well as in areas of Siberia. Therefore, an almost global distribution is observed for the genus *Inula*. It is biodistributed in most temperate and some tropical regions. However, it is unable to thrive in areas with extreme temperatures and dryness, such as the Earth’s poles and deserts [1,2,3].

### 1.2. Characteristics of Inula helenium

The species *Inula helenium* L. belongs to the Compositae family and is a perennial plant. It is native to central and western Asia. In the Bronze Age it spread to Europe and Britain. Presently, it has also acclimatized in various regions of America, Australia, and a few regions in Siberia and China [2]. In the English language the common name of the plant is Elecampane.

### 1.3. The Parasite Varroa destructor

Varroosis, or varroatosis, is one of the main diseases that affects the brood and the adult bee. It is caused by the *Varroa destructor* mite, which is an ectoparasite. Originally, varroa was attributed to the parasite *Varroa jacobsoni*, which parasitized the Asian honey bee *Apis cerana*. At least two species of the parasite were distinguished after studying the genotype, phenotype, and reproductive differences between populations of *V. jacobsoni*. In fact, the varroosis that affects the bee *Apis mellifera* in all other regions, including Greece, is due to the parasite *Varroa destructor* [4].

On an individual level, bees infected with varroa are affected both by the loss of hemolymph, which is important for the bee’s immune system, and by the wound caused by the parasite’s bite, which favors the establishment of bacterial infections and diseases. At higher levels of infection, the symptoms are severe. Among the main symptoms observed are loss of weight, deformation of the wings, and disorientation when returning to the hive [5].

### 1.4. Initial Studies—Background of the Project

**Figure 1:**
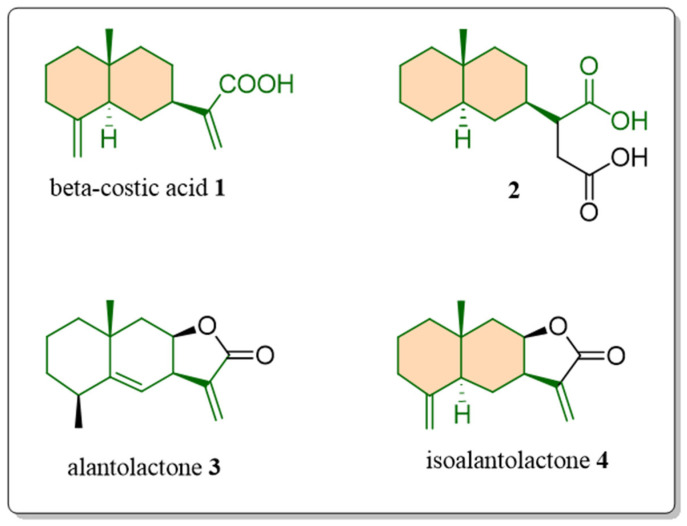
Structure of beta-costic acid (**1**), (R)-2-((2R,4aR,8aR)-4a-methyldecahydronaphthalen-2-yl)succinic acid (**2**), alantolactone (**3**), and isoalantolactone (**4**). Structural similarities are highlighted in green.

Prompted by the observation by local beekeepers that the use of branches of the plant for smoking beehives reduces the *Varroa destructor* population, we embarked on a project involving the phytochemical analysis of *I. viscosa* and the identification of the active ingredient(s). Our efforts were very successful, as the pesticide component in the plant was identified as costic acid (**1**) (Figure 1) and our results were secured by an international patent [6].

Our biological assays included oxalic acid, a natural product used by the beekeepers against varroosis. The fact that a complicated structure such as costic acid and the simplest of the bicarboxylic acids were almost equipotent convinced us that a synthetic compound incorporating both natural products in its carbon framework would be at least equipotent to the parent compounds. Indeed, amongst a number of synthetic congeners, (R)-2-((2R,4aR,8aR)-4a-methyldecahydronaphthalen-2-yl)succinic acid (**2**), with distinct resemblance to both natural products, was found to be much more potent than its “components” [7].

The mere fact that a synthetic compound is more active than a similarly structured natural product led us to the hypothesis that it was very likely that our product would resemble a compound that occurs in nature. Research of the literature led us to alantolactone (**3**) and isoalantolactone (**4**), mentioned above as components of *I. helenium,* a plant of the same genus as *I. viscosa.*

The work described in this publication had a dual purpose: first, the isolation of the two lactones from the plant; and second, the synthesis of structures with the highest possible resemblance to our synthetic compound. The novelty of this study lies in its approach for identifying pharmaceutical substances, namely, it does not only include the use of natural products (in our case costic acid) as lead compounds for the synthesis of active analogs, but also the use of the synthetic products as lead compounds for the isolation of active natural products with similar structures. This procedure leads to the isolation of active natural compounds which are more abundant than costic acid.

In our work by Georgiladaki et al. [7] involving the enantioselective synthesis of costic acid analogs, the synthetic compound exhibited stronger activity, reaching 100% *Varroa destructor* mortality in two hours, whereas costic acid had a similar activity at least eight hours after its application. Alantolactone and isoalantolactone exhibit lower but similar activity (75% mortality in 18 h), but have the advantage of being more abundant than costic acid.

## 2. Results and Discussion

### 2.1. Structure Elucidation of Isolated Components from Inula helenium

Alantolactone

**Figure 2:**
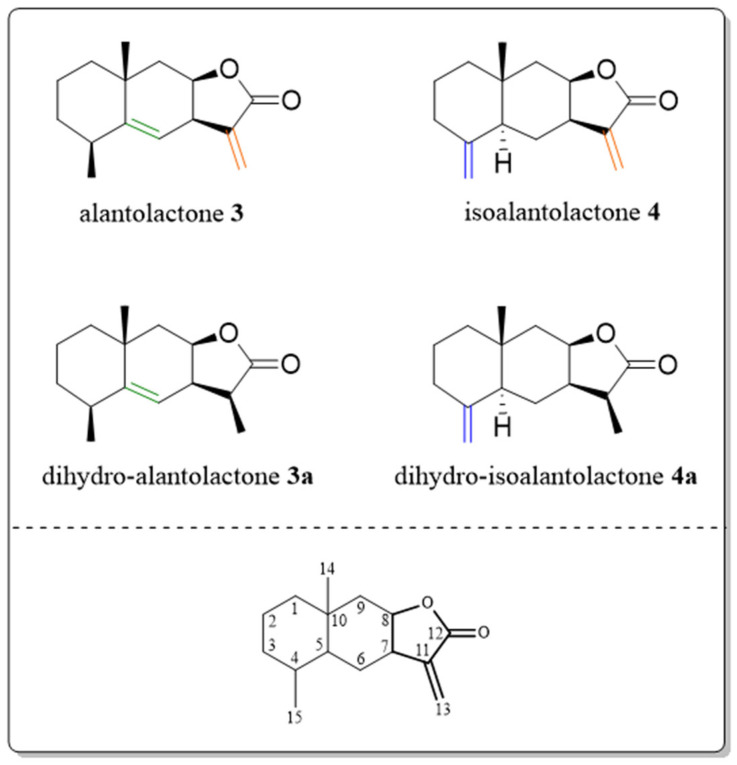
Isolated compounds from *Inula helenium* and their general numbered structure. Double bonds at the same position in different isomers are highlighted with the same colors.

The first secondary metabolite isolated was 8-hydroxy eudesma-5,11(13)-dien-12-ic acid-γ-lactone, also known as alantolactone (**3**) (Figure 2), which is a sesquiterpene lactone of the eudesmanolide group. The structure of alantolactone is bicyclic, consisting of an α-methylene-γ-lactone, exocyclic methyl groups at positions 10 and 4, an intramolecular double bond between positions 5 and 6, and a methylene group on position 11. In Table 1**,** one- and two-dimensional NMR data of compound **3** are presented.

Isoalantolactone

The second secondary metabolite isolated was 8β-hydroxy-linked 4(15),11(13)-dien-12-ic acid-γ-lactone, also known as isoalantolactone (**4**). Isoalantolactone is also a sesquiterpene lactone belonging to the group of eudesmanolides. The structure of isoalantolactone consists of a tricyclic sesquiterpene which has been metabolized to form an exocyclic methylene-group in the 4-position and contains an α-methylene-γ-lactone. In Table 2, one- and two-dimensional NMR data of compound **4** are presented.

11,13-dihydro-alantolactone

The third secondary metabolite isolated was 8β-hydroxy 4α-H-eudesma-5-en-12-ic acid-γ-lactone, also known as 11,13-dihydro alantolactone (**3a**), which is a sesquiterpene lactone of the group of eudesmanolides. The structure of dihydro-alantolactone includes a bicyclic sesquiterpene, containing an α-methyl-γ-lactone, an exocyclic methyl group at position 10 and position 4, an intramolecular double bond between positions 5 and 6, and a methyl group at position 11. A detailed NMR study of compound **3a** is presented in Table 3.

11,13-dihydro-isoalantolactone

The fourth secondary metabolite isolated was the compound 8β hydroxy-eudesma-4(15)-en-12-ic acid γ-lactone, also known as 11,13-dihydro isoalantolactone (**4a**), which is a sesquiterpene lactone of the group of eudesmanolides. The structure of 11,13-dihydroisoalantolactone also consists of a tricyclic sesquiterpene, which contains an α-methyl-γ-lactone, an exocyclic methyl group at position 10, an exocyclic methylene group at position 4, and a methyl group in position 11. A detailed NMR study of compound **4a** is presented in Table 4. 

The assignment of each proton/carbon was based on the HMBC, HSQC, and COSY experiments. The distinct carbonyl carbon in each compound was the starting point for the assignment. From that point, protons at positions 11 and 13 can be validated, proceeding with the rest. Furthermore, the singlet and doublet peaks at positions 14 and 15, respectively, also serve as key points for assigning neighboring protons and carbons. In the case of the **3a** derivative, the main difference from **3** is the methyl group at position 13. These methyl protons were mainly assigned using HMBC, given that these methyl protons interact with the carbonyl carbon at position 12. The same procedure is applied for the **4a** derivative.

Regarding previous assignments, our ^13^C-NMR data from compound **3** are identical to those published by Kumar et al. [8] and similar to those published by Klochkov et al. [9], since we assign the peak at 32.7 ppm to position 2 instead of position 3, as they propose, and the peak at 16.73 ppm to position 3 instead of position 2. Similarly, we assign the carbon at 149.1 ppm to the carbon of position 5 and the peak at 139.83 ppm to the carbon of position 11, while they propose the opposite. However, our data for protons of the same compound are identical to those published by Klochkov et al. [9] and Gökbulut et al. [10] with minor differences, mainly of the protons of positions 1, 2, and 3 for compound **3**, where the authors report a range in ppm or they do not provide any for H-5, whereas we were able to make distinct assignment.

For compound **4**, our 13C-NMR data are identical to those published by Kumar et al. [8] and Klochkov et al. [9], while our proton data are largely identical to those published by Klochkov et al. and Gökbulut et al. [10], with minor differences mainly of the protons of positions 1, 2, and 3, where the authors report a range in ppm or they do not provide any for H-5, whereas we were able to make distinct assignment.

It must be noted that any differences in our NMR data compared with those in the literature are expected, given that their NMR instrument resolution is lower than ours.

### 2.2. Synthesis of Alantolactone and Isoalantolactone Derivatives

#### 2.2.1. Alantolactone Derivatives

**Scheme 1:**
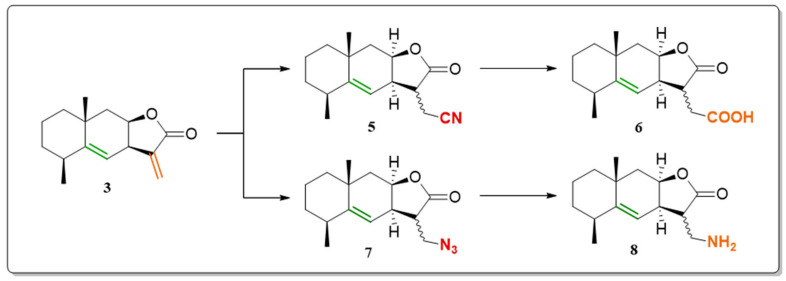
Synthetic pathway of alantolactone derivatives. Double bonds at the same position in all compounds are highlighted in green. Changes in functional groups affected by the reactions are highlighted in different colors.

*Nitrile introduction to alantolactone*: The reaction takes place via a 1,4-Michael-type addition, with the nitrile ion as the donor. This reaction was tested by Nagata’s group in various Michael systems [11] and was applied in the present work under modified conditions. The reaction was complete within three hours with 98% conversion, giving the two diastereomers (**5**) shown above as products (Scheme 1). However, the separation of the two diastereomers was not possible.

The stereochemistry of each diastereomer was assigned based on two main signals. In alantolactone it is known, both from the literature and from the NOESY spectrum, that H-7 and H-8 are pseudo-equatorial to each other, i.e., both are located in the same direction, “behind” the plane defined by the molecule. In the case of **5*R***, H-7 (δ_H_ 3.29 ppm) shows an interaction with H-8 (δ_H_ 4.83 ppm) in the ^1^H-^1^H NOESY spectrum, but it also interacts with H-11 (δ_H_ 3.18 ppm). Respectively, H-11 shows an interaction with H-7, H13a (δ_H_ 2.88 ppm), and H13b (δ_H_ 2.46 ppm). Therefore, it is concluded that H-11 is also in the same direction as H-7 and H-8, “behind” the plane of the molecule, i.e., the stereogenic center has an *R* configuration. On the other hand, in the case of **5*S***, in the ^1^H-^1^H NOESY spectrum, the interaction of H-7 (δ_H_ 3.01 ppm) with H-8 (δ_H_ 4.96 ppm) is clearly visible, but not with H-11 (δ_H_ 2.66 ppm). Moreover, H-11 only interacts with H-13a (δ_H_ 2.82 ppm). In this case, H-11 is oriented in front of the plane of the molecule and the configuration of the stereogenic center is *S*.

*Hydrolysis of alantolactone nitrile to acid:* Hydrolysis of the nitrile is the next step, which takes place under basic conditions. This reaction was reported by Hansen in 1933 [12]; however, the present work required milder heating. Under these basic conditions of the reaction, the nucleophilic attack of the base on the lactone ring is observed, resulting in its opening. Closing of the ring requires heating to 80 °C under acidic conditions for 40 min. The reaction gives the two diastereomeric products **6*R*** and **6*S***, but by-product formation is also observed at five percent (5%). It is expected that the abstraction of H-11 in ***5R*** and ***5S*** will take place at different rates since the compounds are diastereomers and a difference in activation energy of the reaction is expected. Indeed, the nitrile difference in the diastereomeric ratio was 65:35 and after their hydrolysis to acids it changed to 55:45. This happens because H-11 is in the α-position to the carbonyl and simultaneously in the β-position to the nitrile and is, therefore, acidic enough to be removed under the strong basic conditions of the reaction. Abstraction and subsequent attack of the proton from either face of the molecule causes a change in the diastereomeric ratio.

Regarding the confirmation of the stereochemistry of each diastereomer, for compound **6*R*** there is a weak interaction of H-11 (δ_H_ 2.73 ppm) with H-7 (δ_H_ 2.84 ppm) and H-8 (δ_H_ 4.89 ppm). Therefore, H-11 is oriented towards the “back” side of the molecule and the resulting stereogenic center has an *R* configuration. A similar comment cannot be made for the secondary isomer of acid **6*S***, since the signals of H-11 (δ_H_ 3.28 ppm) and H-7 (δ_H_ 3.27 ppm) of the two diastereomers coincide.

*Azide addition into alantolactone:* The Michael addition of azide was performed according to Zaki’s protocol [13]. The Michael reagent chosen was trimethylsilyl azide (TMSN_3_), while the reaction took place in anhydrous conditions under a nitrogen atmosphere. At the end of the reaction, two diastereoisomeric products are formed, **7*R*** and **7S**.

To verify the stereochemistry of the products, a ^1^H-^1^H NOESY spectrum was recorded. In the case of **7*R***, the interaction of H-11 (δ_H_ 3.06 ppm) with H-7 (δ_H_ 3.19 ppm), H-13a (δ_H_ 3.80 ppm), H-13b (δ_H_ 3.43 ppm), and weakly with H-8 (δ_H_ 4.78 ppm) was noted. In the case of **7*S***, H-11 (δ_H_ 2.53 ppm) interacts only with H-13a (δ_H_ 3.70 ppm) and H-13b (δ_H_ 3.70 ppm). Therefore, in **7*R***, H-11 and H-7 are located in the same direction and the configuration of the stereogenic center is *R*, while in **7*S*** they are opposite to each other and the stereogenic center configuration is *S*.

*Reduction of alantolactone azide to an amine:* A final transformation carried out for the specific substrate is the reduction of the azide to the corresponding amine. This reaction is carried out through the interaction of the substrate with triphenyl phosphine (PPh_3_), a classic Staudinger reaction, yielding the corresponding iminophosphine of the compound. Upon acid hydrolysis, the iminophosphine yields the desired amine.

However, only one of the two diastereomers is isolated; the theoretically expected mixture of **8*R*** and **8*S*** was not detected due to the large diastereomeric excess of nitrile **7** of *R* configuration at the C-11 stereocenter. The attempt to confirm its stereochemistry by ^1^H-^1^H NOESY spectrum was not conclusive, given the fact that the H-7, H-11, and H-13 resonance signals are overlapping. Nevertheless, the apparently significant difference in diastereomeric excess between nitriles **7*R*** and **7S** leads to the conclusion that the product is of *R* configuration.

#### 2.2.2. Isoalantolactone Derivatives

**Scheme 2:**
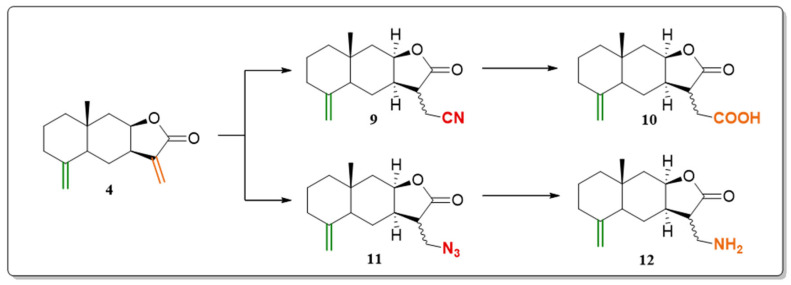
Synthetic pathway of isoalantolactone derivatives. Double bonds at the same position in all compounds are highlighted in green. Changes in functional groups affected by the reactions are highlighted in different colors.

*Nitrile insertion into isoalantolactone*: As in the case of alantolactone, the reaction is a Michael addition with the nitrile ion as the donor. The reaction was complete within three hours with 95% conversion, giving the two diastereomers, shown above, as products (Scheme 2). The diastereomeric excess was determined by both the quantitative analysis of the GC-MS data and the ^1^H NMR spectrum, since all peaks appeared separately. Of the product, **9*R*** formed 90%, while **9*S*** formed 10%. However, separation of the two diastereomers was not possible.

Regarding the stereochemistry of each diastereomer, the ^1^H-^1^H NOESY study was focused on the major product **9*R***, where H-7 (δ_H_ 2.68 ppm) clearly interacts with H-11 (δ_H_ 3.12 ppm), and vice versa. Also, H-8 (δ_H_ 4.57 ppm) interacts with H-11 and H-11 interacts with H-7, H-8, H-13a (δ_H_ 2.90 ppm), and H-13b (δ_H_ 2.53 ppm). Therefore, it is concluded that, in this structure, H-11 is in the same direction, with H-7 and H-8 “behind” the plane of the molecule, so the configuration of the stereogenic center is *R*.

*Hydrolysis of isoalantolactone nitrile to acid:* The next step is the hydrolysis of the nitrile of isoalantolactone to form an acid, which takes place under basic conditions. In the case of isoalantolactone, despite strongly basic conditions, no lactone ring opening was observed.

As expected, the reaction gives the two diastereomeric products **10*R*** and **10*S***, but ten percent (10%) by-product formation is also observed. The diastereomeric excess is determined from the ^1^H NMR spectrum, since the signals appear separately, with **10*R*** forming 68% and **10*S*** forming 32% of the product. Both the diastereomeric ratio and the existence of a by-product were also confirmed by quantitative analysis of the corresponding GC-MS data, after treatment of the acids with N,O-Bis(trimethylsilyl)trifluoroacetamide (BSTFA).

It is important to underline once more the difference in the ratio of the diastereomers of the nitrile starting materials (90:10) and the acids (68:32). This is also due to the acidic nature of H-11 as it is in an α-position to the carbonyl and at the same time in a β-position to the nitrile. Elimination, and subsequent attack of the proton from either face of the molecule, results in a change in the diastereomeric ratio.

The stereochemistry of the reaction products is established through the ^1^H-^1^H NOESY spectrum. In compound **10*R***, the interaction between H-11 (δ_H_ 2.71 ppm) and H-7 (δ_H_ 2.32 ppm) is evident. In the case of product **10*S***, no similar interaction of H-11 (δ_H_ 3.21 ppm) with H-7 (δ_H_ 2.62 ppm) is observed. H-11 interacts only with H-13a (δ_H_ 2.94 ppm) and H-13b (δ_H_ 2.60 ppm) and weakly with H-6a (δ_H_ 1.13 ppm). Therefore, in product **10*R,*** the stereogenic center of C-11 has an *R* configuration, while **10*S*** has an *S* chiral center.

*Azide insertion into isoalantolactone:* Isoalantolactone undergoes a 1,4-addition of azide, under the same conditions as alantolactone and using trimethylsilyl azide (TMSN_3_) under dry conditions and a nitrogen atmosphere. During the reaction, the formation of only one diastereomer **11*R*** is observed in the NMR spectra.

The stereochemistry of product **11*R*** is verified by the ^1^H-^1^H NOESY spectrum which shows a strong interaction of H-11 (δ_H_ 2.98 ppm) with H-7 (δ_H_ 2.57 ppm) and H-8 (δ_H_ 4.51 ppm). Therefore, H-11 is oriented toward the beta face of the molecule and the configuration of the C-11 stereocenter (δ_C_ 47.2 ppm) is *R.*

*Reduction of isoalantolactone azide to amine*: The next transformation of the substrate is a reduction of the azide to the corresponding amine. This reaction, as in the case of alantolactone, takes place through the interaction of the substrate with triphenylphosphine (PPh_3_), a classic Staudinger reaction, forming the corresponding iminophosphine that, upon hydrolysis, yields the desired amine **12*R***.

As expected from the stereochemistry of the reactant, but also confirmed by the ^1^H-^1^H NOESY spectra, in product **12*R,*** the configuration of the stereogenic center of C-11 (δ_C_ 49.6 ppm) is *R*, since H-11 is in the beta face of the molecule and therefore it exhibits a strong interaction with H-7 (δ_H_ 2.67 ppm) and H-8 (δ_H_ 4.56 ppm).

**Figure 3:**
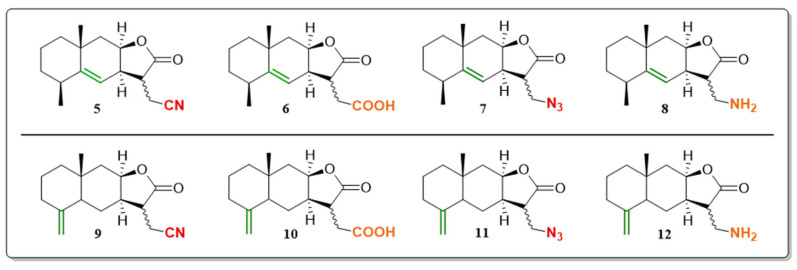
List of synthetic derivatives of alantolactone and isoalantolactone. Double bonds at the same position in all compounds are highlighted in green. Changes in functional groups affected by the reactions are highlighted in different colors.

A list of synthetic derivatives of alantolactone and isoalantolactone is shown in Figure 3.

A summary of the percentage yields and diastereomeric ratios of all synthetic products is presented in Table 5.

### 2.3. Biological Assays

#### 2.3.1. Antioxidant Study

The study of antioxidant capacity via the DPPH method was performed for the isolated compounds alantolactone (**3**) and isoalantolactone (**4**)**,** as well as for all of their synthetic derivatives. The antioxidant evaluation was conducted independently of the acaricidal assays and was intended to provide a broader characterization of the redox-related properties of the isolated compounds and plant extracts, rather than to imply a mechanistic relationship between radical-scavenging capacity and acaricidal activity.

A range of concentrations of the order of 0.01 to 50 mM was tested for all compounds, since at this range antioxidant activity is observed for the compounds of the terpene class [14,15]. Alantolactone (**3**) did not show strong reducing activity towards DPPH, with the maximum inhibition rate of the DPPH· radical reaching 8.20% at a concentration of 12.5 mg/mL (53.88 μM). However, the amine of alantolactone (**8**) showed significant antioxidant activity, since complete inhibition of the radical was observed at a concentration of 12.5 mg/mL. The IC_50_ value was 4.42 ± 0.38 mg/mL, which, when expressed in molarity, corresponds to 17.74 ± 1.52 mM.

Except for the amine of alantolactone (**8**), the rest of the synthetic derivatives of alantolactone did not show strong antioxidant activity, with the observed inhibition of the DPPH radical ranging from 10.68% and below, as shown in the diagram (in Supplementary Information). Again, isoalantolactone (**4**)**,** did not exhibit strong reducing activity towards the DPPH radical, since the maximal scavenging activity was observed at a concentration of 12.5 mg/mL (53.84 mM, 4.60%). As expected, none of the synthetic derivatives of isoalantolactone (**4**) showed significant activity, with the percentage of radical inhibition by the compounds ranging from 5.16% for the isoalantolactone amine (**12**) to lower. Due to the low antioxidant activity of the compounds, it was not possible to calculate the IC_50_ values.

Subsequently, we focused on the methanolic and hexane total extracts of *I. helenium*, which were studied in a concentration range from 0.01 mg/mL to 25 mg/mL. The hexane extract showed a reducing activity towards DPPH, with radical inhibition at a concentration of 25 mg/mL reaching 98.34% and the IC_50_ being calculated as 14.00 ± 0.5 mg/mL. Therefore, it can be safely concluded that the antioxidant activity displayed by the hexane extract is not largely due to alantolactone (**3**) and isoalantolactone (**6**)**,** but to other components of the extract.

However, the methanolic extract exhibited a strong antioxidant capacity with radical inhibition at a concentration of 25 mg/mL, reaching 99.02% with a calculated IC_50_ of 0.60 ± 0.05 mg/mL.

The phytochemical profiling of extracts of *Inula helenium* has been reported [16], aiming for investigation of the chemical composition of *Inula helenium* extracts and the evaluation of the antioxidant potential conferred by the chemical constituents.

Antioxidant constituents found in the roots, leaves, and flowers of *Inula helenium* include phenolic acids (caffeic acid, chlorogenic acid, hydroxybenzoic acid, and ferulic acid derivatives) flavonoids (quercetin, kaempferol, luteolin, epicatechin, and catechingallate), and terpenoids (β-caryophyllene). Sesquiterpene lactones such as alantolactone and isoalantolactone have been mentioned to be antioxidants and anti-inflammatory agents [17].

It should be noted that all studies were carried out using ascorbic acid as a standard, for which the corresponding DPPH radical inhibition percentage plot was constructed. Its IC_50_ was calculated as equal to 0.0187 ± 0.0003 mg/mL, or expressed in molarity equal to 0.1061 ± 0.0017 mM. A summary table follows, with the calculated IC_50_ values of the samples showing antioxidant activity, as well as the R^2^ coefficients for the data fitting model used (Table 6).

Sesquiterpene lactones bearing an α-methylene-γ-lactone motif have been widely studied, and structure–activity relationships suggest that this electrophilic functionality can interact with biological nucleophiles, contributing to diverse biological effects [18,19]. However, no correlation was observed between DPPH radical-scavenging activity and acaricidal potency in the present study, suggesting that redox-mediated mechanisms are not central to the acaricidal action against *Varroa destructor*. The enhanced DPPH radical-scavenging activity observed for the alantolactone amine derivative is most likely attributed to the introduction of a proton-donating amine functionality and does not reflect a general feature of the eudesmanolide scaffold.

#### 2.3.2. Acaridical Study

Analysis of the data of the acaricidal studies led to a number of interesting results. Activity of the natural products isoalantolactone (**4**) and alantolactone (**3**) is expected, given their structural resemblance to beta-(**1**) and alpha-costic acid, respectively (see Supplementary Information).

Their relatively lower activity compared to that of their parent compounds could be attributed to the fact that their carboxylic acid moieties are “masked” in the form of lactonic rings between the C-7 and C-8 positions.

**Figure 4:**
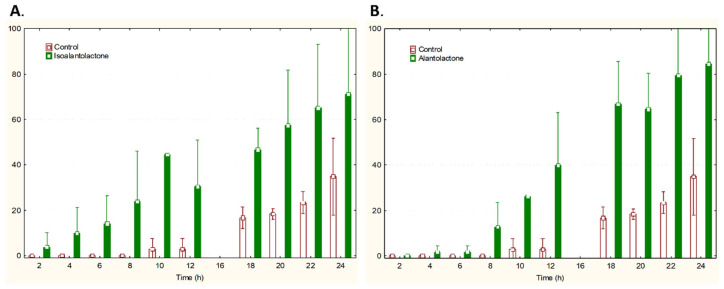
Percentage of acaridical mortality of *Varroa destructor* when treated with (**A**) isoalantolactone and (**B**) alantolactone, over time.

Another interesting point that emerges from the comparison of the potency results refers to the rate of increase in the potency of the analogs. Costic acid (**1**) exhibits 100% activity in fourteen hours; however, it reaches 50% activity at seven hours of exposure of the mites to the drug. Isoalantolactone (**4**) and alantolactone (**3**) reach 80% activity in 24 h, but in half the time (12 h) they also exhibit half of their maximal activity (Figure 4). It appears, therefore, that **4** and **3** are both less active and slower than the parent compound (**1**), but they exhibit similar rate of potency.

**Figure 5:**
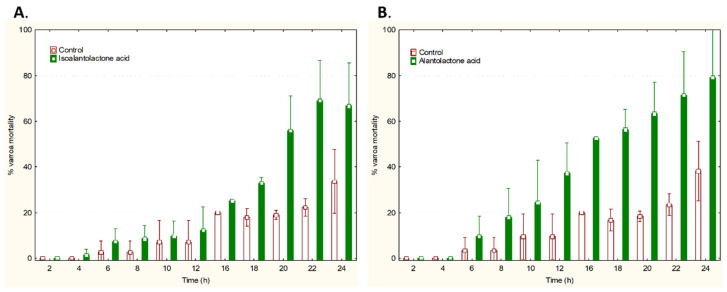
Percentage of acaridical mortality of *Varroa destructor* when treated with (**A**) isoalantolactone acid and (**B**) alantolactone acid, over time.

A similar interpretation could be offered in the case of the corresponding isoalantolactone and alantolactone acids (**10** and **6**), given that the isoalantolactone acid (**10**) is structurally similar to the synthetic product **2** reported previously by our group [7] (Figure 5). Again, the lower activity of **10,** compared to that of **2,** indicates that the “pharmacophore” of the acaricidal drugs preferably contains a free carboxylic moiety rather than its lactone analog.

The acaricidal profile of bis-carboxylic acid **2** is remarkable: *Varroa destructor* mortality is over 80% in two hours and reaches 100% in six hours. The fact that the isoalantolactone and alantolactone acid congeners (**10** and **6**) reach a maximal acaricidal activity of 80% after 24 h indicates that the active site binding the costic acid-type drugs contains a carboxylate-binding domain that favors binding free carboxylate moieties rather than their lactonic analogs.

Regarding the alantolactone amine **8**, its inertness is apparent when the control values are subtracted for the corresponding activity values of the amine. The mean activity values are maximal at 20% (see Supporting Information).

Phytochemical analysis of *Inula helenium* extracts did not reveal the presence of amines, in contrast to the numerus acids isolated in the study. This result may indicate that the “pharmacophore” interacts with the active site through a carboxylic moiety rather than an aliphatic amine.

Carboxylic acids are known to interact with the amino groups of proteins in binding sides. The presence of the carboxylate moiety might be a sufficient but not necessary condition for acaricidal activity and could hold only for eudesmane-type compounds. The fact that formic acid and oxalic acid are as active as costic acid implies that the carboxylic moiety, even in its simplest form, activates the acaricidal process. However, other acaricides that act against *Varroa destructor* do not contain carboxylic groups: cumaphos is an organophosphate, amitraz is a formamidine, and tau fluvalinate and flumethrine are pyrethroid esters. These compounds are active [17], apparently through different interactions with the active site.

In a previous publication by our group [6], insecticide Byvarol (active ingredient flumethine) was found equipotent to costic acid, the lead compound in our studies. Pyrethroid insecticides like flumethrinare control the *Varroa destructor* mite through interactions with the sodium channel [20]. The active site interaction is through L925 (Leucine 925).

Organophosphorus insecticides, such as cumaphos, act via phosphorylation of the serine hydroxyl group within the active site of AChE [21].

The acaricide amitraz is known to act on octopamine receptors; however, the exact molecular target site in Acari remains unclear. However, there is evidence showing that βAOR is the main target of DPMF(N2-(2,4-Dimethylphenyl)-N1-methyformamidine), the active metabolite of amitraz [22]. There is evidence that I61F SNP is associated with resistance to amitraz [23]. I61F SNP is a specific genetic mutation where an Isoleucine amino acid is replaced by a Phenylalanine at position 61 in the RmβAOR gene.

From the above data, we may conclude that each class of insecticides has a different mode of interaction with the active sites.

## 3. Materials and Methods

### 3.1. General Experimental Procedures

All purchased chemical reagents (Sigma-Aldrich, Merck and Fluka, Taufkirchen, Germany) were of the highest commercial purity and were used without prior purification. The 1D NMR spectra ^1^H and ^13^C and the 2D NMR spectra COSY, ^1^H-^1^H NOESY, HSQC, and HMBC were recorded in solutions of CDCl_3_ or CD_3_OD and are referenced to the residual peaks at δ = 7.26 ppm and δ = 77.0 ppm for CDCl_3_ and at δ = 3.31 ppm and δ = 49.05 ppm for CD_3_OD for ^1^H and ^13^C, respectively. All analyses were performed on a BrukerFT-NMRAMX 500(Bruker, Rheinstetten, Germany), operating at 500 MHz for ^1^H and 125 MHz for ^13^C. The chemical shifts are expressed in δ ppm and the coupling constants (*J*) in Hz. GC–MS was carried out using a ShimadzuNexisGC-2030 instrument (Tokyo, Japan), which was equipped with a MEGA-5 HT capillary column (30 m × 0.25 mm diameter × 0.25 μm thickness) and was coupled to a Shimadzu GCMS-QP2020NX 24 mass detector, Tokyo, Japan. Silica gel 60 F_254_ plates: Merck KGaA, Darmstadt, Germany were used for TLC. Spots on TLC plates were detected first under a UV lamp on 254 nm and 365 nm and then by heating after spraying with 7% ethanolic solution of phosphomolybdic acid.

### 3.2. Plant Material Collection

The roots of *Inula helenium* used in this study have been purchased from the company Dictamus, Genuine Cretan Herbs, Heraklion, Crete, Greece, while the sample was harvested from the region of Serbia during the month of October 2019. The received material was chopped into small pieces which were dried in the dark.

### 3.3. Extraction Procedure

First Extraction Method

The roots of the plant were crushed into smaller pieces (0.5–2 mm) and placed in a conical flask containing 600 mL of methanol [8]. After stirring for two days at room temperature, the extract was collected by filtration with a Buchner funnel and subsequent removal of the solvent in a rotary evaporator. The roots were again subjected to methanol (600 mL) extraction for an additional two-day period as above. The combined extract residues were dissolved in 600 mL of hexane and stirred for two days at room temperature with a magnetic stirrer. The hexane extract was isolated by filtration and removal of the solvent.

Second Extraction Method

A total of 250.02 g of *Inula helenium* roots (250.0 g) were crushed and divided into two 125 g parts. Each portion was placed in an Erlenmeyer flask containing 500 mL of chloroform and stirred for two days at room temperature. The extracts were collected and the roots of the plant were subjected to the same procedure again. The two extracts were combined, filtered under vacuum in a Buchner funnel, and the solvent was concentrated in vacuo using a rotary evaporator. The brownish extract was of a sticky and thick texture. Finally, the residue was filtered on a Schott filter topped with 1 cm silica gel layer using benzene as eluent, until the eluate was colorless, obtaining the “benzene extract”. The filter was again eluted with methanol, yielding the “methanolic extract”.

During the optimization of the extraction and isolation methods, two different extraction methods were tested. Their choice was based on common methods of extraction for the plants. However, as shown by NMR spectra, both the final hexane extract of the first method and the final benzene extract of second method resulted in similar extract profiles. The preference of the first method was based on the fact that hexane is a less toxic solvent compared to benzene and chloroform. Regardless of the extraction method, the desired compounds were isolated following the same procedure (number of columns and solvent system).

### 3.4. Antioxidant Study with DPPH

Method: The antioxidant capacity of the compounds and plant extracts was determined through the DPPH method, following a modified protocol based on the method of Brand–Williams et al. [24]. According to the method, DPPH (2,2-diphenyl-1-picryl-hydrazyl), a stable radical in methanolic solution, has a maximum absorption at 515 nm, imparting a purple color to the solution. In the presence of substances with antioxidant activity, the DPPH radical is reduced to DPPH-H (2,2,-diphenyl-1-picryl-hydrazine), which has a yellow color, and the absorbance at 515 nm decreases as the radical decreases, this change being determined spectrophotometrically.

The reduction in the DPPH radical by substances with antioxidant activity is primarily based on an electron transfer mechanism (Single Electron Transfer or SET) and to a lesser extent on a hydrogen atom transfer mechanism (HAT) [25]. The percentages of DPPH radical inhibition at various concentrations of the samples were fitted to a dose–response curve, and from the equation of the curve the IC_50_ value was calculated, referring to the amount of substance that causes a 50% inhibition of DPPH radicals.

### 3.5. Acaricidal Studies

Mites and Bees

The protocol described below is a modification of an earlier-published procedure [6]. The *V. destructor* mites were collected from the colonies of *A. mellifera* with sister queens. For mite collection, an apparatus method was used, introduced by Ariana et al. [26]. Approximately 1000 infected adult honeybees were transferred into a wire-screen cylinder, directly from bee frames. The cylinder was then placed inside a second Plexiglass cylinder. CO_2_ was released for 5 min with a flow rate of 5 L/min inducing anesthesia to the mites as well as to the bees. Then, the apparatus was shaken several times in order to separate the mites from the bees. The wire-screen cylinder was taken out of the apparatus and the bees were returned to their mother colony where, a few minutes later, they all recovered from the effect of the anesthesia. By this method, more than 80% of the mites were separated from the bees, falling to the bottom of the outer cylinder. The mites were collected and placed into test vials. No mortality of bees was observed after repeated trials. The mortality of mites observed was less than 0.1%.

### 3.6. Screening Tests

Screening of the synthetic analogs was performed as in the study of costic acid described previously [6]. The experiment was conducted in five replications under laboratory conditions in a completely randomized design. The isolated *Varroa destructor* were immediately placed in groups of five at the bottom of 35 mL glass vials with the help of a stereoscope. The compounds studied were used to make 10 mg/mL acetone solutions, which were placed on filter paper fitted to the caps. Doses of 60 μL, chosen as the optimal dose for costic acid activity, were used for measurements [6]. Acetone (60 μL) was applied as a control in some vials (five replicates). Nitrogen gas was used to remove acetone from the vials and 20 μL H_2_O was added to maintain the necessary moisture levels. The vials with *Varroa destructor* were then sealed and were incubated at 32 °C. The mortality of mites was recorded in two-hour time intervals under a stereoscope binocular set.

### 3.7. Data Analysis

Graphs and statistical analysis were performed using Statistica software, version 7.1 (Stat Soft Inc., Tulsa, OK, USA) [27], while *t*-tests were performed using MS Office Excel 2013.

We performed a *t*-test (one-tailed, equal variance) for each compound with its control and the pairs between the parental compound (lactone) and the corresponding synthetic analog (acid).

In the first *t*-tests, statistical significance is evident after 6–8 h for the two lactones (*p* 0.02–0.07) and after 12 h for the two acids (*p* 0.01–0.04). These data provide evidence that the natural product’s acaricidal activity is expressed faster than that of the synthetic congeners.

In the pairs between the various compounds, no statistically significant differences appear, as indicated by the average *p* values in these comparisons (see Supplementary Information).

## 4. Conclusions

The synthetic analog of a natural product with acaricidal activity against *Varroa destructor* led to the identification of a group of natural products and the subsequent synthesis of a second series of active synthetic acaricides. The sequence of studies of the natural products and their synthetic congeners led to meaningful conclusions regarding the “pharmacophore” group in the structural framework of the acaricides.

## Figures and Tables

**Table 1:** NMR spectroscopic data (500 MHz, CDCl_3_) of alantolactone.

#	13C	HSQC	δ ^1^H	Multiplicity, J (Hz)	COSY	HMBC
12	170.42	H-12	-	-	-	H-13/H-7
5	149.1	H-5	-	-	-	H-7/H-4/H-14/H-15/H-9/H-1
11	139.83	H-11	-	-	-	H-13/H-6/H-7
13	121.62	H-13α	6.19 (1H)	d, J = 1.77 Hz	H-7	H-7
		H-13β	5.61 (1H)	d, J = 1.77 Hz	H-7	
6	118.76	H-6	5.14 (1H)	d, J = 4.0 Hz	H-7	H-13/H-8/H-7/H-4
8	76.42	H-8	4.81 (1H)	dt, J_1_ = 6.41, J_2_ = 3.2 Hz	H-7/H-9	H-6/H-9α
9	42.65	H-9α	2.1 (1H)	dd, J_1_ = 14.95 Hz, J_2_ = 2.75 Hz	H-9β/H-8	H-14
		H-9β	1.52 (1H)	m	H-9α/H-8	
1	41.71	H-1α	1.13 (1H)	dd, J_1_ = 14.95 Hz, J_2_ = 3.6 Hz	H-1β/H-2/H-14/H-3	H-2/H-14
		H-1β	1.52–1.6 (1H)	m	H-1α/H-3/H-14/H-2	
7	39.48	H-7	3.57 (1H)	m	H-13/H-6/H-8	H-13/H-9α/H-6
4	37.58	H-4	2.45 (1H)	m	H-15/H-2	H-6/H-1β/H-2/H-14/H-15
2	32.7	H-2	1.52–1.6 (2H)	m	H-3/H-4/H-14/H-1	H-4/H-3/H-1/H-14/H-15
10	32.65	H-10	-	-	-	H-6/H-8/H-9/H-4/H-2/H-1/H-14
14	28.55	H-14	1.19 (3H)	s	H-1/H-2/H-3	H-9/H-2/H-1/H-15
15	22.52	H-15	1.09 (3H)	d, J = 7.58 Hz	H-4/H-3	H-4/H-2/H-6
3	16.73	H-3α	1.43 (1H)	m	H3β/H-15/H1/H-2	H-4/H-15/H-2/H-1
		H-3β	1.81 (1H)	m	H-3α/H-2/H-15/H-1	

**Table 2:** NMR spectroscopic data (500 MHz, CDCl_3_) of isolantolactone.

#	13C	HSQC	δ 1H	Multiplicity, J (Hz)	COSY	HMBC
12	170.59	H-12	-	-	-	H-13/H-7
4	148.94	H-4	-	-	-	H-15/H-3/H-5/H-2/H-6/H-9α
11	142.21	H-11	-	-	-	H-13/H-7/H-9α/H-6α
13	120	H-13α	6.12 (1H)	d, J = 0.98 Hz	H-13β/H-7	H-7
		H-13β	5.58 (1H)	d, J = 0.98 Hz	H-13α/H-7	
15	106.59	H-15α	4.76 (1H)	q, J = 1.52 Hz	H-15β/H-5/H-3	H-3/H-5
		H-15β	4.43 (1H)	q, J = 1.52 Hz	H-15α/H-5/H-3/H-6β	
8	76.78	H-8	4.49 (1H)	td, J_1_ = 4.94 Hz, J_2_ = 1.58 Hz	H-7/H-9	H-13/H-7/H-6/H-9α
5	46.2	H-5	1.84 (1H)	br.d, J_1_ = 12.65 Hz	H-6α, H-15	H-15/H-3/H-6/H-14/H-2/H-9α
1	42.18	H-1α	1.24 (1H)	td, J_1_ = 13.85 Hz, J_2_ = 12.4 Hz	H-1β/H-2	H-2/H-14/H-3/H-9β
		H-1β	1.54 (1H)	m	H-1α/H-2	
9	41.36	H-9α	2.19 (1H)	dd, J_1_ = 15.6 Hz, J_2_ = 1.66 Hz	H-9β/H-8	H-1/H-14
		H-9β	1.49 (1H)	dd, J_1_ = 15.6 Hz, J_2_ = 4.72 Hz	H-9α/H-8/H-14	
7	40.52	H-7	2.96 (1H)	ddd, J_1_ = 11.96 Hz, J_2_ = 6.54 Hz, J_3_ = 5.47 Hz	H-8/H-6/H-13	H-13/H-9α/H-6
						
3	36.8	H-3α	2.0 (1H)	td, J_1_ = 12.82 Hz, J_2_ = 5.81 Hz	H3β/H2/H-15	H-15/H-2/H-1
		H-3β	2.33 (1H)	m	H-3α/H-2/H-1β	
10	34.26	H-10	-	-	-	H-8/H-9/H-5/H-6/H-14/H-1
6	27.46	H-6α	1.38 (1H)	dt, J_1_ = 13.88 Hz, J_2_ = 12.36 Hz	H-6β/H-7/H-5	H-8/H-7/H-5/H-13
		H-6β	1.73 (1H)	ddd, J_1_ = 13.99 Hz, J_2_ = 7.08 Hz, J_3_ = 2.67 Hz	H-6α/H-7	
2	22.67	H-2	1.55–1.58 (2H)	m	H-1/H3	H-3/H-15α/H-1α
14	17.64	H-14	0.82 (3H)	s	H-9β	H-9/H-5/H-1

**Table 3:** NMR (500 MHz, CDCl_3_) of dihydro-alantolactone.

#	13C	HSQC	δ ^1^H	Multiplicity, J (Hz)	COSY	HMBC
12	179.21	H12	-	-	-	H13, H11, H7
5	150.64	H5	-	-	-	H7, H4, H9a, H1a, H15, H14
6	115.55	H6	5.17 (1H)	d, J = 3.3 Hz	H7	H8, H7, H11, H4
8	76.93	H8	4.74 (1H)	dt, J_1_= 5.5 Hz, J_2_= 2.75 Hz	H7, H9a, H9b	H6, H9a
9	42.94	H9a	2.11 (1H)	dd, J_1_ = 14.73 Hz, J_2_ = 3.21 Hz	H8	H14
		H9b	1.52 (1H)	dd, J_1_ = 15.03 Hz, J_2_ = 2.43 Hz	H8	
1	42.26	H1a	1.6 (1H)	m	H1b, H1a, H2a, H2b	H14, H9b
		H1b	1.11 (1H)	dd, J_1_ = 12.3 Hz, J_2_ = 3.96 Hz		
11	40.36	H11	2.88 (1H)	dq, J_1_ = 8.67 Hz, J_2_ = 7.31 Hz	H13, H7	H7, H13, H6
7	38.77	H7	3.03 (1H)	ddd, J_1_ = 8.78 Hz, J_2_ = 5.63 Hz, J_3_ = 3.25 Hz	H6, H8, H11	H11, H9a, H13
4	38.53	H4	2.49 (1H)	m	H15, H3	H6, H15, H3
10	33.06	H10	-	-	-	H6, H8, H9a, H9b, H14
3	32.92	H3	1.57 (2H)	m	H4, H2b, H2a	H1a, H15, H4
14	28.72	H14	1.24 (3H)	s	-	H9b, H9a
15	23.04	H15	1.13 (3H)	d, J_1_ = 7.59 Hz	H4	H4
2	16.88	H2	1.43 (1H)	dquintet, J_1_ = 1.38 Hz, J_2_ = 3.56 Hz	H2b, H3	H4, H3, H1b
			1.83 (1H)	m	H2a, H3	
13	10.69	H13	1.22 (3H)	d, J = 7.31 Hz	H11	H11

**Table 4:** NMR (500 MHz, CDCl_3_) of dihydro-isoalantolactone.

#	13C	HSQC	δ ^1^H	Multiplicity, J (Hz)	COSY	HMBC
12	179.42	H12		-		H11, H13, H7
4	149.43	H4		-		H15a, H15b, H3a, H3b, H5, H6a, H2, H9a
15	106.42	H15a	4.77 (1H)	td, J_1_ = 1.53 Hz, J_2_ = 1.53 Hz	H15b, H3, H5	H3a, H3b, H5
		H15b	4.48 (1H)	td, J_1_ = 1.51 Hz, J_2_ = 1.5 Hz	H15a, H3, H5	
8	77.85	H8	4.46 (1H)	td, J_1_ = 4.29 Hz, J_2_ = 1.85 Hz	H9b, H7	H9a, H6a, H6b
5	46.57	H5	1.78 (1H)	dd, J_1_ = 12.4 Hz, J_2_ = 1.2 Hz	H6, H15a, H15b	H15a, H15b, H9a, H6a, H6b, H14
1	42.28	H1a	1.53 (1H)	M	H1b, H2	H14
		H1b	1.24 (1H)	M	H1a	
11	41.79	H11	2.8 (1H)	dq, J_1_ = 7.2 Hz, J_2_ = 7.01 Hz	1.22 (H13), 2.38(H7)	H13, H7
9	41.63	H9a	2.16 (1H)	dd, J_1_ = 15.32 Hz, J_2_ = 1.92 Hz	H9b, H8	H7
		H9b	1.46 (1H)	dd, J_1_ = 15.4 Hz, J_2_ = 4.25 Hz	H9a, H8	
7	40.36	H7	2.38 (1H)	Dtd, J_1_ = 12.4 Hz, J_2_ = 6.15 Hz, J_3_ = 4.2 Hz	H6b, H6a, H11, H8	H13, H9a, H11, H6
3	36.8	H3a	2.33 (1H)	m	H2, H3b	H15a, H15b, H2
		H3b	1.99 (1H)	ddd, J_1_ = 13.42 Hz, J_2_ = 12.4 Hz J_3_ = 6.61 Hz	H2, H3a, H15a, H15b	
10	34.86	H10	-	-	-	H14, H6a, H9a, H9b, H5, H6b
2	22.72	H2	1.58 (2H)	m	H3b, H1b	H3b, H1b
6	21.29	H6a	1.55 (1H)	m	H7, H6b, H5	H11, H7, H5, H8
		H6b	1.16 (1H)	dt, J_1_ = 13.7 Hz, J_2_ = 12.4 Hz	H5, H7, H6a	
14	17.8	H14	0.8 (3H)	s	-	H9a, H5, H9b, H1a
13	9.3	H13	1.22 (3H)	d, J = 7.2 Hz	H11	H11

**Table 5:** List of synthetic derivatives, their diastereomeric ratios, and the method of determination.

Derivative	Percentage ^a^	Diastereomeric Ratio	Means of Stereochemical Assignments
**5*R***	65%	1.86/1.00	^1^H NMR, GC-MS
**5*S***	35%		^1^H NMR, GC-MS
**6*R***	55%	1.22/1.00	^1^H NMR, GC-MS
**6*S***	45%		^1^H NMR, GC-MS
**7*R***	84%	5.25/1.00	^1^H NMR, GC-MS
**7S**	16%		^1^H NMR, GC-MS
**8*R***	100%		^1^H NMR, GC-MS
**8*S***	0		
**9*R***	90%	9.00/1.00	^1^H-^1^H NOESY, GC-MS
**9*S***	10%		^1^H-^1^H NOESY, GC-MS
**10*R***	68%	2.13/1.00	^1^H-^1^H NOESY, GC-MS
**10*S***	32%		^1^H-^1^H NOESY, GC-MS
**11*R***	100%		^1^H-^1^H NOESY, GC-MS
**11*S***	0		**-**
**12*R***	100%		^1^H-^1^H NOESY, GC-MS
**12*S***	0		

^a^ For GC-MS chromatographs of diastereomeric mixtures, see Supplementary Information.

**Table 6:** IC_50_ and R^2^ values of the samples exhibiting antioxidant activity.

Sample	IC_50_ (mg/mL)	R^2^
Alantolactone amine (**8**)	4.17	1.00
Hexane extract	10.46	1.00
Methanolic extract	0.56	1.00

## Data Availability

The original contributions presented in this study are included in the article/supplementary material. Further inquiries can be directed to the corresponding author.

## Supplementary Materials

The following supporting information can be downloaded at https://www.mdpi.com/article/10.3390/plants15020310/s1; Tables S1.1–S1.12: Synthetic Procedures and Analytical Data of Alantolactone and Isoalantolactone Derivatives. Figures S2.1–S2.75: Exemplary copies of NMR spectra of compounds. Figures S2.76–S2.86: GC-MS chromatograms of diastereomeric mixtures. Figures S3.1–S3.2: Biological assays-Acaricidal Studies. Tables S3.1–S3.5 Biological assays-Acaricidal Studies. Figures S3.3–S3.5 Biological assays-Antioxidant Studies.
