# Supplementary material for: From Nature to Synthesis and Vice Versa: Costic Acid Analogs with Acaricidal Activity Against the Bee Parasite Varroa destructor
Source: Plants (Basel). 2026 Jan 20;15(2):310. doi: 10.3390/plants15020310 (PMC12845287; doi:10.3390/plants15020310)
Supplement: Supplementary file 1 [file plants-15-00310-s001.zip › plants-4056404-supplementary.pdf]

From Nature to Synthesis and *vice versa.* Costic Acid Analogs with Acaricidal Activity against the Bee Parasite *Varroa destructor*.

E. Papastefanaki, A. Spyros, D. Isaakidis, M. Kalivretaki, D. Moraiti, N. Stratigakis, D. Ghanotakis and H. E. Katerinopoulos*

Department of Chemistry, University of Crete, Voutes Campus, 71003, Heraklion, Crete, Greece.

E-mail: kater@chemistry.uoc.gr; Fax: +30 2810 545166; Tel: +30 2810 545026

August 4, 2025

Supplementary Information

Table of Contents

1. Synthetic Procedures and Analytical Data 2

A. Synthesis of Alantolactone Derivatives 2

B. Synthesis of Isoalantolactone Derivatives 13

2. Exemplary copies of NMR spectra of compounds 22

3. Biological assays 63

A. Acaricidal Study 63

B. Antioxidant Study 66

1. Synthetic Procedures and Analytical Data

A. Synthesis of Alantolactone Derivatives

1.1** Synthesis of 2-(5,8a-dimethyl-2-oxo-2,3,3a,5,6,7,8,8a,9,9a-decahydronaphtho[2,3-b]furan- 3-yl) acetonitrile **(5)

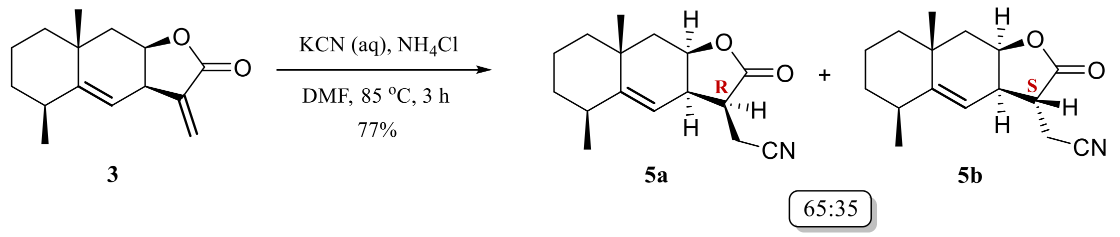

In a 25 ml round bottom flask, 105 mg (0.45 mmol, 1 eq) of alantolactone was transferred and then 1.1 ml (0.4 M) of N,N-dimethylformamide (DMF) was added. This was followed by the addition of 60 mg (0.92 mmol, 2.04 eq) of KCN dissolved in 0.3 ml (3.1 M) of H_2O, under stirring, and then 55 mg (1.03 mmol, 2.3 eq) of NH_4Cl was added. The flask was transferred to an oil bath and after being connected to a cooler the solution was heated to 80°C for 3 hours under stirring. A GC-MS spectrum confirmed the formation of the desired product and the solution underwent further workup. To the round-bottomed flask with the solution was added 20 ml of CH_2Cl_2 and stirred vigorously for 2 min. This was followed by their careful and quantitative transfer to a 100 ml extraction funnel, where extractions were carried out with H_2O (5x5 ml). The organic phase was collected, dried with anhydrous Na_2SO_4, and then the solvent was removed under vacuum. The product **5** (2-(5,8a-dimethyl-2-oxo-2,3,3a,5,6,7,8,8a,9,9a-decahydronaphtho[2,3-b]furan- 3-yl) acetonitrile) was obtained without further purification as a yellow oil, as a mixture of **5a**, **5b**. Yield: 90 mg, 77%.

GC-MS *m/z*: 259 $[M]^+$. HRMS (ESI) *m/z* calculated for $C_{16}H_{21}NO_2$ $[M+Na]^+$ 282.1464 amu, found 282.1466 amu.

Table S1.1: Spectroscopic Data from NMR (500MHz, $CDCl_3$) of the main diastereoisomer **5a**.

#	^{13}C	HSQC	δ ^1H	Multiplicity, J(Hz)	COSY	HMBC	^1H-^1H NOESY
1	41.7	H1a<br>H1b	1.14 (1H) pseudo-axial<br>1.57 (1H) pseudo-equatorial	m<br>m	H1b, H2<br>H1a, H2	H2, H14	H1b, H2, H3b<br>H1a, H2
2	32.7	H2	1.57 (2)	m	H3a , H3b, H1b	H13a, H13b, H15, H1b, H3a	H3b, H3a, H4
3	16.7	H3a<br>H3b	1.44 (1H) pseudo-equatorial<br>1.82 (1H) pseudo-axial	m (*J_1= 13.83Hz*)<br>m	H3b, H14<br>H3a, H14	H4, H2, H15<br>-	H3b, H2<br>H2, H3a, H14, H15
4	38.5	H4	2.54 (1H)	m	H15, H2	H15, H6	H2, H15
5	153.6	5	-	-	-	H6, H7, H4, H9b, H9a, H14, H15	-
6	112.8	H6	5.22 (1H)	d, *J=3.30Hz*	H7	H8, H7, H11, H4	H7, H4, H15
7	37.3	H7	3.29 (1H)	ddd, *J_1= 8.41Hz, J_2= 5.60Hz, J_3= 3.40Hz,*	H11, H8, H6	H11, H13a, H13b, H9	H6, H8, **H11**, H13a
8	77.4	H8	4.83 (1H)	dt, *J_1 =5.4Hz, J_2 =2.76Hz*	H9a, H9b, H7	H6, H9b	H7, H11, H9b, H9a
9	42.5	H9a<br>H9b	1.58 (1H) pseudo-axial<br>2.14(1H) pseudo-equatorial	m<br>dd, *J_1=14.91Hz, J_2=3.20Hz*	H8, H9b<br>H8, H9a	H14	H8, H9b<br>H9a, H14, H8
10	33.1	-	-	-	-	H4, H14, H1b,H2, H6, H9b, H8	-
11	42.8	H11	3.18 (1H)	ddd, *J_1=8.41Hz, J_2= 11.37Hz, J_3=4.32Hz*	H7, H13b, H13a	H6, H7, H13a , H13b	**H7**, H13a, H13b
12	174.7	12	-	-	-	H7, H11, H13a, H13b	-
13	14.8	H13a<br>H13b	2.88 (1H)<br>2.46 (1H)	dd, *J_1=17.36Hz, J_2= 4.23Hz*<br>dd, *J_1=17.41Hz, J_2=11.51Hz*	H13b, H11<br>H13a, H11	H11<br>-	H11, H13b<br>H11, H13a
14	28.63	14	1.22 (3H)	s	-	H9a	H9a , H9b, H3b, H15
15	22.9	15	1.13 (6H)	d, *J=7.57Hz*	H4, H3b, H13a, H2	H4, H2, H6	H4, H3b, H2, H3a
16	117.6	16	-	-	-	H11, H13a, H13b	-

Table S1.2: Spectroscopic Data from NMR (500MHz, $CDCl_3$) of the main diastereoisomer **5b**.

#	^{13}C	HSQC	δ ^1H	Multiplicity, J(Hz)	COSY	HMBC	^1H-^1H NOESY
1	41.7	H1a<br>H1b	1.14 (1H) pseudo-axial<br>1.57 (1H) pseudo-equatorial	dd, J_1=6.87Hz, J_2=3.54Hz	H1b, H2<br>H1a, H2	H2, H14	H1b, H2, H3b<br>H1a, H2
2	32.8	H2	1.57 (2H)	m	H3a , H3b, H1b	H15, H1b, H13a, H13b	H4, H3b, H3a
3	16.8	H3a<br>H3b	1.44 (1H) pseudo-equatorial<br>1.82 (1H) pseudo-axial	m (J_1= 13.83Hz)<br>m	H3b, H14<br>H3a, H14	H4, H2, H15<br>-	H3b, H2<br>H2, H3a, H14, H15
4	37.9	H4	2.46 (1H)	m	H15, H2	H15, H6	H2, H15
5	150.6	5	-	-	-	H7, H4, H9b, H9a, H14, H15	-
6	118.1	H6	5.23 (1H)	d, J=3.66Hz	H7	H8, H7	H7, H11, H4, H15
7	39.7	H7	3.01 (1H)	m	H8, H6, H7	H13a, H13b, H11, H9	H6, H8
8	77.3	H8	4.96 (1H)	dt, J_1=6.7Hz, J_2=6.5Hz,	H9a, H9b, H7	H6, H9b	H7, H11, H9b, H9a
9	42.1	H9a<br>H9b	1.58 (1H) pseudo-axial<br>2.08 (1H) pseudo-equatorial	m<br>dd, J_1= 14.91Hz, J_2=3.70Hz	H8, H9b<br>H8, H9a	H14	H8, H9b<br>H8, H9a, H14
10	33.0	-	-	-	-	H4, H14, H6, H9b, H1b, H2, H8	-
11	45.3	H11	2.66 (1H)	m, J_1=9.8Hz, J_2=3.6Hz	H13a, H13b	H13a, H13a, H7, H6	H13a
12	175.5	12	-	-	-	H7, H13a, H13b	-
13	19.0	H13	2.82 (1H)<br>2.68 (1H)	dd, J_1=15.36Hz, J_2= 3.67Hz<br>d, J=15.46Hz	H13b, H11<br>H13a, H11	H7<br>-	H13b, H11<br>H13a, H11
14	28.65	14	1.21 (3H)	s	-	H9b, 1.58(H9α, H1b)	H9a, H9b, H3b, H15
15	22.4	15	1.13 (3H)	d, J=7.69Hz	H4, H13b, H3a, H2	H4, H2, H6	H4, H3b, H2, H3a
16	116.9	16	-	-	-	H13a, H13b, H11	-

1.2 *Synthesis of (2-(5,8a-dimethyl-2-oxo-2,3,3a,5,6,7,8,8a,9,9a-decahydronaphtho[2,3-b]furan)-3-yl)acetic acid* ***(6)***

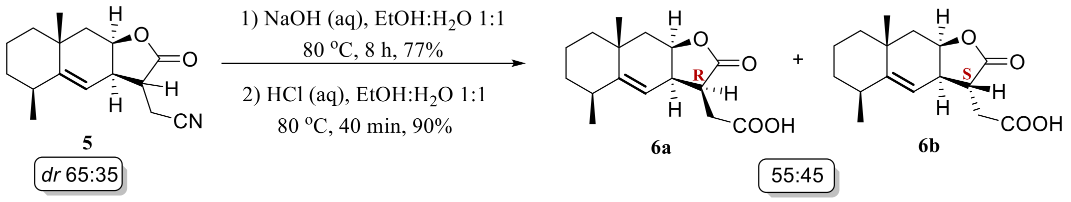

In a 10 mL round bottom flask 75 mg (0.29 mmol, 1 eq) of 2-(5,8a-dimethyl-2-oxo-2,3,3a,5,6,7,8,8a,9,9a-decahydronaphtho[2,3-b]furan-3-yl) acetonitrile **5** was transferred and 0.5 ml (0.58 M) of EtOH was added. This was followed by the addition of 59 mg (1.48 mmol, 5.09 eq) of NaOH dissolved in 0.5 ml (3.0 M) of H_2O, under stirring. The flask was connected to a condenser and placed in an oil bath. The solution was heated at 80 °C for 8 h with stirring. Completion of the reaction was ascertained by obtaining a GC-MS spectrum. This was followed by adding 3.5 ml of H_2O in the flask with the solution, acidifying it with drops of 2N HCl and then extractions were carried out with CH_2Cl_2 (3x4 ml). Then, the organic phase was collected followed by extractions with 0.1M NaOH solution (4x10 ml). The aqueous phase was collected, acidified with 2N HCl solution and then extracted with CH_2Cl_2 (4x15 ml). The organic phase was collected, dried with anhydrous Na_2SO_4 , and then the solvent was removed under reduced pressure. A mixture of product and the intermediate opened lactone product was isolated without further purification as a mixture of diastereoisomers. Yield: 62 mg, 77%.

The formation of the intermediate opened lactone was followed by transferring 29 mg (0.1 mmol, 1 eq) of it in a 10 mL round-bottomed flask. 0.5 ml of H_2O and a drop of 2N HCl were added, while 0.5 ml of EtOH was also added under stirring. Then complete dissolution of starting material was observed. The flask was attached to a condenser and the solution was heated to 80 °C for 40 min under stirring. Addition of 1 ml of H_2O and extractions with CH_2Cl_2 (4x4 ml) were followed. The organic phase was collected, dried with anhydrous Na_2SO_4, and then the solvent was removed under reduced pressure. The product **6** (2-(5,8a-dimethyl-2-oxo-2,3,3a,5,6,7,8,8a,9,9a-decahydronaphtho[2,3-b]furan)-3-yl)acetic acid) was obtained without further purification in a mixture of **6a**, **6b**. Yield: 26 mg, 90%

Along with NMR spectra, the presence of the desired product was confirmed by a TMS derivative.

GC-MS *m/z*: 332 $[M-18]^+$, 304 $[M-46]^+$. HRMS (ESI) *m/z* calculated for $C_{16}H_{22}O_4$ $[M+Na]^+$ 301.1410 amu, found 301.1412 amu.

Table S1.3: Spectroscopic Data from NMR (500MHz, $CDCl_3$) of the main diastereoisomer **6a**.

#	C	HSQC	H	Multiplicity, J(Hz)	COSY	HMBC	^{1}H-^{1}H NOESY
1	42.3	H1a<br>H1b	1.59(1H) pseudo-equatorial<br>1.12(1H) pseudo-axial	m<br>m	H1a<br>H1b	H9b, H14, H3b	H1b, H14<br>H1a, H2
2	32.88	H2	1.55 (2H)	m	H1b, H3a, H1a	H4, H1a, H3b, H15	H4
3	16.9	H3a<br>H3b	1.82(1H) pseudo-axial<br>1.43(1H) pseudo-equatorial	m<br>m	H3b, H2<br>H3a	H15, H1b, H4, H2, H1a	H3b, H14, H15
4	37.8	H4	2.47 (H1)	m	H15	H6, H15	H6,H15, H2
5	149.4	-	-	-	-	H6, H7, H4, H9a, H9b, H1a, H14, H15	-
6	119.5	H6	5.26(1H)	d, *J=3.34Hz*	H7	H8, 2H11, H4	H7, H13b, H4, H15
7	40.0	H7	2.84(1H)	s	H8, H6 , H11	H13a, H13b, H9a, H11	**H11**,H6, H8
8	77.4	H8	4.89(1H)	dt, J_1= *6.69Hz*, J_2=*3.25Hz*<br>s	H7, H9a, H9b	H6, H8, H11, H9a	H7, H9a, H9b, H11
9	42.8	H9a<br>H9b	2.08(1H) pseudo-equatorial<br>1.52(1H) pseudo-axial	dd, J_1=*14.9Hz*, J_2= *2.83Hz*<br>m	H9b, H8<br>H9a, H8	H14	H9b, H8, H14<br>H9a, H8
10	32.9	-	-	-	-	H6, H9b, H9a, H14, H8	-
11	45.3	H11	2.73 (H1)	m	H13b, H13a	H7, H13a, H13b, H6	**H7**, H8, H13
12	176.3	-	-	-	-	H13a, H7, H11, H13b	-
13	34.8	H13a<br>H13b	2.85(1H)<br>2.75(1H)	m<br>m	H13b<br>H13a	H7, H11	H11<br>H11, H6
14	28.6	H14	1.21(3H)	s	-	H9a, H9b	H9a, H3a
15	22.6	H15	1.12(3H)	d, J_1=*7.55Hz*	H4, H3a, H3b	H4, H6	H4, H6, H3a
16	178.0	-	-	-	-	H13a, H11	-

Table S1.4: Spectroscopic Data from NMR (500MHz, $CDCl_3$) of the main diastereoisomer **6b**.

#	C	HSQC	H	Multiplicity, J(Hz)	COSY	HMBC	NOESY
1	41.9	H1a<br>H1b	1.59 (1H) pseudo-equatorial<br>1.12 (1H) pseudo-axial	m<br>m	H1b, H3a<br>H1a	H14, H9b	H1b, H14<br>H1a, H2
2	32.85	H2	1.55 (2H)	m	H3a	H4, H1a, H3b, H15	H4
3	16.8	H3a<br>H3b	1.82 (1H) pseudo-axial<br>1.43 (1H) pseudo-equatorial	m<br>m	H3b, H2, H1a<br>H3a	H15, H1b, H2, H4, H1a	H3b, H14, H15<br>H3a
4	38.5	H4	2.48 (H1)	m	H15	H6, H15	H6, H15, H2
5	152.1	-	-	-	-	H6, H7, H4, H9a, H9b, H1a, H14, H15	-
6	114.4	H6	5.08 (1H)	br. s	H7	H8, H7, H11, H13a, H13b, H4	H15, H4, H13b, H13a, H7
7	37.6	H7	3.27 (1H)	br. s	H8, H6	H13a, H9a,	H6, H8
8	77.6	H8	4.81 (1H)	br. s	H7, H9a, H9b	H6, H9a	H7, H9a, H9b
9	42.75	H9a<br>H9b	2.12 (1H) pseudo-equatorial<br>1.52 (1H) pseudo-axial	dd, *J_1=14.8Hz, J_2= 2.83Hz*<br>m	H9b, H8<br>H9a, H8	H14	H9b, H8, H14<br>H9a, H8
10	33.1	-	-	-	-	H6, H9b, H9a, H14, H8	-
11	42.3	H11	3.28 (1H)	m	H13b, H13a	H7, H13a, H13b	H13a, H13b
12	177.0	-	-	-	-	H11, H13a, H13b	-
13	30.8	H13a<br>H13b	2.92 (1H)<br>2.59 (1H)	m<br>dd, *J_1=17.79Hz, J_2=9.19Hz*	H13b, H11<br>H13a, H11	H11, H7	H11, H6
14	28.7	H14	1.22 (3H)	s	-	H9b, H9a	H9a, H3a
15	23.0	H15	1.12 (3H)	d, *J_1=7.55Hz*	H4	H4, H6	H6, H4, H3a
16	177.2	-	-	-	-	H7	-

1.3 Synthesis of 3-(azidomethyl)-5,8a-dimethyl-3a,5,6,7,8,8a,9,9a-octahydronaphtho[2,3-b]furan-2(3H) -one ***(9)***

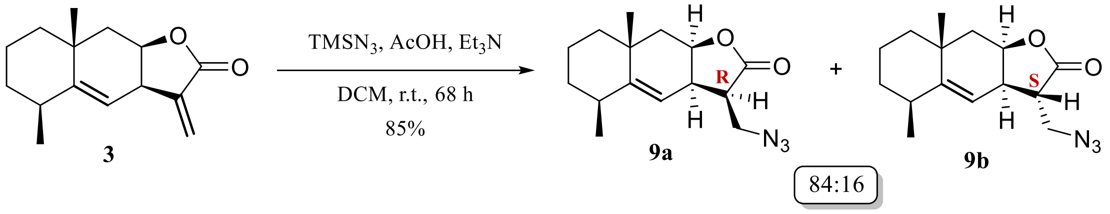

In a 10 ml two-neck round-bottomed flask in inert atmosphere, 2 ml dry CH_2Cl_2, 0.3 ml (0.92 mmol, 2.26 eq) TMSN3 and 0.13 ml (1.03 mmol, 2.27 eq) glacial AcOH were added, which were stirred for 20 min at room temperature. Then, 106 mg (0.46 mmol, 1 eq) of alantolactone **3** and 1 drop (0.36 mmol, 0.82 eq) of Et_3N were added. The reaction was left at room temperature under inert atmosphere for 68 hours. A GC-MS spectrum confirmed the formation of the desired product and the solution underwent further work up. This was followed by addition of 3 mL of H2O to the solution and extractions with EtOAc (3x4 mL). The organic phase was collected and extracted with saturated NaHCO3 solution (3x4 ml), then extracted with saturated NaCl solution (3x4 ml). Finally, the organic phase was collected, dried with anhydrous Na2SO4 , and then the solvent was removed under reduced pressure. The desired product 3-(azidomethyl)-5,8a-dimethyl-3a,5,6,7,8,8a,9,9a-octahydronaphtho[2,3-b]furan-2(3H)-one **9** was obtained without further purification in a mixture of diastereoisomers **9a**, **9b**. Yield: 106 mg, 85%

GC-MS *m/z*: 247 $[M-28]^+$, 232 $[M-43]^+$. HRMS (ESI) *m/z* calculated for $C_{15}H_{21}N_3O_2$ $[M+Na]^+$ 298.15274 amu, found 298.15273 am

Table S1.5: Spectroscopic Data from NMR (500MHz, $CDCl_3$) of the main diastereoisomer **9a**.

#	C	HSQC	H	Multiplicity, J(Hz)	COSY	HMBC	NOESY
1	42.2	H1a<br>H1b	1.60 (H1) pseudo-equatorial<br>1.13 (H1) pseudo-axial	m<br>m	H1b<br>H1a	H14, H9b, H3b	H1b, H14, H9a<br>H9b, H3b
2	32.82	H2	1.56 (H2)	m	H4, H3a	H4, H3b	H4, H1b, H3b
3	16.8	H3a<br>H3b	1.82 (H1) pseudo-axial<br>1.43 (H1) pseudo-equatorial	m<br>m	H3b, H2, H1a<br>H3a, H1a	H4, H2, H1a, H1b<br>H1a, H1b	H3b, H14, H15<br>H3a, H1b
4	38.5	H4	2.52 (H1)	m	H2, H15	H6, H15	H15, H2, H6
5	152.3	-	-	-	-	H6, H7, H4, H9a, H1a, H14 ,H15	-
6	114.1	H6	5.18 (H1)	d, J=3.14Hz	H7	H8, H7, H11, H4	H13a, H7, H4, H11
7	37.3	H7	3.19 (H1)	ddd, J_1=8.55Hz, J_2=5.57Hz, J_3=3.18Hz	H6, H8, H11	H13a, H13b, H9a,H11	H6, H8, **H11**, H9b
8	77.4	H8	4.78 (H1)	dt,J_1=5.50Hz, J_2=2.84Hz	H7, H9a, H9b	H6, H9a	H7, H11, H9a, H9b
9	42.6	H9a<br>H9b	2.12 (H1) pseudo-equatorial<br>1.53 (H1) pseudo-axial	dd, J_1=14.82Hz, J_2=3.21Hz<br>m	H8, H9b<br>H8, H9a	H14	H8, H9b, H14, H1a<br>H8, H7, H9a
10	33.1	-	-	-	-	H6, H14, H15, H9b, H1a, H4, H9a, H8	-
11	45.7	H11	3.06 (H1)	ddd, J_1=10.51, J_2=8.52Hz, J_3=4.72Hz	H13a, H13b, H7	H13a, H13b, H6 weak, H8 weak	H8, H13a, H13b, **H7**
12	175.3	-	-	-	-	H13a, H13b, H7, H11	-
13	47.8	H13a<br>H13b	3.80 (H1)<br>3.43 (H1)	dd, J_1=12.8Hz, J_2=4.70Hz<br>dd, J_1=12.8Hz, J_2=10.56HzHz	H13b, H11<br>H13a, H11	H11	H3b, H11)<br>H13a, H11
14	28.6	H14	1.22 (H3)	s	-	H9a, H9b	H1a, H3a,H9a
15	23.0	H15	1.13 (H3)	d, J=7.41Hz	H4	H4, H6, H2	H4, H3a

Table S1.6: Spectroscopic Data from NMR (500MHz, $CDCl_3$) of the main diastereoisomer **9b**.

#	C	HSQC	H	Multiplicity, J(Hz)	COSY	HMBC	NOESY
1	41.9	H1a<br>H1b	1.60 (H1) pseudo-equatorial<br>1.13 (H1) pseudo-axial	m<br>m	H1b, H3a, H14<br>H1a, H3b	H14, H9b, H3b, H3a	H1b, H14, H9a<br>H1a, H9b, H2,H3b
2	32.84	H2	1.56 (H2)	m	H4, H3a	H4, H3b, H3a weak, H1a	H4, H1b, H3b
3	16.82	H3a<br>H3b	1.82 (H1) pseudo-axial<br>1.43 (H1) pseudo-equatorial	m<br>m	H3b, H2, H1a<br>H3a, H1b	H4, H2, H1a, H1b	H3b, H14, H15<br>H3a,H1b
4	37.9	H4	2.45 (H1)	m	H2, H15	H6, H15	H15, H2, H6
5	149.7	-	-	-	-	H7, H4, H9a, H1a, H14, H15	-
6	119.0	H6	5.17 (H1)	d, $J= 3.69Hz$	H7	H8, H7, H11, H4	H7, H4, H13
7	38.54	H7	2.96 (H1)	ddd, $J_1=6.86Hz, J_2=3.70Hz, J_3=2.35Hz$	H6, H8, H11 weak	H13, H9a	H6, H8, H13
8	77.8	H8	4.89 (H1)	dt, $J_1=6.80Hz, J_2=3.24Hz$	H7, H9a, H9b	H6, H9a, H11	H7, H13, H9a, H9b
9	42.7	H9a<br>H9b	2.07 (H1) pseudo-equatorial<br>1.53 (H1) pseudo-axial	dd, $J_1=14.92Hz, J_2=3.27Hz$	H8, H9b<br>H8, H9a	H14	H8, H9b, H14, H1a<br>H8, H7, H9a, H1b
10	33.1	-	-	-	-	H6, H14, H15, H9b, H1a, H4, H9a, H8	-
11	49.3	H11	2.53 (H1)	m	H13, H8	H13, H6	H13
12	176.5	-	-	-	-	H13, H7	-
13	51.6	H13a<br>H13b	3.70 (H1)<br>3.70 (H1)	m<br>m	H13b, H11<br>H13b, H11	H11	H11<br>H11
14	28.7	H14	1.21 (H3)	s	-	H9a, H9b, H1a	H1a, H3a, H9a
15	22.6	H15	1.13 (H3)	d, $J=7.40Hz$	H4, H3a, H1a	H4, H6, H2	H4, H3a

1.4** Synthesis of (3R)-3-(aminomethyl)-5,8a-dimethyl-3a,5,6,7,8,8a,9,9a-octahydronaphtho[2,3-b]furan-2(3H)-one **(10)

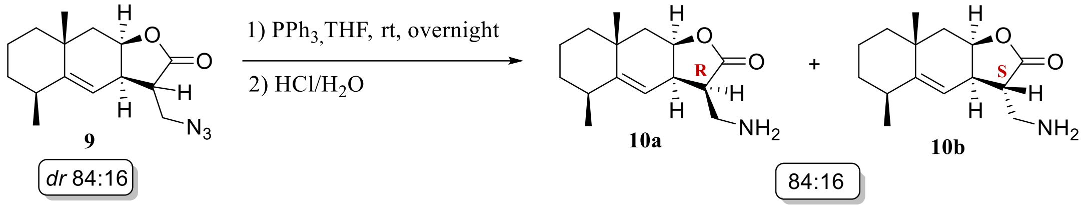

Substrate **9**, 86 mg (0.31 mmol, 1 eq), was placed in a 25 ml round bottom flask and 2.2 ml (0.14 M) of THF was added. Then, 98 mg (0.37 mmol, 1.19 eq) of triphenylphosphine (PPh3) was added under stirring. The reaction was stirred at room temperature overnight. Completion of the reaction was confirmed by obtaining a GC-MS spectrum and the solution was further processed. This was followed by removal of THF from the solution under reduced pressure and addition of diethyl ether (Et2O). Next, the solution was transferred in a 50 ml extraction funnel and extractions with H2O took place, while the H2O was acidified with drops of 2N HCl solution, until the pH was between 2-3 (4x5 ml). The aqueous phase was then collected and 2N KOH was added until the pH became strongly basic (pH=13). Extractions with CH2Cl2 (4x20 ml) followed. Finally, the organic phase was collected, dried with anhydrous Na2SO4 , and the solvent was removed under vacuum. The desired product **10a** was isolated by column chromatography (eluent solvent: MeOH). Due to formation of triphenylphosphine oxides as byproduct the yield of isolated pure product was significantly lower than the yield of conversion (~100%). Yield: 10 mg, 13 %.

GC-MS *m/z*: 249 $[M]^+$, 234 $[M-15]^+$. HRMS (ESI) *m/z* calculated for $C_{15}H_{23}NO_2$ $[M+H]^+$ 250.18016 amu, found 250.17972 amu.

Table S1.7: Spectroscopic Data from NMR (500MHz, $CDCl_3$) of the main diastereoisomer **10a**.

#	^{13}C	HSQC	δ ^1H	Multiplicity, J(Hz)	COSY	HMBC	^1H-^1H NOESY
1	42.19	H1a<br>H1b	1.60 (1H) pseudo-equatorial<br>1.10 (1H) pseudo-axial	m<br>m	H1b<br>H1a	H14, H2, H9b	H1b, H14<br>H1a, H2
2	32.84	H2	1.55 (2H)	m	H4, H3a , H1	H4, H1a, H1b, H3b	H4, H1b
3	16.81	H3a<br>H3b	1.82 (1H) pseudo-axial<br>1.43 (1H) pseudo-equatorial	m<br>m	H15, H3b, H2<br>H3a, H15	H4, H1a, H1b, H2	H3b, H14<br>H3a
4	38.46	H4	2.47 (1H)	m	H15, H2	H6, H15	H2, H6, H15
5	151.22	-	-	-	-	H6, H7, H4, H9a, H1a, H2, H14, H15	-
6	114.95	H6	5.15 (1H)	d, *J=3.26Hz*	H7	H4, H7, H8, H11	H4, H7 weak, ,H13a weak, H13b weak
7	37.67	-	3.16 (1H)	ddd, J_1= *3.14Hz,* J_2= *5.48Hz,* J_3= *8,44Hz*	H6, H8, H11	H6, H11, H9α, H13α	H6, **H8**, **H11**
8	77.26	H8	4.74 (1H)	-	H7, H9a, H9b	H6, H9a	H7, H9a, H9b
9	42.69	H9a<br>H9b	2.10 (1H) pseudo-equatorial<br>1.51 (1H) pseudo-axial	dd, J_1= *14.75Hz,* J_2= *3.27Hz*<br>m	H9b, H8<br>H9a, H8	H14	H8, H9b, H14<br>H1b, H8, H9a
10	33.02	-	-	-	-	H1a, H4, H6, H8, H9a, H9b, H14	-
11	48.09	H11	2.91 (H1)	m	H7, H13a	H7, H13a	**H7**, H13
12	177.89	-	-	-	-	H7, H11	-
13	39.15	H13a<br>H13b	3.10 (1H)<br>2.96 (1H)	dd, J_1= *12.80Hz,* J_2= *7.00Hz*<br>m	H13b, H11<br>H13a	H11	-<br>-
14	28.61	H14	1.23 (3H)	s	-	H9a, H9b, H1a	H3a, H9a, H1a
15	22.87	H15	1.12 (3H)	d, *J= 7,60Hz*	H4, H3a, H3b	H4	H4
-	-	NH_2	2.16 (2H)	br. s	-	-	-

B. Synthesis of Isoalantolactone Derivatives

1.5 *Synthesis of 2-(8a-methyl-5-methylene-2-oxododecahydronaphtho[2,3-b]furan-3-yl)acetonitrile* ***(7)***

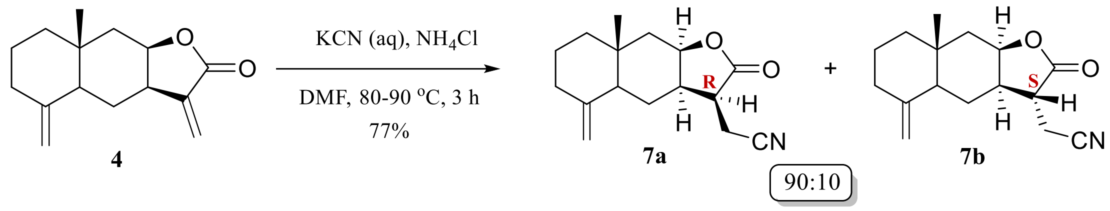

For the preparation of the product, the same experimental procedure was followed as for the preparation of the compound **5** in section **1.1**. The desired product 2-(8a-methyl-5-methylene-2-oxododecahydronaphtho[2,3-b]furan-3-yl)acetonitrile **7** was obtained without further purification as a mixture of **7a**, **7b**. Yield: 83 mg, 77%.

GC-MS *m/z*: 259 $[M]^+$, 244 $[M-15]^+$. HRMS (ESI) *m/z* calculated for $C_{16}H_{21}NO_2$ $[M+Na]^+$ 282.1464 amu, found 282.1463 amu.

Table S1.8: Spectroscopic Data from NMR (500MHz, $CDCl_3$) of the main diastereoisomer **7a**.

#	^{13}C	HSQC	δ ^1H	Multiplicity, J(Hz)	COSY	HMBC	NOESY
1	42.0	H1a<br>H1b	1.55 (1H) pseudo-equatorial<br>1.25 (1H) pseudo-axial	m<br>td, J_1=13.29Hz, J_3=5.49Hz	H1b<br>H1a	H3a, H3b, H14, H9b	H1b, H14<br>H3b, H5, H1a, H2, H9b
2	22.5	H2	1.59 (2H)	m	H3a, H3b, H1b	H3b, H1b	H3a, H3b, H1b
3	36.6	H3a<br>H3b	2.34 (1H) pseudo-equatorial<br>2.00 (1H) pseudo-axial	dq J_1= 13.20Hz, J_2= 2.03Hz<br>td, J_1= 12.87Hz, J_3= 5.65Hz	H3b, H2<br>H13a, H2, H15a, H15b	H15a, H15b, H1a, H2	H15a, H3b, H2<br>H3a, H5, H1a, H1b
4	148.6	-	-	-	-	H15a, H15b, H3a, H3b, H5, H6a, H6b, H2, H9a	-
5	46.1	H5	1.85(1H)	br. dd, J_1= 12.26Hz, J_2= 1.23Hz	H15a, H15b, H6b	H15a, H15b, H9a, H6a, H6b, H1a, H14, H3a, H3b	H7, H3b, H9b, H1b, H6a, H6b weak
6	20.6	H6a<br>H6b	1.69 (1H) pseudo-equatorial<br>1.14 (1H) pseudo-axial	ddd, J_1=13,09Hz, J_2=5.93Hz, J_3=2.47Hz<br>dt, J_1=12.80Hz, J_2=12.61Hz	H7, H5, H6b<br>H7, H5, H6a	H8, H11, H7, H5	H6b, H7, H5, H15b<br>H13b, H5, H6a, H14
7	38.7	H7	2.68 (1H)	dddd, J_1= 12.40Hz, J_2= 6.38H, J_3= 6.20Hz, J_4= 4.05Hz	H8, H1, H6a, H6b	H11, H13a, H13b, H9a, H6a, H6b	**H8**, **H11**, H5, H6a, H9b, H6b weak
8	78.3	H8	4.57(1H)	ddd, J_1= 4.07Hz, J_2= 4.07Hz, J_3=1.84Hz	H7, H9a, H9b	H9a, H6a, H7, H11	H11, H7, H9a, H9b
9	41.2	H9a<br>H9b	2.19 (1H) pseudo-axial<br>1.51 (1H) pseudo-equatorial	dd, J_1= 15.54Hz, J_2= 1.89Hz<br>dd, J_1=15.54Hz, J_2=4.33Hz	H9b, H8<br>H9a, H8	H1b, H14	H8, H9b, H14<br>H5, H14, H8, H9a
10	34.6	-	-	-	-	H9a, H9b, H5, H6a, H1b, H6b, H14	-
11	44.1	H11	3.12 (1H)	ddd, J_1=11.25Hz, J_2=4.64 J3= 6.22Hz	H13a, H13b, H7	H13a, H13b, H7, H6b	H8, **H7,** H13a, H13b
12	174.9	-	-	-	-	H11, H13a, H13b, H7	-
13	13.7	H13a<br>H13b	2.90 (1H)<br>2.53 (1H)	dd, J_1= 17.42Hz, J_2= 4.52Hz<br>dd, J_1= 17.45Hz, J_2= 11.34Hz	H13b, H11<br>H13a, H11	H11	H13b, H11<br>H13a, H11, H6a, H6b
14	17.7	H14	0.79 (3H)	s	H1b, H9b	H9a, H5, H9b, H1b	H9a, H1a, H6b
15	106.7	H15a<br>H15b	4.80 (1H)<br>4.46 (1H)	td, J_1=1.75Hz, J_2=1.47Hz<br>td, J_1=1.87Hz, J_2=1.45Hz	H15b, H5, H3b, H3a<br>H15a, H5, H3b	H3a, H3b, H5	H15b, H3a<br>H15a, H6a, H6b
16	117.43	-	-	-	-	H13a, H13b, H11	-

__1.6__ Synthesis of 2-(8a-methyl-5-methylene-2-oxododecahydronaphtho[2,3-b]furan-3-yl)acetic acid __(8)__

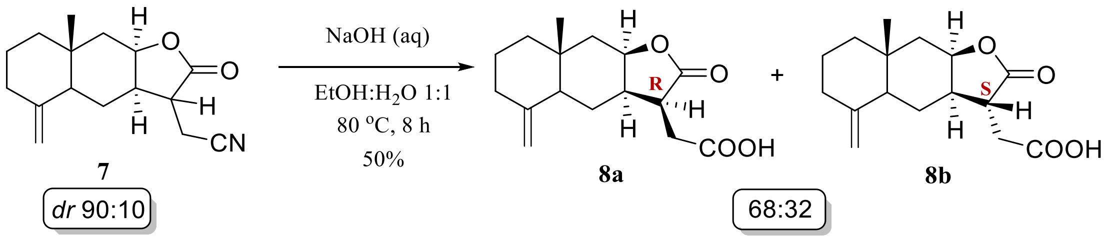

For the preparation of this acid, the same procedure was followed as for the compound **6** in section **1.2**, with the only difference being that no boiling was performed in acidic conditions to close the opened lactone ring, as such a phenomenon was not observed. The desired product 2-(8a-methyl-5-methylene-2-oxododecahydronaphtho[2,3-b]furan-3-yl)acetic acid **8** was obtained without further purification in a mixture of **8a**, **8b** with 10% by-product admixture. Yield: 40 mg, 50%.

An attempt was made to separate the two diastereomers by semi-preparative HPLC. During the analysis of a 29 mg sample with a 60:40 AcCN:H_2O system as eluent and a flow rate of 3 ml/min, 14 mg of a highly pure mixture of **8a** and **8b** was collected, free of any impurity.

The presence of the desired product was confirmed by NMR spectra and GC-MS spectra of a TMS derivative.

GC-MS *m/z*: 350 $[M]^+$. HRMS (ESI) *m/z* calculated for $C_{16}H_{21}NO_2$ $[M+Na]^+$ 301.1410 amu, found 301.1414 amu.

Table S1.9: Spectroscopic Data from NMR (500MHz, $CDCl_3$) of the main diastereoisomer **8a**.

#	^{13}C	HSQC	δ ^1H	Multiplicity, J(Hz)	COSY	HMBC	NOESY
1	41.6	H1a<br>H1b	1.53 (1H)<br>1.23 (1H)	m<br>m	H1a, H2<br>H2, H1b	H14, H3a	H1b, H14<br>H3b, H5, H2, H1a
2	22.5	H2	1.60 (2H)	m	H3a, H3b, H1b, H1a	H3b, H1b, H3a, H1a weak	H3a, H1b, H3b
3	36.3	H3a<br>H3b	2.32 (1H) pseudo-equatorial<br>1.98 (1H) pseudo-axial	m<br>m	H3b, H2<br>H3a, H2, H15a	H15, H15	H15a, H3b, H2<br>H3a, H2, H1b
4	149.0	-	-	-	-	H15a weak, H15b, H3a, H9a, H3b, H5, H2, H6b	-
5	49.3	H5	1.91(1H)	dd, J_1=12.20Hz, J_2=1.05Hz	H15b, H15a weak, H6b	H15a, H15b, H9a, H7, H6b, H6a, H14	H1b, H7, H6a, H9b, H15b
6	27.0	H6a<br>H6b	1.68 (1H) pseudo-equatorial<br>1.53 (1H) pseudo-axial	ddd, J_1=13.20Hz, J_2= 4.84Hz, J_3=2.80Hz<br>m	H5, H7, H6b<br>H7, H6a, H5	H8, H11, H5	H15b, H6b, H13b, H5, H7<br>H15b, H6a
7	37.3	H7	2.32 (1H)	m	H8, H11, H6a, H6b	H13a, H13b,H11, H9a, H6a, H6b	**H11**,**H8**, H5, H6a
8	75.7	H8	4.55 (1H)	m	H7, H9a, H9b	H11, H9a, H6a, H6b	H11, H7, H9a, H9b
9	43.7	H9a<br>H9b	2.04 (1H) pseudo-axial<br>1.49 (1H) pseudo-equatorial	dd, J_1=15.0Hz, J_2=2.49Hz<br>m	H8, H9b<br>H8, H9a	H14, H5 weak	H8, H9b, H14<br>H8, H9a, H5, H1b
10	35.1	-	-	-	-	H5, H9b, H9a, H1b, H14, H6a, H6b	-
11	42.2	H11	2.71 (1H)	m	H7, H13a, H13b	13a, H13b	**H7**, H13a, H8
12	178.2	-	-	-	-	H11, H13b, H13a	-
13	29.2	H13a<br>H13b	2,82 (1H)<br>2.71 (1H)	m<br>m	H11, H13b<br>H13a, H11	H11	H13b, H11<br>H13a
14	17.7	H114	0.88 (H)	s	-	H9a, H9b , H5, H1b	H9a, H1a, H15a, H15b weak
15	106.2	H15	4.76 (1H)<br>4.45 (1H)	dt, J_1=1.52Hz, J_2=1.48Hz<br>dt, J_1=1.50Hz, J_2=1.48Hz	H15b, H5, H3b<br>H15a, H5	H3a, H3b, H5	H3a, H15b<br>H15a, H6a, H6b, H14
16	170.8	-	-	-	-	H13a, H13b, H11	-

Table S1.10: Spectroscopic Data from NMR (500MHz, $CDCl_3$) of the main diastereoisomer **8b**.

#	^{13}C	HSQC	δ ^1H	Multiplicity, J(Hz)	COSY	HMBC	NOESY
1	42.2	H1a<br>H1b	1.53 (1H) pseudo-equatorial<br>1.23 (1H) pseudo-axial	m<br>m	H1a, H2<br>H2, H1b	H14	H1b, H14<br>H3b, H5, H2, H1a
2	22.7	H2	1.60 (2H)	m	H3a, H3b, H1b, H1a	H3b, H1b, H3a, H1a weak	H3a, H1b, H3b
3	36.7	H3a<br>H3b	2.32 (1H) pseudo-equatorial<br>1.98 (1H) pseudo-axial	m	H3b, H2<br>H3a, H2, H15a	H15, H15b	H15a, H3b, H2<br>H3a, H2, H5, H1b
4	149.1	-	-	-	-	H15a, H15b, H3a, H9a, H3b, H5, H2 ,H6b	-
5	46.4	H5	1.80 (1H)	dd, *J_1=12.27Hz, J_2= 0.97Hz*	H15a, H15b, H6a, H6b	H15a, H15b, H9a, H6b, H6a, H14	H1b, H7, H6a, H9b,H15b
6	21.2	H6a<br>H6b	1.13 (1H) pseudo-axial<br>1.53 (1H) pseudo-equatorial	m	H5, H7, H6b<br>H7, H6a, H5	H8, H11, H7, H5	H15b, H6b, H7, H5, H14<br>H15b, H6a
7	39.0	H7	2.62 (1H)	m	H8, H11, H6b, H6a	H13a, H11, H13b, H9a	H8, H5, H9b, H6a, H6b
8	78.4	H8	4.55 (1H)	m	H9a, H9b, H7	H9a, H6b	H11, H7, H9a, H9b
9	41.4	H9a<br>H9b	2.18 (1H) pseudo-axial<br>1.48 (1H) pseudo-equatorial	dd, *J_1=15.5Hz, J_2=1.81Hz*<br>m	H9b, H8<br>H8, H9a	H14	H9b, H14, H8<br>H8, H9a, H5, H1b
10	34.8	-	-	-	-	H9a, H5, H9b , H1b, H14, H6a, H6b	-
11	43.6	H11	3.21 (1H)	ddd, *J_1=9.91Hz, J_2=6.33Hz, J_3=5.1Hz*	H13a, H7, H13b	H13a, H13b, H7, H6a	H8, H13a, H13b
12	177.2	-	-	-	-	H11, H13a, H13b	-
13	29.5	H13a<br>H13b	2.94 (1H)<br>2.60 (1H)	dd, *J_1=17.83Hz, J_2=5.19Hz*<br>dd, *J_1=17.83Hz, J_2=9.80Hz*	H13b, H11<br>H13a, H11	H11	H11, H13b , weak H6a, weak H6b<br>H11, H13a, H6a weak, H6b weak
14	17.8	H14	0.78 (3H)	s	-	H9a, H5, H1b, H9b	H9a, H1a, H6a, H15a, H15b weak
15	106.6	H15	4.78 (1H)<br>4.46 (1H)	dt, *J_1=1.51Hz, J_2=1.49Hz*<br>dt, *J_1=1.46Hz, J_2=1.42Hz*	H15b, H5<br>H15a, H5	H3a, H3b, H5	H3a, H15b<br>H15a, H6a, H6b, H14
16	176.6	-	-	-	-	H13a, H13b	-

1.7 Synthesis of (3R)-3-(azidomethyl)-8a-methyl-5-methylene- decahydronaphtho[2,3-b]furan-2(3H)-one ***(11)***

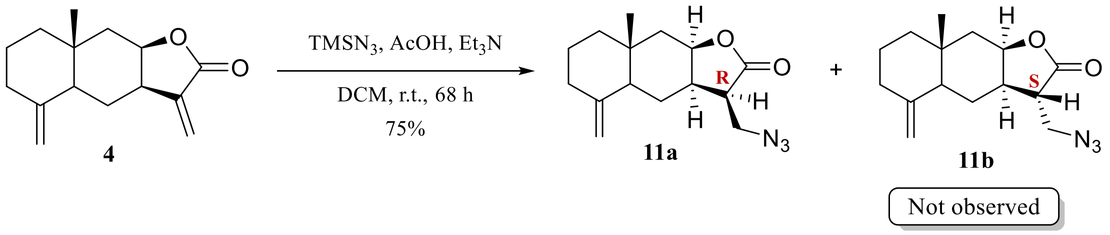

For the preparation of the compound **11**, the same experimental procedure was followed as for the preparation of compound **9** in section **1.3**. The desired product (3R)-3-(azidomethyl)-8a-methyl-5-methylene- decahydronaphtho[2,3-b]furan-2(3H)-one **11a** was obtained without further purification, while the compound **11b** was not observed. Yield: 90 mg, 75%.

GC-MS *m/z*: 275 $[M]^+$, 247 $[M-28]^+$. HRMS (ESI) *m/z* calculated for $C_{15}H_{21}N_3O_2$ $[M+Na]^+$ 298.15274 amu, found 298.15260 amu.

Table S1.11: Spectroscopic Data from NMR (500MHz, $CDCl_3$) of the main diastereoisomer **11a**.

#	^{13}C	HSQC	^{1}H	Πολλαπλότητα, J(Hz)	COSY	HMBC	^{1}H-^{1}H NOESY
1	42.2	H1	1.55 (1H) pseudo-axial<br>1.25 (1H) pseudo-equatorial	m<br>m	H1b<br>H1a, H2	H3a, H9b, H2, H14	H3a, H1b, H14<br>H1a, H9b, H5
2	22.6	H2	1.59 (2H)	m	H3a, H3b, H1b	H3b, H3a weak, H1b	H3a, H1b
3	36.7	H3a<br>H3b	2.34 (1H)<br>1.99 (1H)	dq, J_1=12.80Hz, J_2=2.00Hz<br>td, J_1=12.92Hz, J_2=6.07Hz	H3b, H1a<br>H3a, H2, H15a, H15b	H15a, H15b, H1a	H15a, H3b, H2<br>H3a
4	148.9	-	-	-	-	H15a, H15b, H3a, H3b, H9a, H5, H6a, H6b, H2	-
5	46.3	H5	3.47 (1H)	br.d, J=12.30Hz	H15a, H15b, H6b	H15a, H15b, H9a, H3a, weak, H6a, H6b, H14, H1a	H7, H6b, H6a
6	20.8	H6a<br>H6b	1.62 (1H) pseudo-equatorial<br>1.19(1H) pseudo-axial	ddd, J_1=12.88Hz, J_2=5.87Hz, J_3=2.54Hz<br>dt, J_1=12.82Hz, J_2=12.70Hz	H7, H6b<br>H6a,H7,H5	H8, H11, H7, H5	H15b, H13b, H6b, H7<br>H15b, H13b, H6a, H7
7	38.5	H7	2.57 (1H)	dddd, J_1=12.25Hz, J_2=1.79Hz, J_3=6.07Hz, J_4=4.20Hz	H8, H11, H6a, H6b	H13a, H13b, H11, H9a, H6a, H6b, H5 weak	**H8, H11**, H5, H9b, H6a, H6b
8	78.3	H8	4.51 (1H)	ddd, J_1=4.15Hz, J_2=4.15Hz, J_3=1.80Hz	H7, H9b, H9a	H9a, H6a, H6b	H11, H7, H9a, H9b
9	41.3	H9a<br>H9b	2.18 (1H) pseudo-axial<br>1.48 (1H) pseudo-equatorial	dd, J_1=15.49Hz, J_2=1.79Hz<br>dd, J_1=15.50Hz, J_2=4.34Hz	H9b, H8 weak<br>H9a, H8	H14, H1b	H8, H9b, H14<br>H8, H9a, H5
10	34.7	-	-	-	-	H8, H9a, H5, H6a, H9b, H6b, H1b, H14	-
11	47.2	H11	2.98 (1H)	ddd, J_1=10.34Hz, J_2=6.00Hz, J_3=4.90Hz	H13a, H13b, H7	H13a, H13b, H7	**H7, H8**, H13a, H13b
12	175.5	-	-	-	-	H13a, H13b, H11, H7	-
13	46.8	H13a<br>H13b	3.82 (1H)<br>3.47 (1H)	dd, J_1=12.98Hz, J_2=4.90Hz<br>dd, J_1=12.94Hz, J_2=10.40Hz	H13b, H11<br>H13a, H11	H11	H13b, H11<br>H13a, H11, H6a, H6b
14	17.7	H14	0.80 (3H)	s	-	H9a, H5, H9b, H1b	H9a, H1a
15	106.6	H15a<br>H15b	4.79 (1H)<br>4.47 (1H)	td, J_1=1.80Hz, J_2=1.30Hz<br>td, J_1=1.80Hz, J_2=1.30Hz	H15b, H5, H3b<br>H15a, H5, H3b	H3a, H3b, H5	H15b, H3a<br>H15a, H6a, H6b

1.8** Synthesis of (3R)-3-(aminomethyl)-8a-methyl-5-methylenedecahydronaphtho[2,3-b]furan-2(3H)-one **(12)

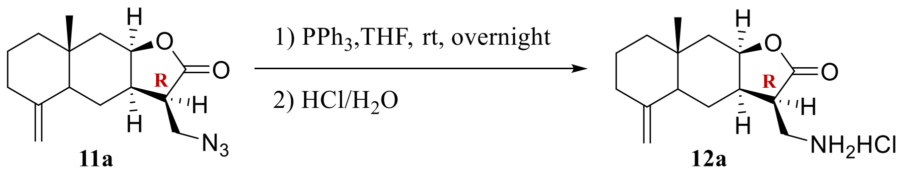

In a 25 ml round bottom flask 82 mg (0.30 mmol, 1 eq) of substrate **11a** and 2.2 ml (0.14 M) THF were added. Then, 95 mg (0.36 mmol, 1.21 eq) of triphenylphosphine (PPh_3) was added under stirring. The reaction was stirred at room temperature overnight. Conversion of the starting material was confirmed by GC-MS. Next, petroleum ether was added to the reaction flask and successive concentration took place under reduced pressure, in order to remove THF from the solution. This was followed by transferring the solution to a 50 ml extraction funnel and extracting with H_2O, which was acidified with drops of 2N HCl solution, until the pH was between 1-2 (3x5 ml). The resulting organic phase was collected, dried with anhydrous Na_2SO_4, and then the solvent was removed under vacuum. NMR spectra, GC-MS, and silica gel thin layer chromatography analysis confirmed the existence of the desired product as hydrochloride salt. The white solid product **12a** was obtained after washing with ethyl acetate. While the conversion of the reactant was almost 100%, the amount of the desired product **12a** isolated was 10 mg, with impurities of triphenylphosphine-oxides and triphenylphosphine.

The acidified aqueous phase was further treated with 2N KOH, until the pH became strongly basic (pH=12-13), followed by extractions with CH_2Cl_2 (4x20 ml), collection of the organic phase, drying with Na_2SO_4 and removal of the solvent under reduced pressure. Spectral analysis of the contents of the organic layer confirmed the absence of the desired product.

GC-MS *m/z*: 232 $[M-17]^+$, 247 $[M-28]^+$. HRMS (ESI) *m/z* calculated for $C_{15}H_{24}NO_2Cl$ $[M+H]^+$ 250.18016 amu, found 250.18019 amu.

Table S1.12: Spectroscopic Data from NMR (500MHz, $CDCl_3$) of the main diastereoisomer **12a**.

#	^{13}C	HSQC	^{1}H	Πολλαπλότητα, J(Hz)	COSY	HMBC	^{1}H- ^{1}H NOESY
1	43.1	H1a<br>H1b	1.50 (1H) pseudo-axial<br>1.30 (1H) pseudo-equatorial	m<br>td, J_1=12.87Hz, J_2=4.67Hz	H1b, H2a<br>H1a, H2b	H3a, H2, H14	H14, H1b, H9a<br>H1a, H5, H9b
2	23.8	H2	1.59 (1H)<br>1.54 (1H)	m<br>m	H2b, H1b<br>H3a, H1b	H3b, H1b, H5	H3a<br>H3a
3	37.8	H3a<br>H3b	2.30 (1H) pseudo-equatorial<br>2.01 (1H) pseudo-axial	br. *d, J=12.88Hz*<br>td, J_1=13.00Hz, J_2=5.31Hz	H3b, H2<br>H15a, H15b, H3a	H15a, H15b, H1a	H15a, H3b, H2, H1a<br>H3a, H5, H6a, H2b
4	150.5	-	-	-	-	H15a, H15b, H3a, H3b, H5, H2a, H6a	-
5	47.3	H5	1.85 (1H)	br.d, *J= 12.15Hz*	H15a, H15b, H6a, H6b	H15a, H15b, H3a, H9a, H1a, H6a, H6b, H14	H7, H6a, H3b
6	22.0	H6a<br>H6b	1.28 (1H) pseudo-equatorial<br>0.91 (1H) pseudo-axial	m<br>dt, J_1=J_2=12.68Hz	H6b, H5, H7<br>H6a, H5, H7	H8 ,H11, H7, H5	H6b, H7, H13b, H15b, H5<br>H6a, H13b, H15b
7	39.5	H7	2.67 (1H)	m	H8, H11, H6a, H6b	H13a, H13b, H9a, H6b	**H8, H11**, H5, H9b, H6a
8	80.0	H8	4.56 (1H)	br. s	H7, H9a, H9b	H9a, H6a, H6b	H11, H7, H9a, H9b
9	42.2	H9a<br>H9b	2.07 (1H) pseudo-axial<br>1.56 (1H) pseudo-equatorial	br. d, J_1=15.63Hz<br>m	H9b, H8<br>H9a, H8, H14	H1b, H14	H8, H9b, H1b, H14<br>H1b, H9a, H7, H8
10	35.7	-	-	-	-	H8, H9a, H5, H9b, H14, H1b	-
11	49.5	H11	3.14 (1H)	m	H13a,H13b, H7	H7, H13a, H13b	H13a, **H7, H8**
12	178.1	-	-	-	-	H13a, H13b, H11, H7	-
13	39.1	H13a<br>H13b	3.47 (1H)<br>3.23 (1H)	ddd, J_1=12.74Hz, J_2=7.45Hz, J_3=5.49Hz<br>*m*	H13b, H11<br>H13a, H11	H11, H6a	H13b, H11<br>H13a, H6a, H6b
14	18.2	H14	0.70 (1H)	s	H9b	H9a, H9b, H1b, H5	H9a, H1a, H6b, H2
15	107.0	H15a<br>H15b	4.70 (1H)<br>4.17 (1H)	br. s<br>br. s	H15b, H5, H3b<br>H15a, H5, H3b	H3a, H3b, H5	H15b, H3a<br>H15a, H6a, H6b

2. Exemplary copies of NMR spectra of compounds

Alantolactone ***(3)***

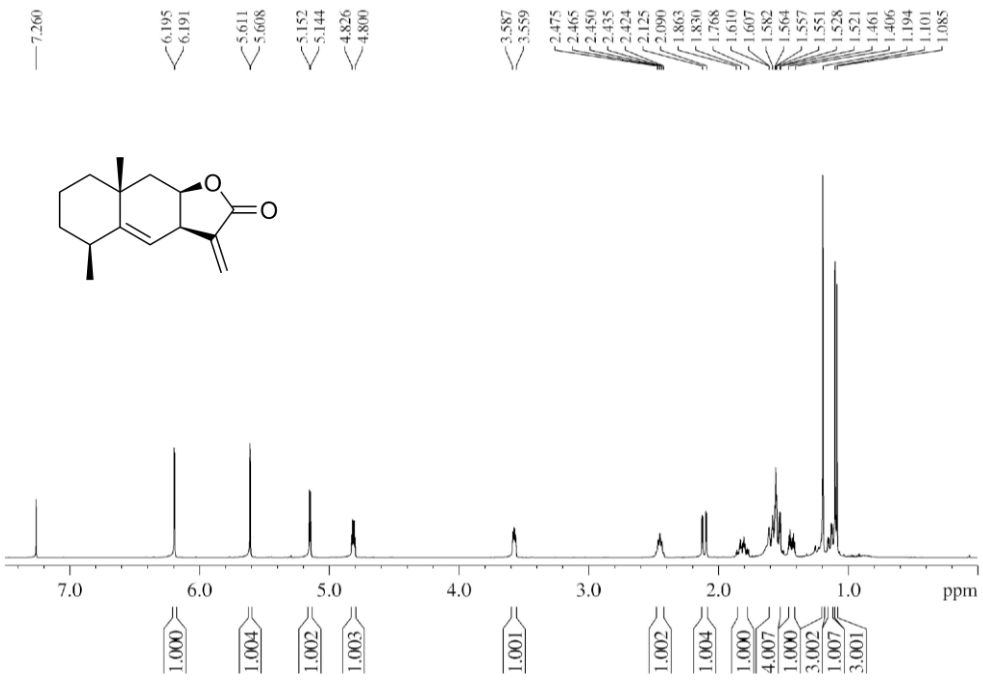

Figure S2.1: ^{1}H-NMR spectrum (500 MHz, $CDCl_3$) of alantolactone **(3).**

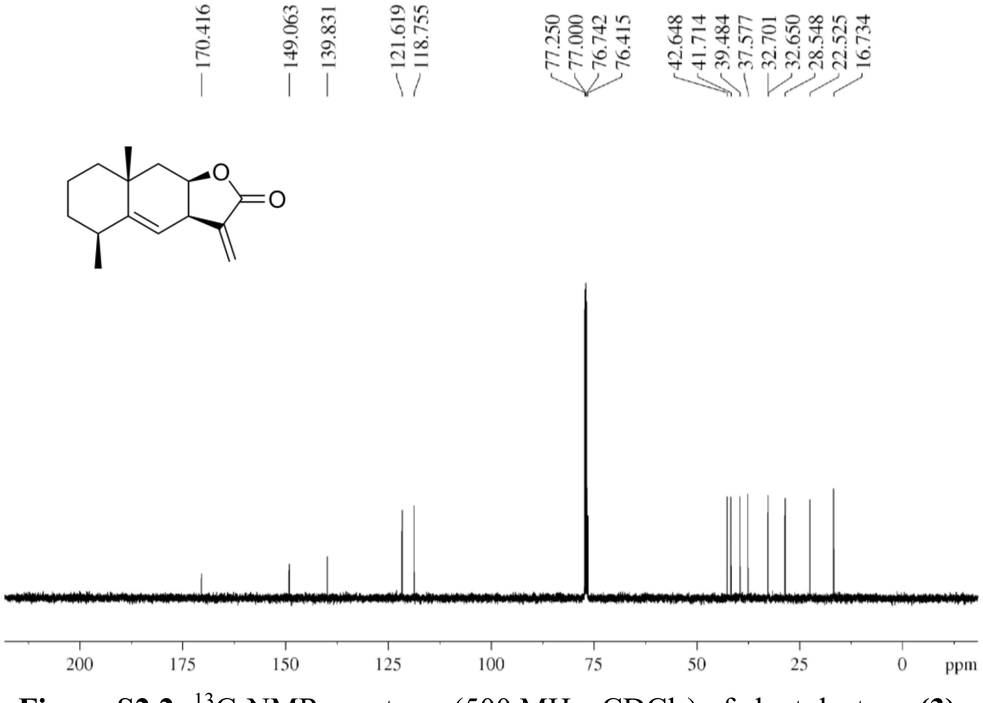

Figure S2.2: ^{13}C-NMR spectrum (500 MHz, $CDCl_3$) of alantolactone **(3).**

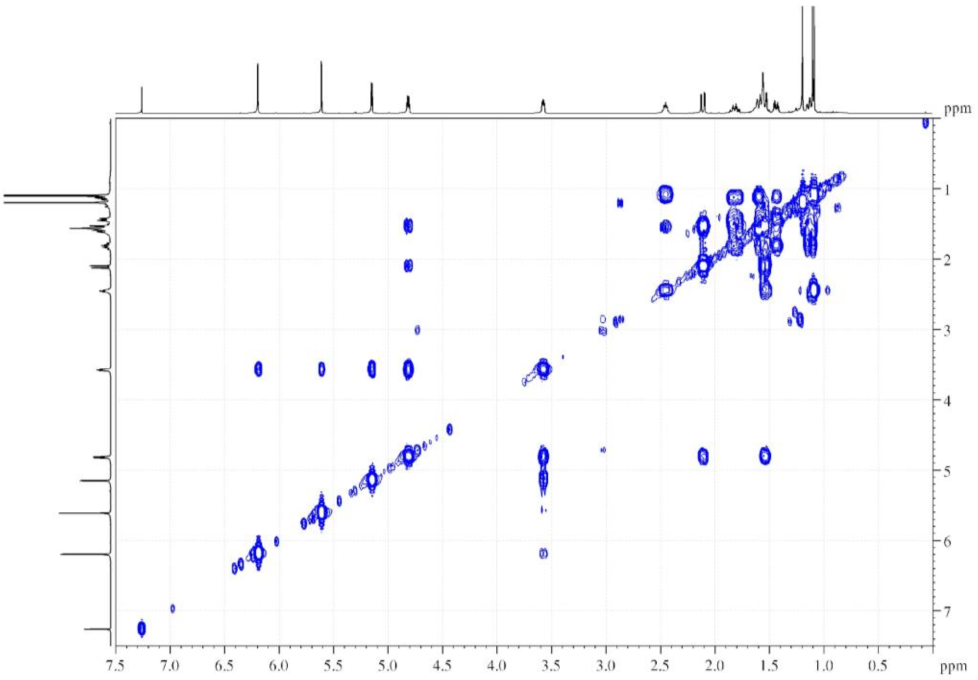

Figure S2.3: COSY spectrum (500 MHz, $CDCl_3$) of alantolactone **(3)**.

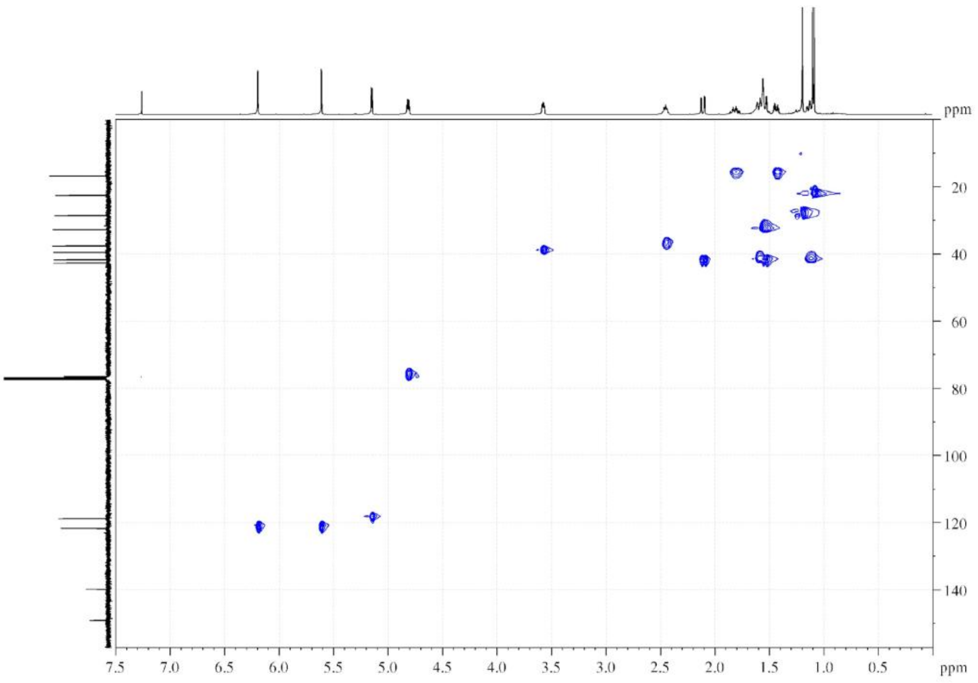

Figure S2.4: HSQC spectrum (500 MHz, $CDCl_3$) of alantolactone **(3)**.

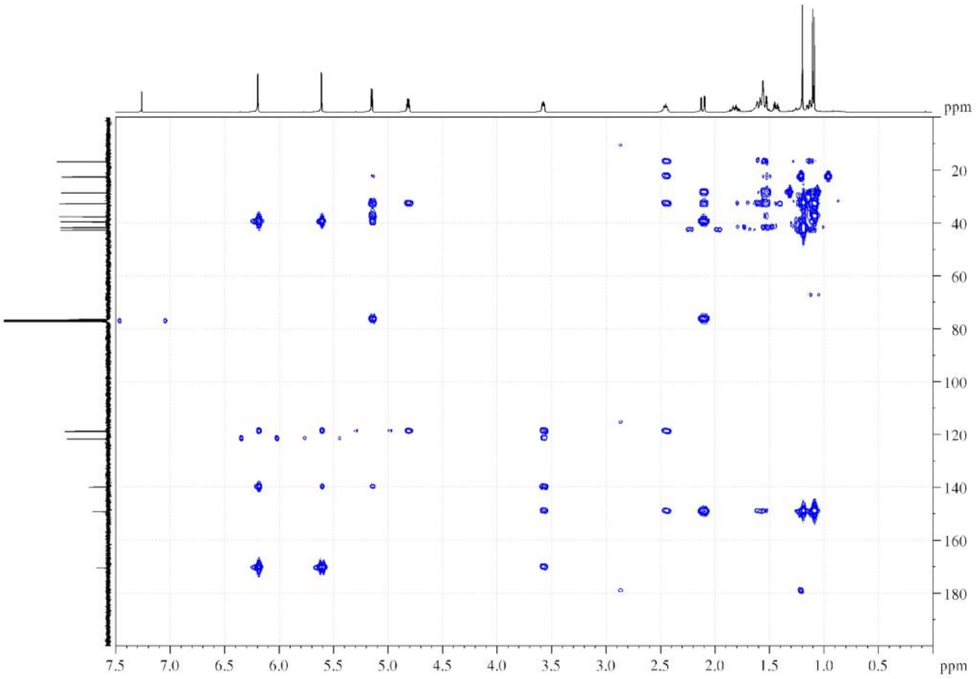

Figure S2.5: HMBC spectrum (500 MHz, $CDCl_3$) of alantolactone **(3)**.

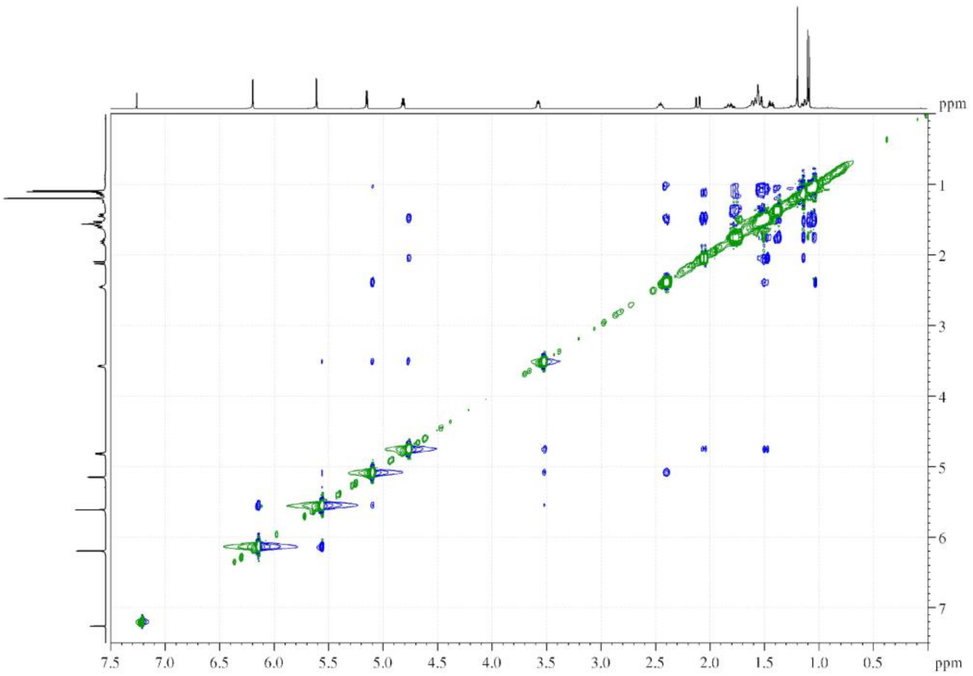

Figure S2.6: ^1H-^1H NOESY spectrum (500 MHz, $CDCl_3$) of alantolactone **(3)**.

Isoalantolactone ***(4)***

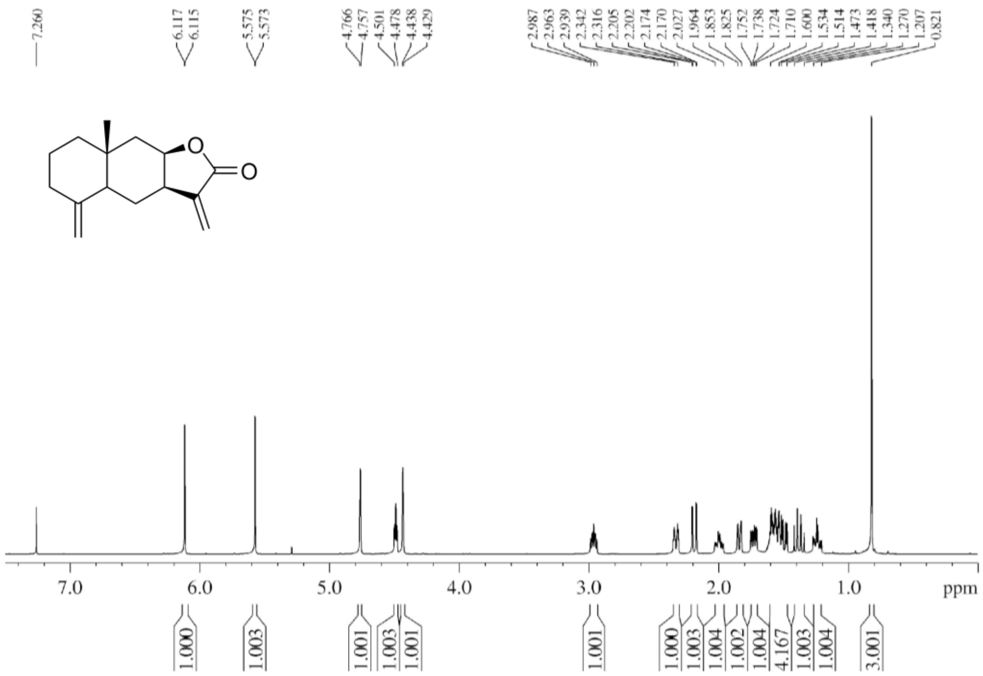

Figure S2.7: ^{1}H-NMR spectrum (500 MHz, $CDCl_3$) of isoalantolactone **(4)**.

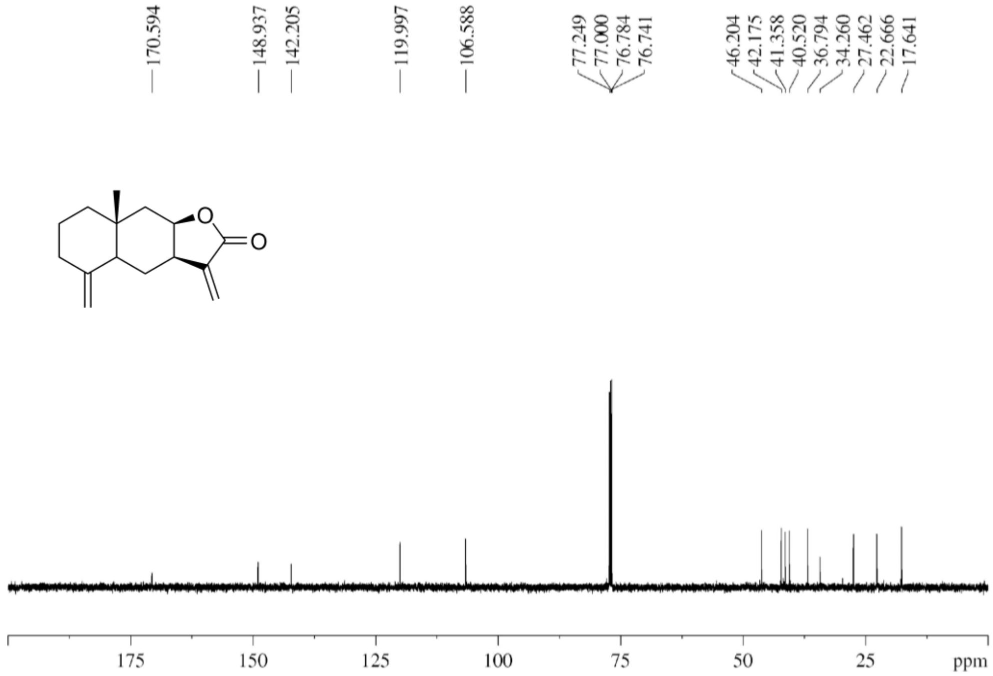

Figure S2.8: ^{13}C-NMR spectrum (500 MHz, $CDCl_3$) of isoalantolactone **(4)**.

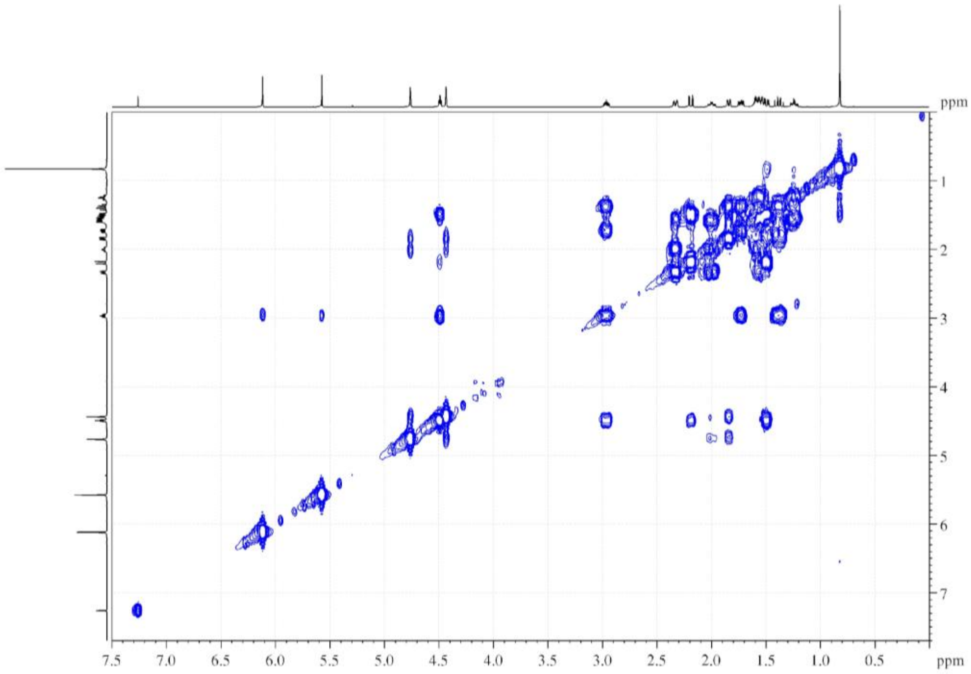

Figure S2.9: COSY spectrum (500 MHz, $CDCl_3$) of isoalantolactone **(4)**.

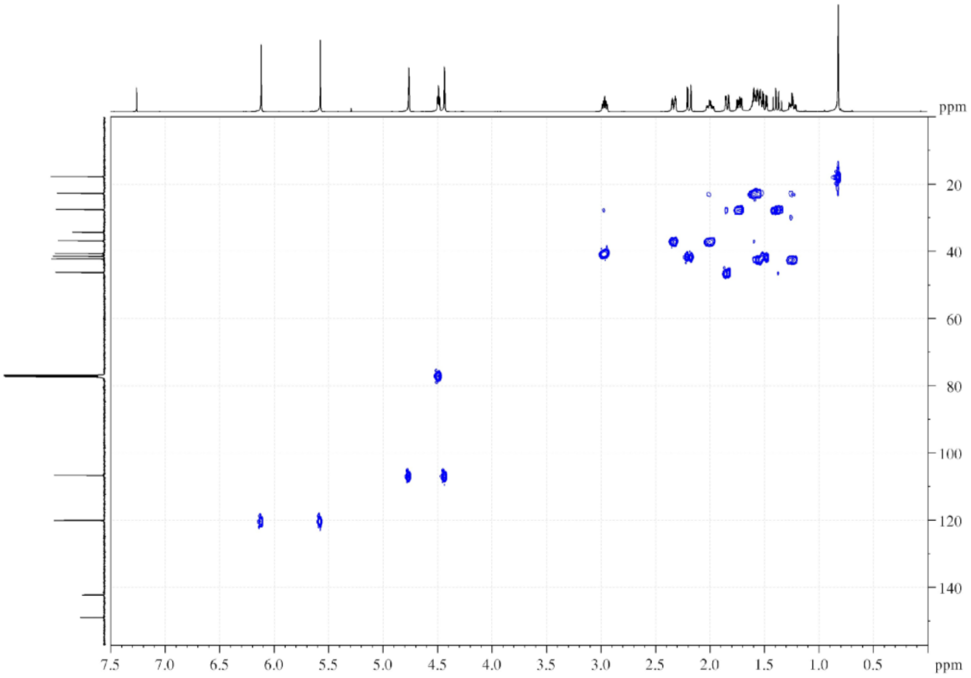

Figure S2.10: HSQC spectrum (500 MHz, $CDCl_3$) of isoalantolactone **(4)**.

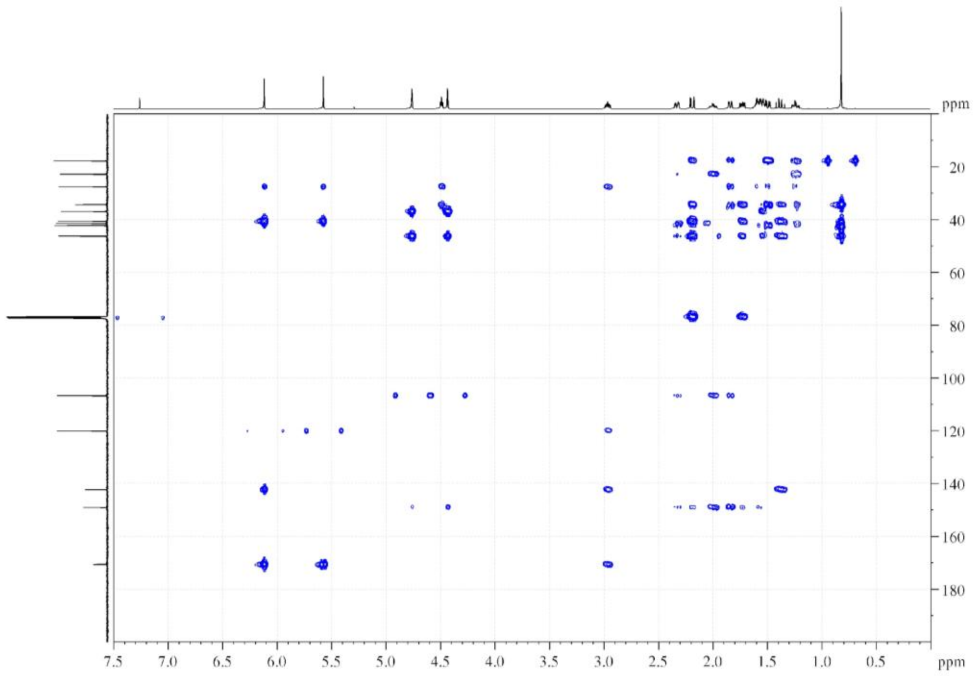

Figure S2.11: HMBC spectrum (500 MHz, $CDCl_3$) of isoalantolactone **(4)**.

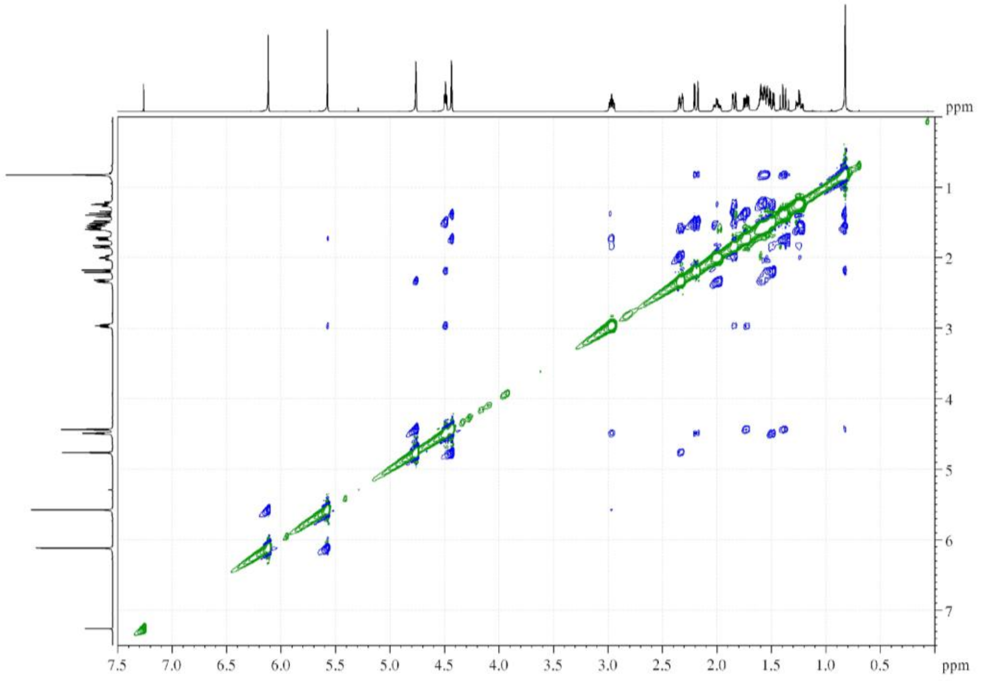

Figure S2.12: ^{1}H-^{1}H NOESY spectrum (500 MHz, $CDCl_3$) of isoalantolactone **(4)**.

2-(5,8a-dimethyl-2-oxo-2,3,3a,5,6,7,8,8a,9,9a-decahydronaphtho[2,3-b]furan- 3-yl) acetonitrile ***(5)***

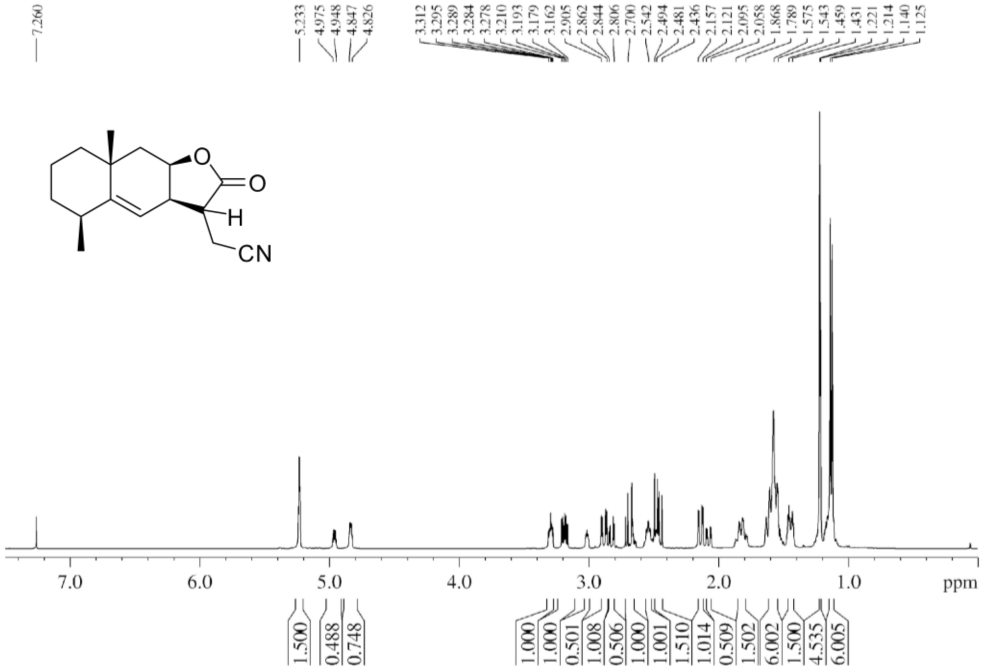

Figure S2.13: ^{1}H-NMR spectrum (500 MHz, $CDCl_3$) of **5a**, **5b** mixture.

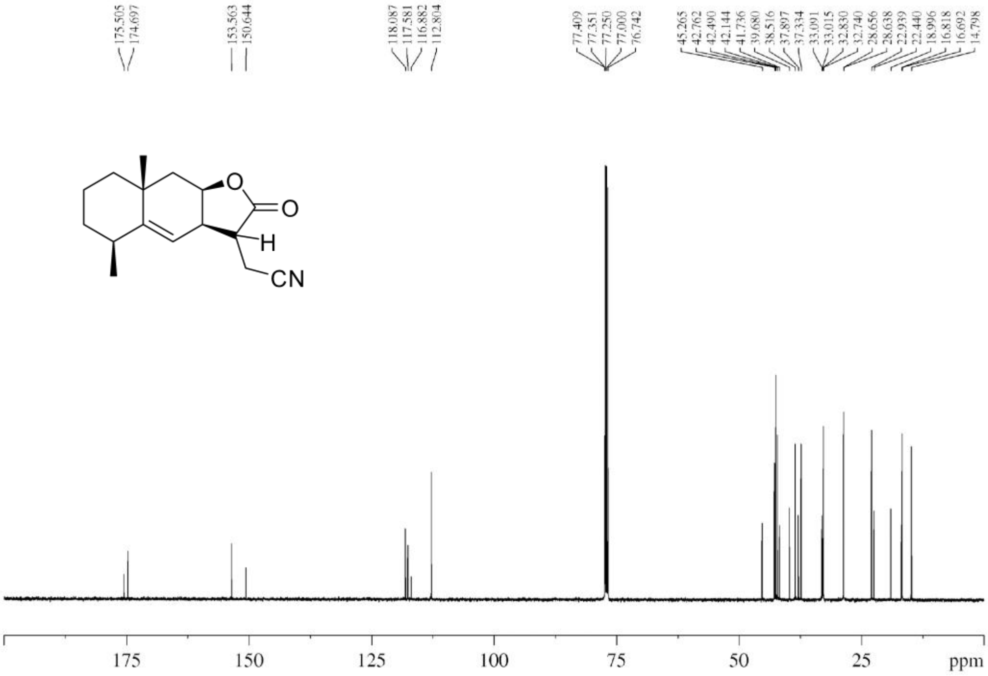

Figure S2.14: ^{13}C-NMR spectrum (500 MHz, $CDCl_3$) of **5a**, **5b** mixture.

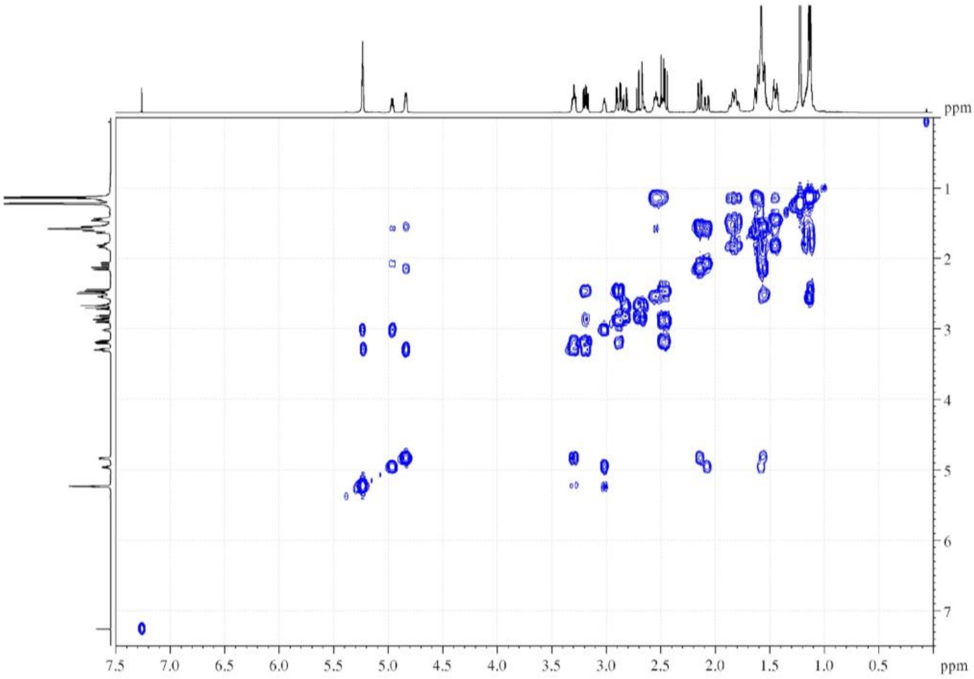

Figure S2.15: COSY spectrum (500 MHz, $CDCl_3$) of **5a**, **5b** mixture.

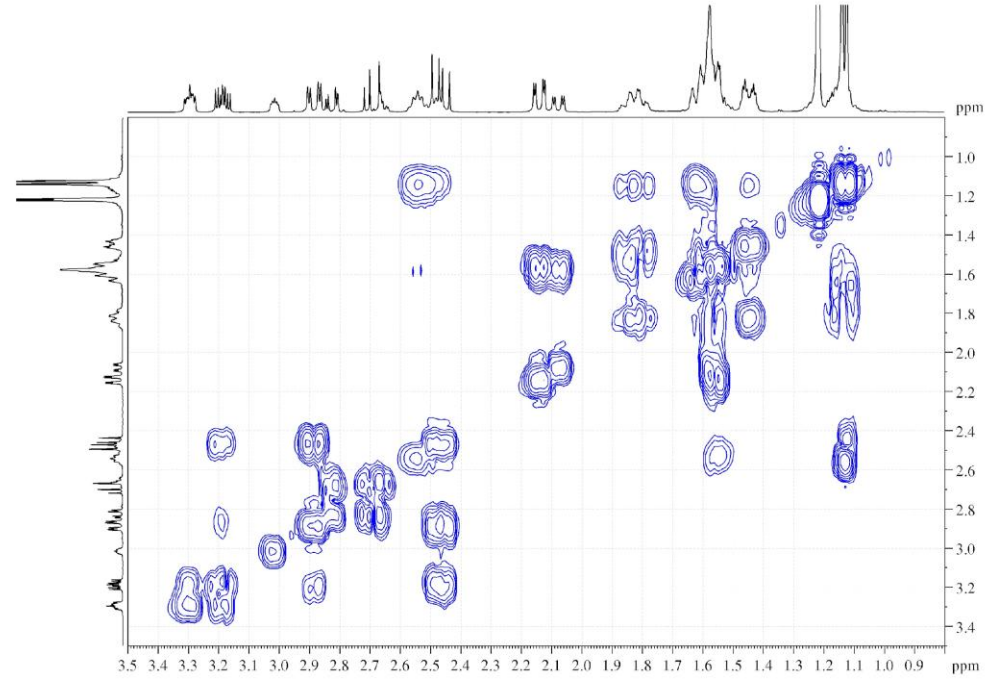

Figure S2.16: COSY spectrum (500 MHz, $CDCl_3$) of **5a**, **5b** mixture with focus on the lower ppm region.

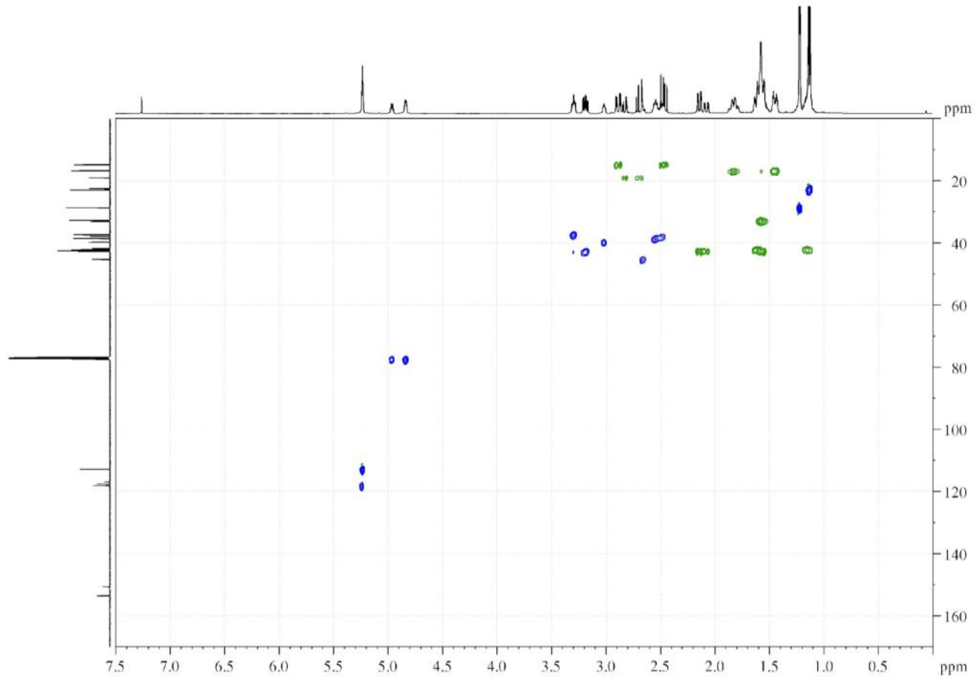

Figure S2.17: HSQC spectrum (500 MHz, $CDCl_3$) of **5a**, **5b** mixture.

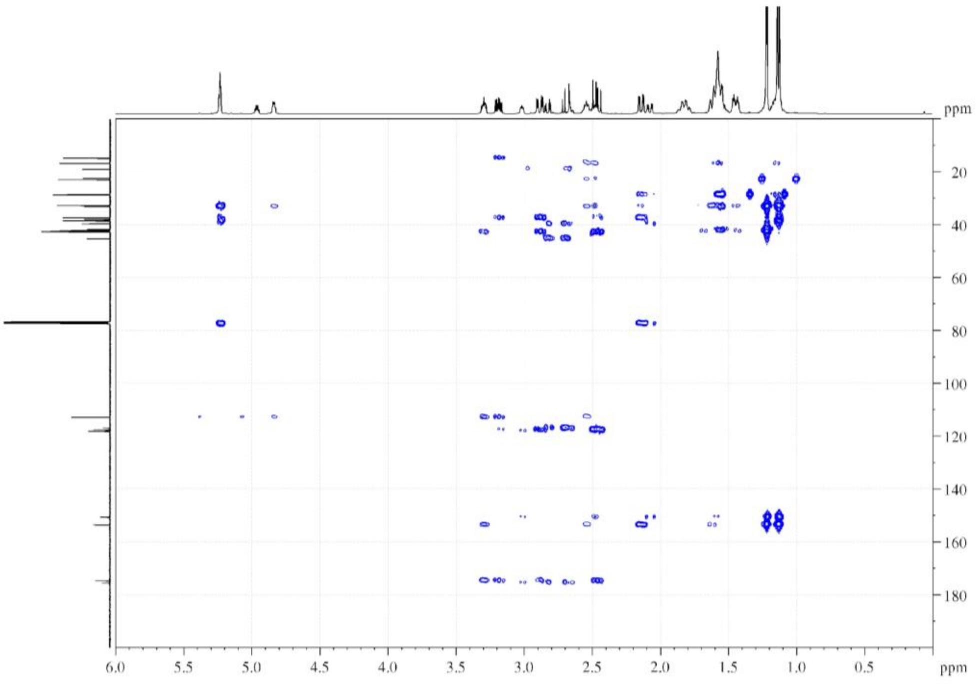

Figure S2.18: HMBC spectrum (500 MHz, $CDCl_3$) of **5a**, **5b** mixture.

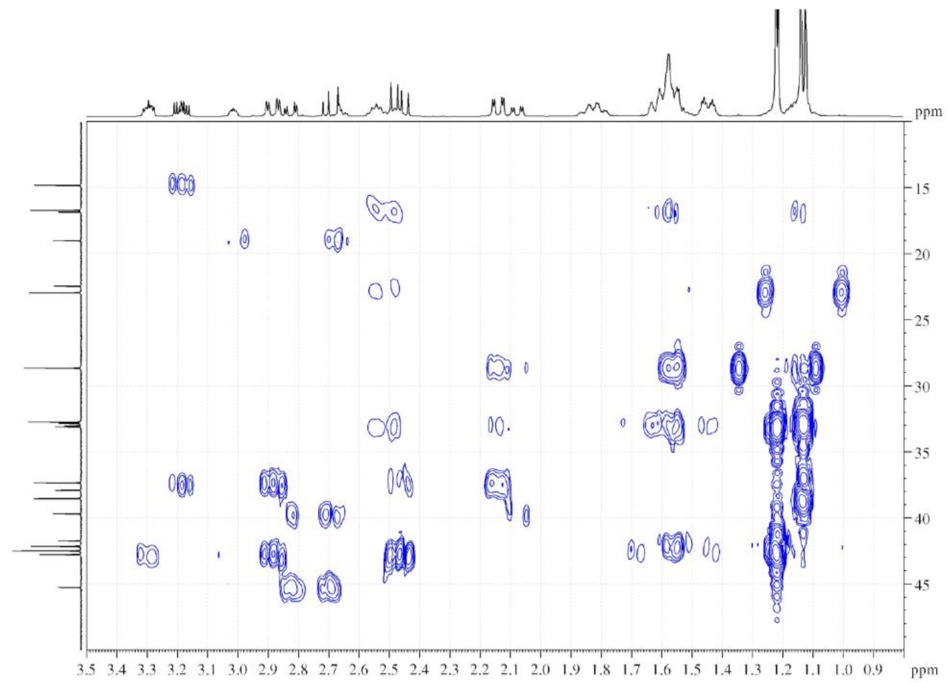

Figure S2.19: HMBC spectrum (500 MHz, $CDCl_3$) of **5a**, **5b** mixture with focus on the lower ppm region.

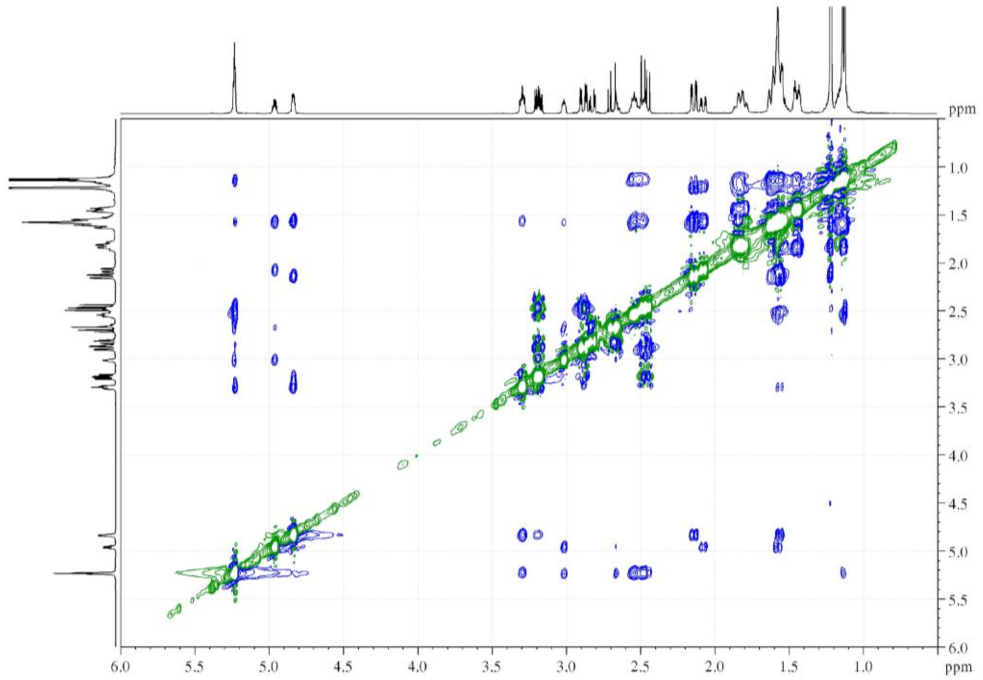

Figure SS2.20: ^{1}H-^{1}H NOESY spectrum (500 MHz, $CDCl_3$) of **5a**, **5b** mixture.

Synthesis of (2-(5,8a-dimethyl-2-oxo-2,3,3a,5,6,7,8,8a,9,9a-decahydronaphtho[2,3-b]furan)-3-yl)acetic acid ***(6)***

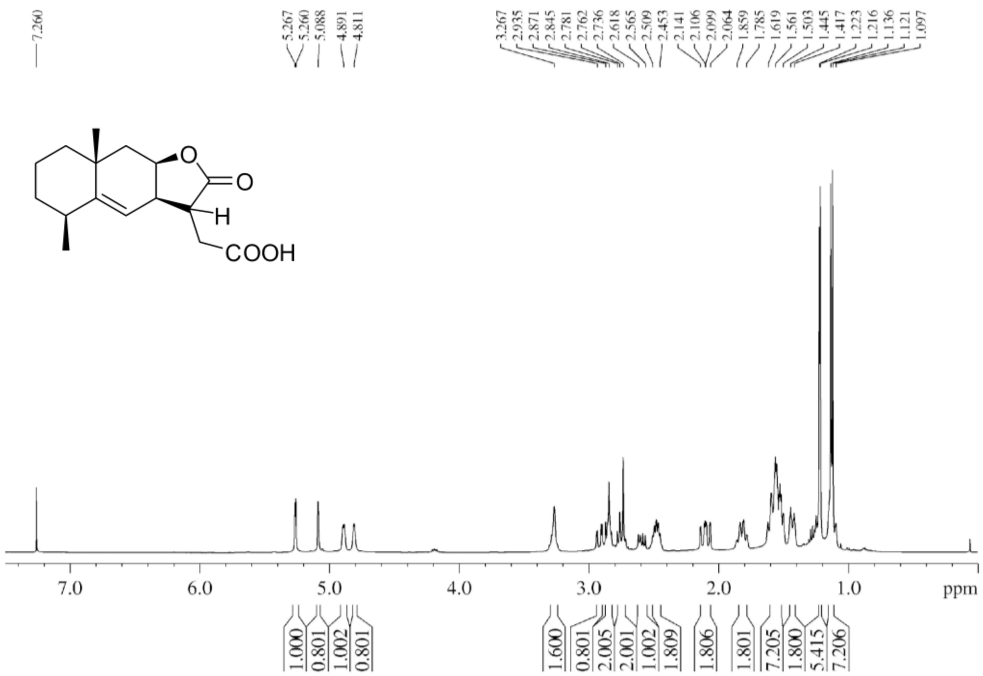

Figure S2.21: ^{1}H-NMR spectrum (500 MHz, $CDCl_3$) of **6a**, **6b** mixture.

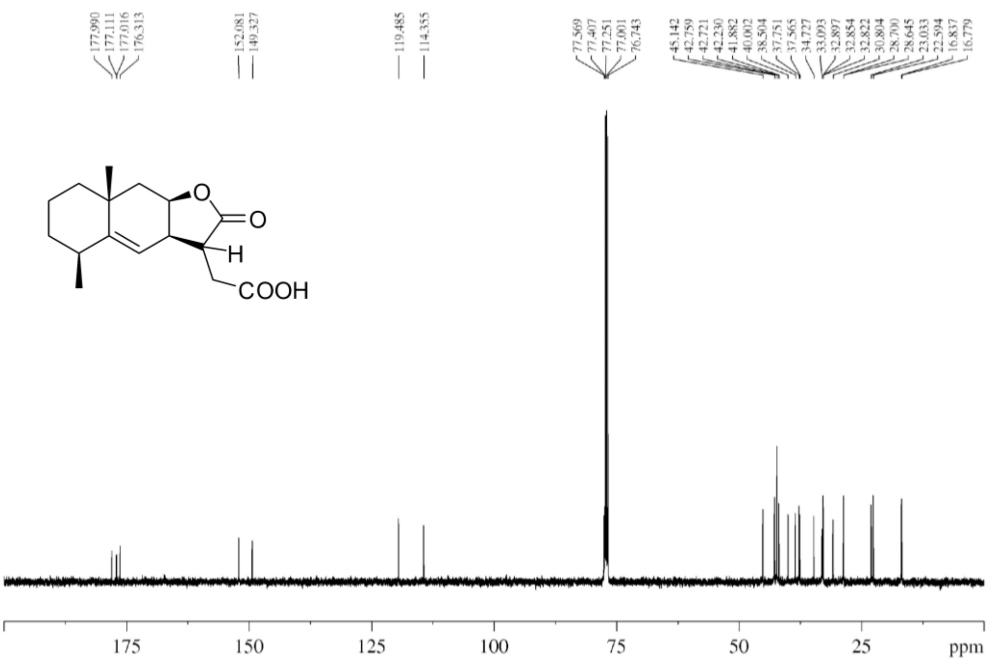

Figure S2.22: ^{13}C-NMR spectrum (500 MHz, $CDCl_3$) of **6a**, **6b** mixture.

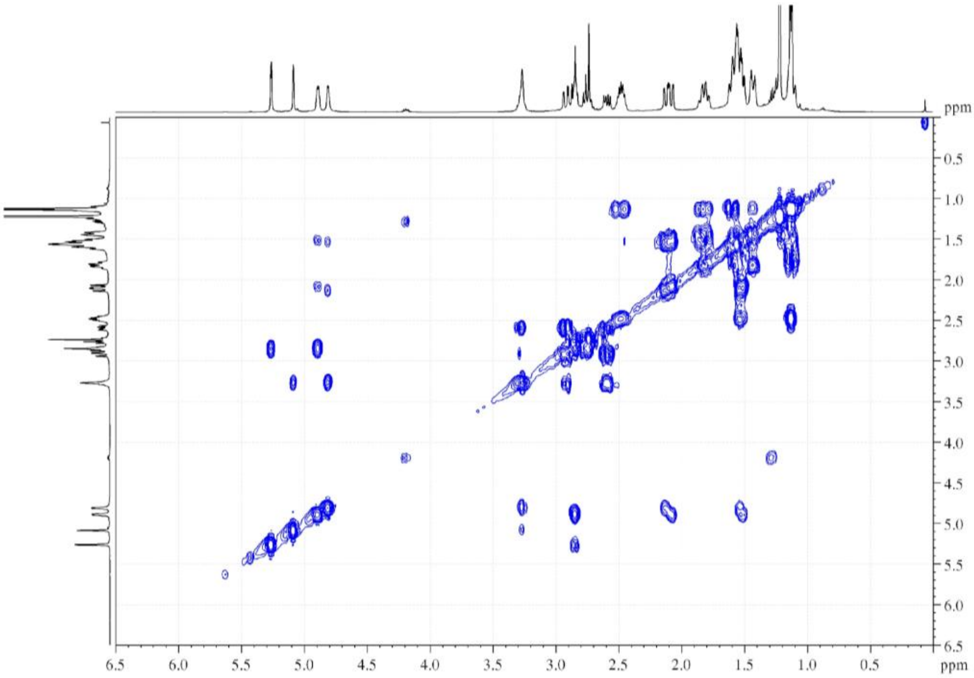

Figure S2.23: COSY spectrum (500 MHz, $CDCl_3$) of **6a**, **6b** mixture.

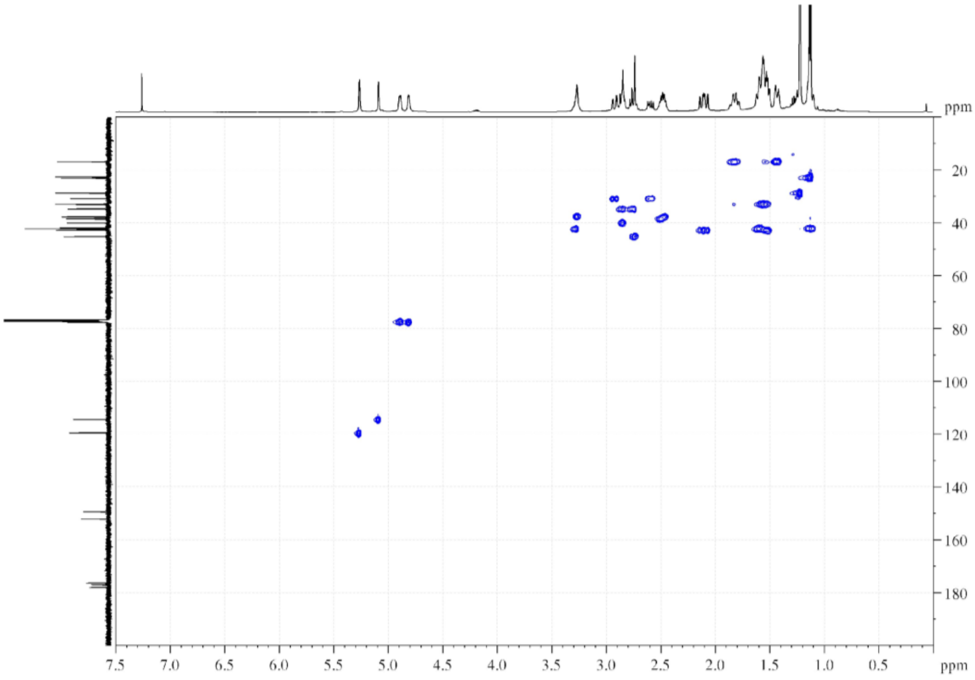

Figure S2.24: HSQC spectrum (500 MHz, $CDCl_3$) of **6a**, **6b** mixture.

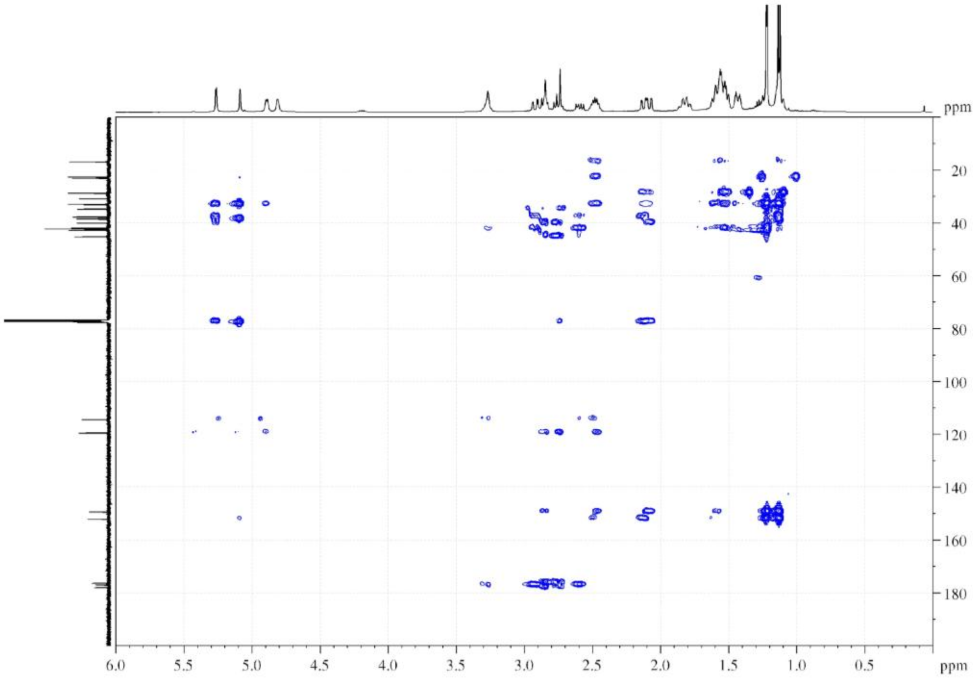

Figure S2.25: HMBC spectrum (500 MHz, $CDCl_3$) of **6a**, **6b** mixture.

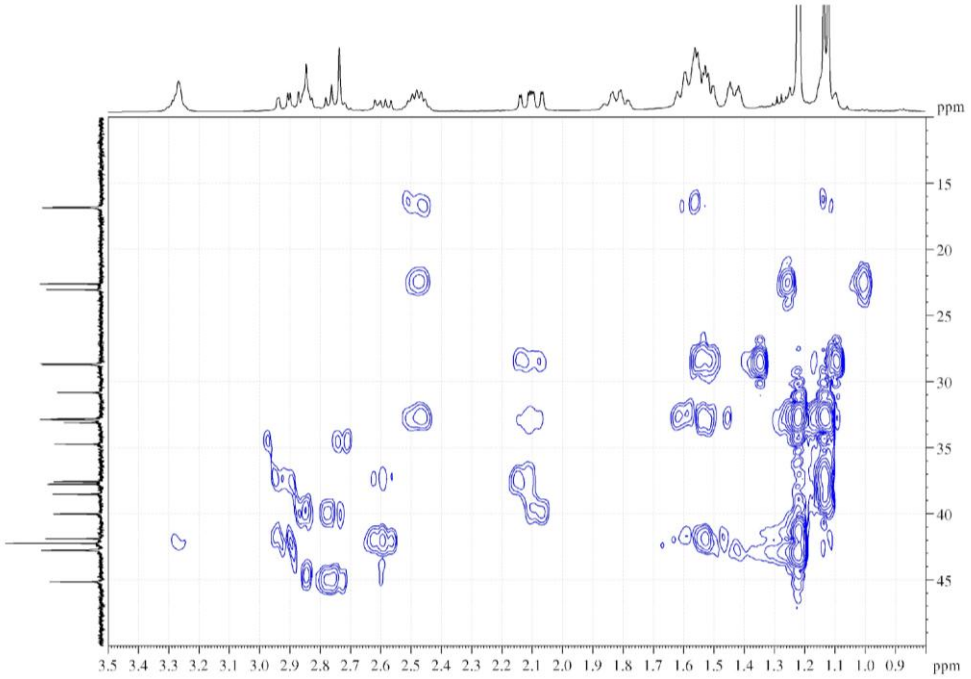

Figure S2.26: HMBC spectrum (500 MHz, $CDCl_3$) of **6a**, **6b** mixture with focus on the lower ppm region.

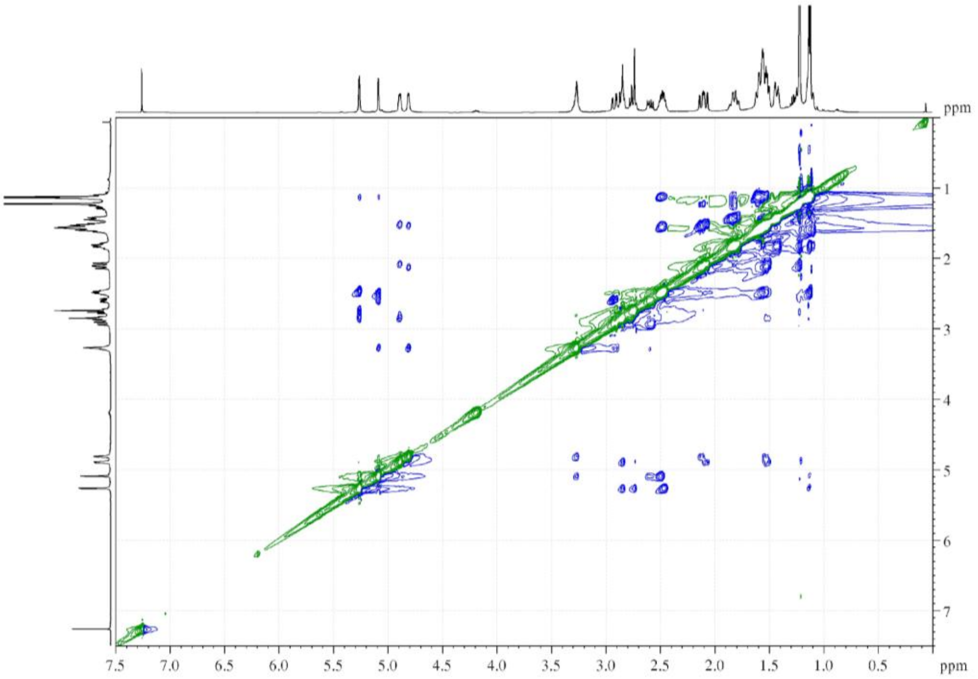

Figure S2.27: ^{1}H-^{1}H NOESY spectrum (500 MHz, $CDCl_3$) of **6a**, **6b** mixture.

*Synthesis of 2-(8a-methyl-5-methylene-2-oxododecahydronaphtho[2,3-b]furan-3-yl)acetonitrile **(7)***

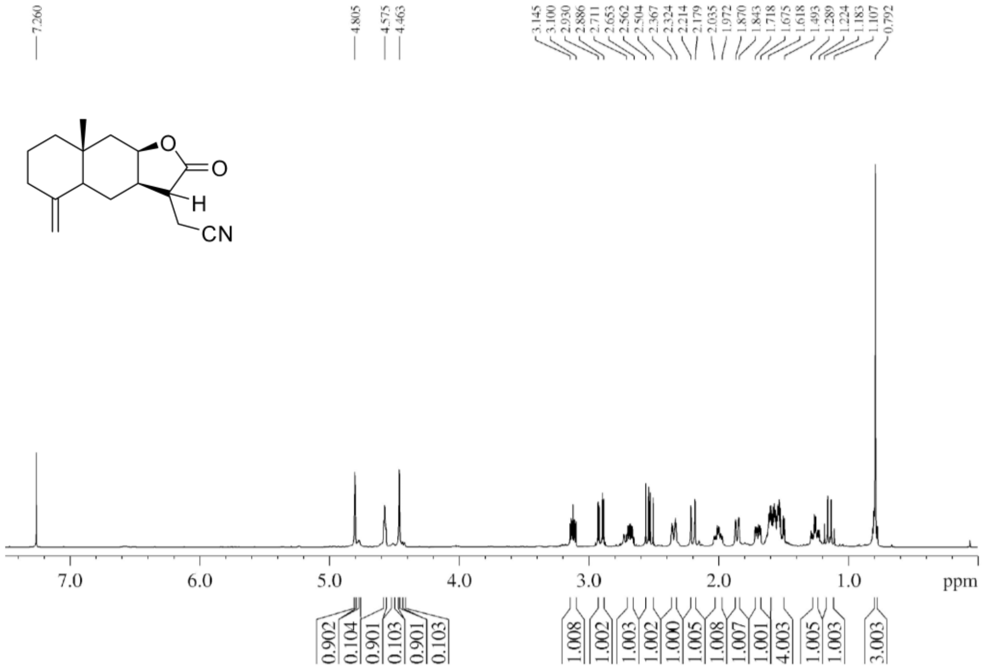

Figure S2.28: ^{1}H-NMR spectrum (500 MHz, $CDCl_3$) of **7a**, **7b** mixture.

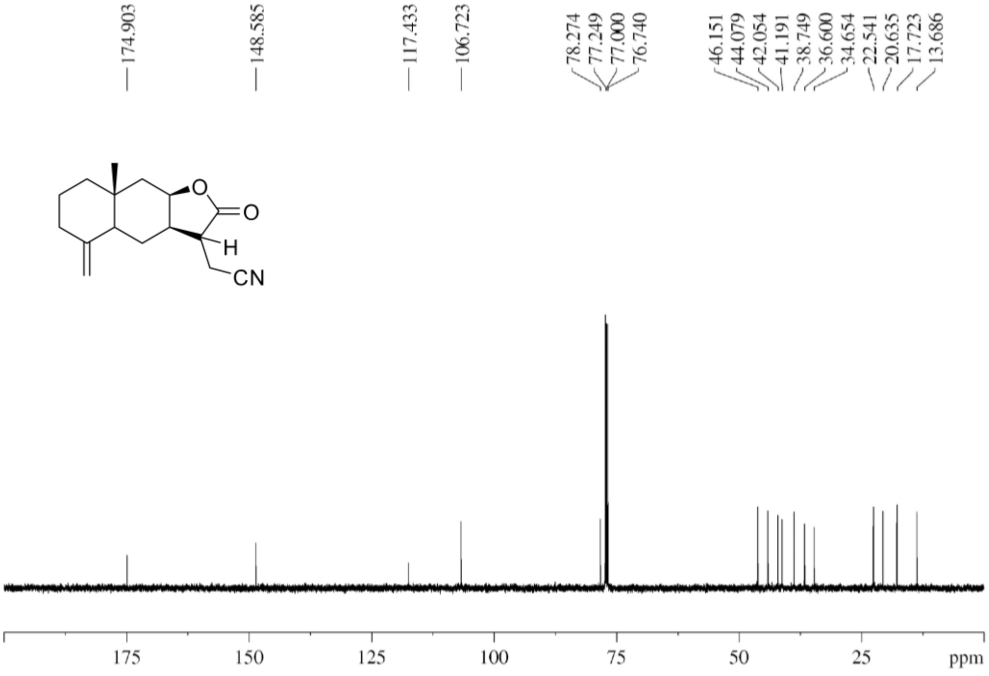

Figure S2.29: ^{13}C-NMR spectrum (500 MHz, $CDCl_3$) of **7a**, **7b** mixture.

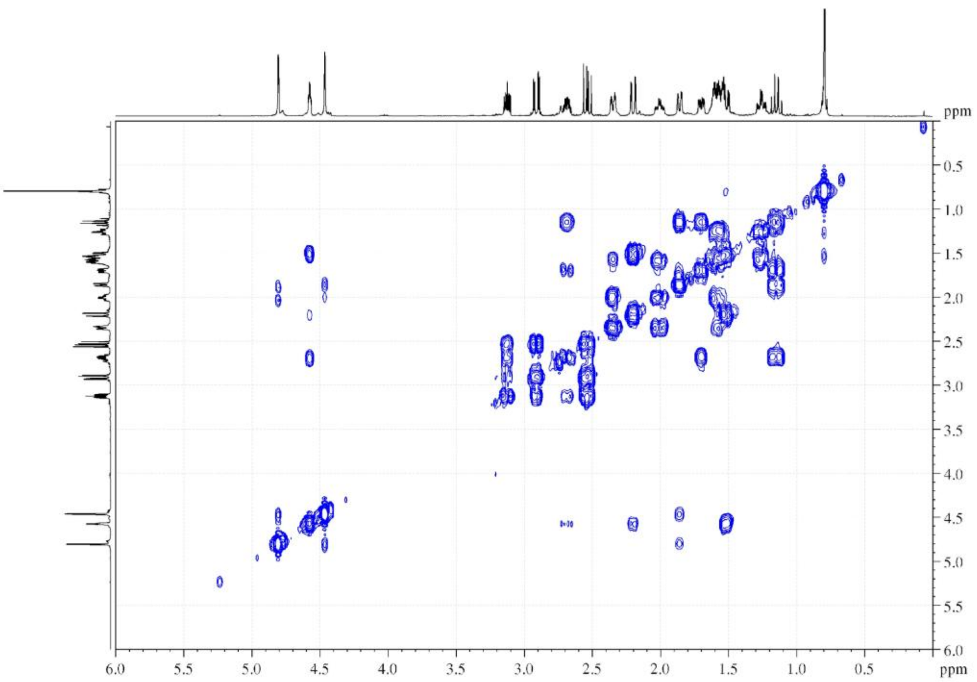

Figure S2.30: COSY spectrum (500 MHz, $CDCl_3$) of **7a**, **7b** mixture.

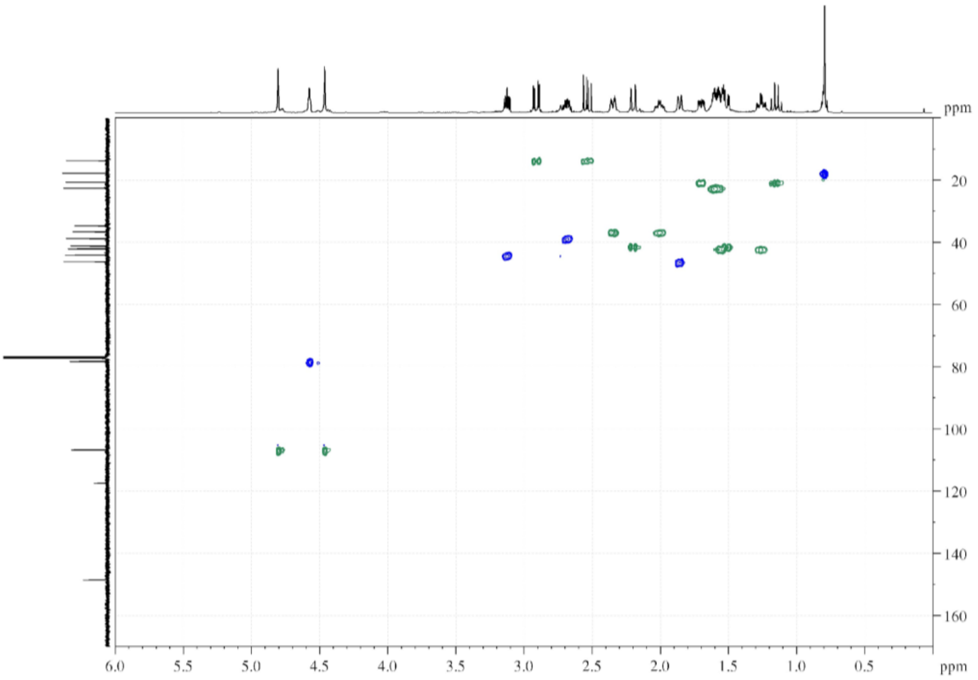

Figure S2.31: HSQC spectrum (500 MHz, $CDCl_3$) of **7a**, **7b** mixture.

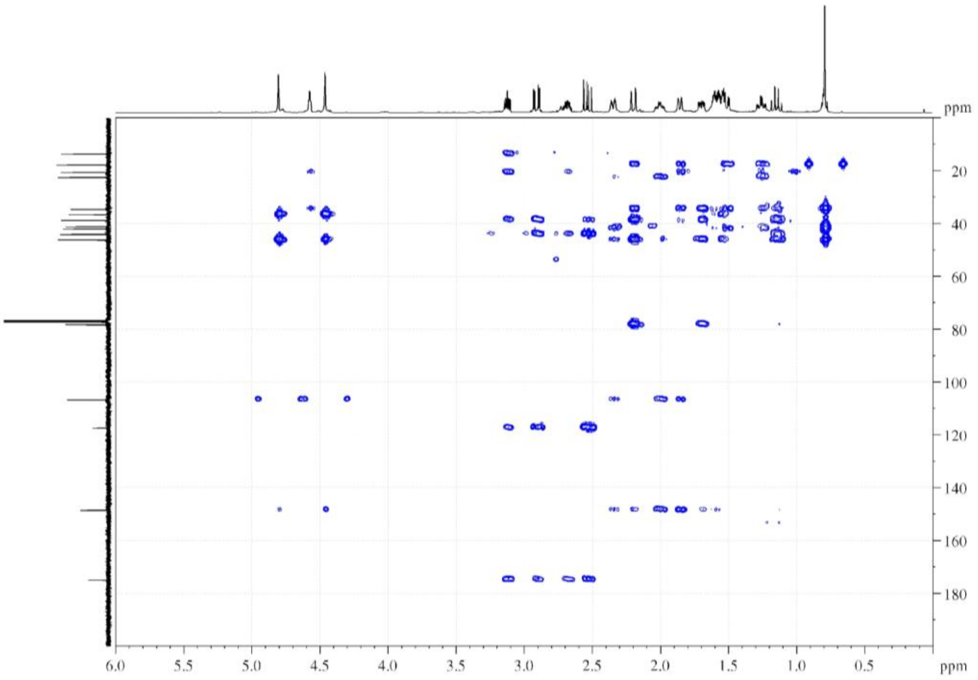

Figure SS2.32: HMBC spectrum (500 MHz, $CDCl_3$) of **7a**, **7b** mixture.

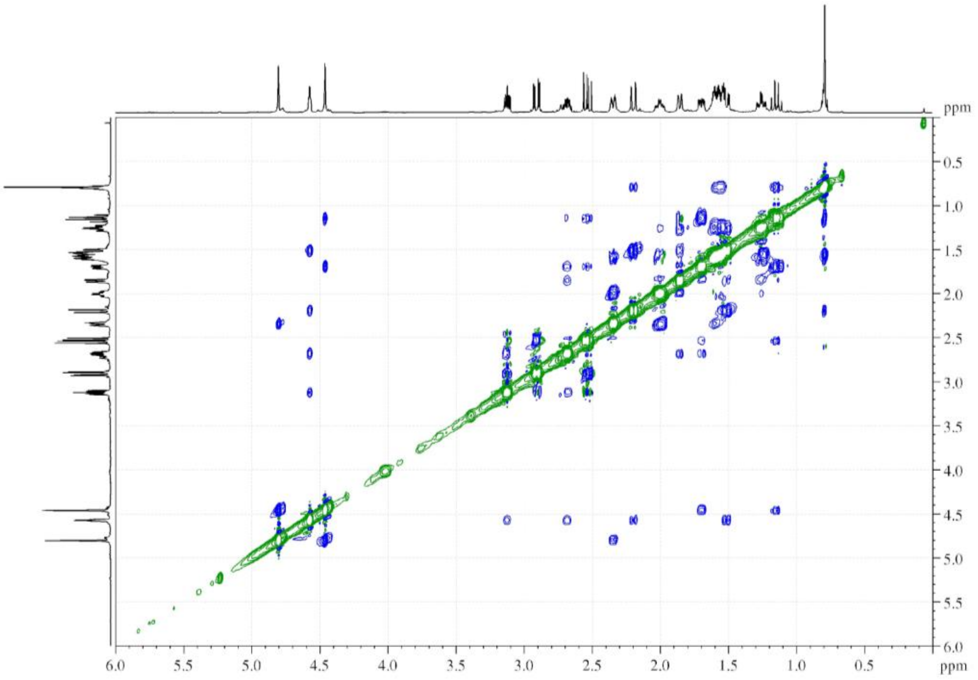

Figure S2.33: ^1H-^1H NOESY spectrum (500 MHz, $CDCl_3$) of **7a**, **7b** mixture.

Synthesis of 2-(8a-methyl-5-methylene-2-oxododecahydronaphtho[2,3-b]furan-3-yl)acetic acid ***(8)***

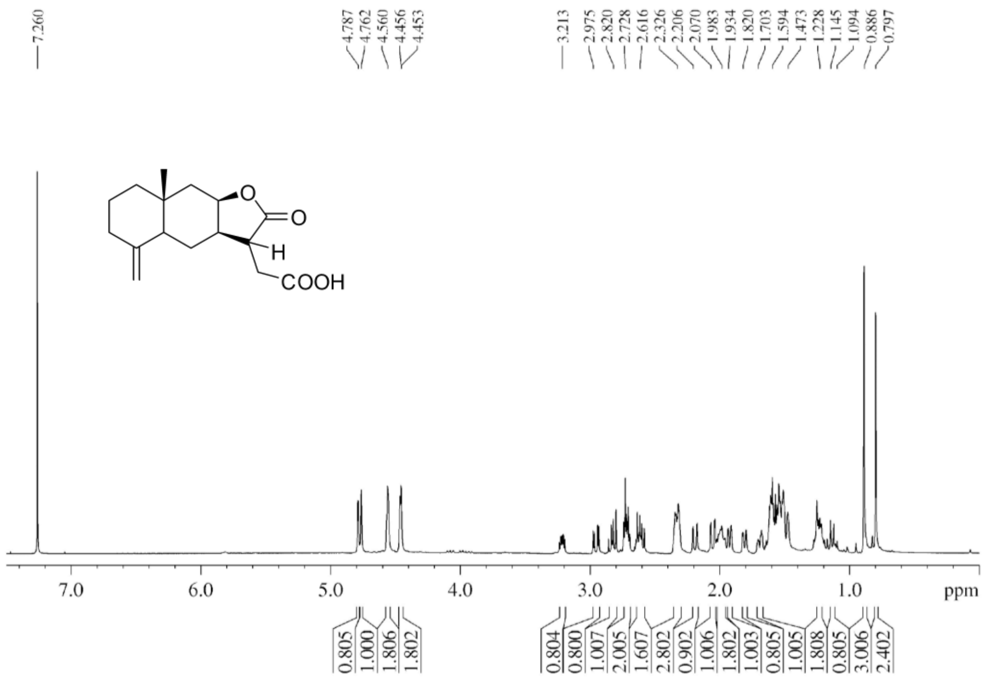

Figure S2.34: ^{1}H-NMR spectrum (500 MHz, $CDCl_3$) of **8a**, **8b** mixture.

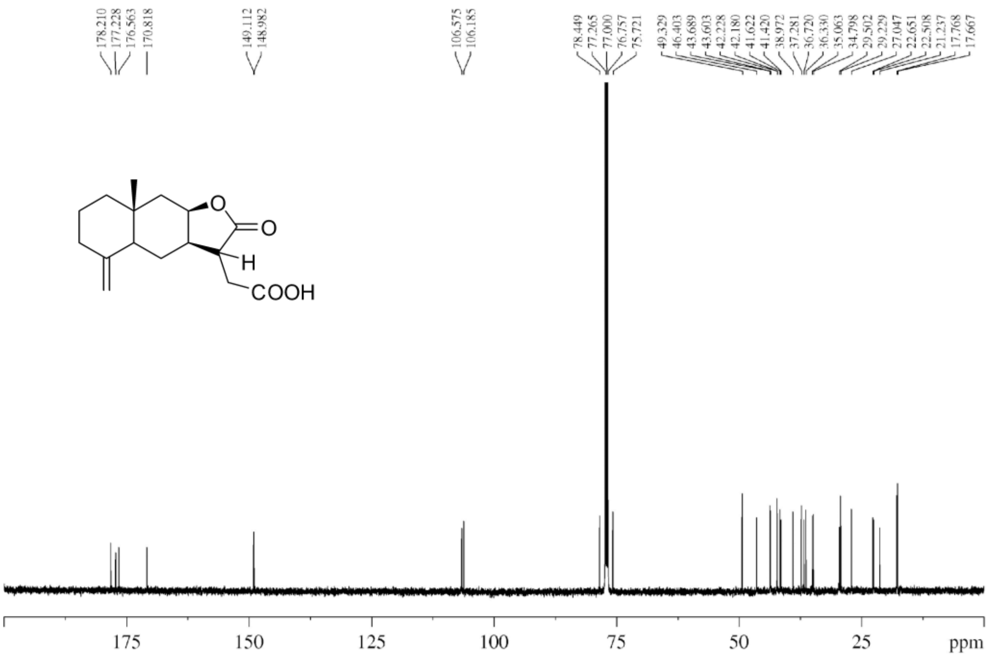

Figure S2.35: ^{13}C-NMR spectrum (500 MHz, $CDCl_3$) of **8a**, **8b** mixture.

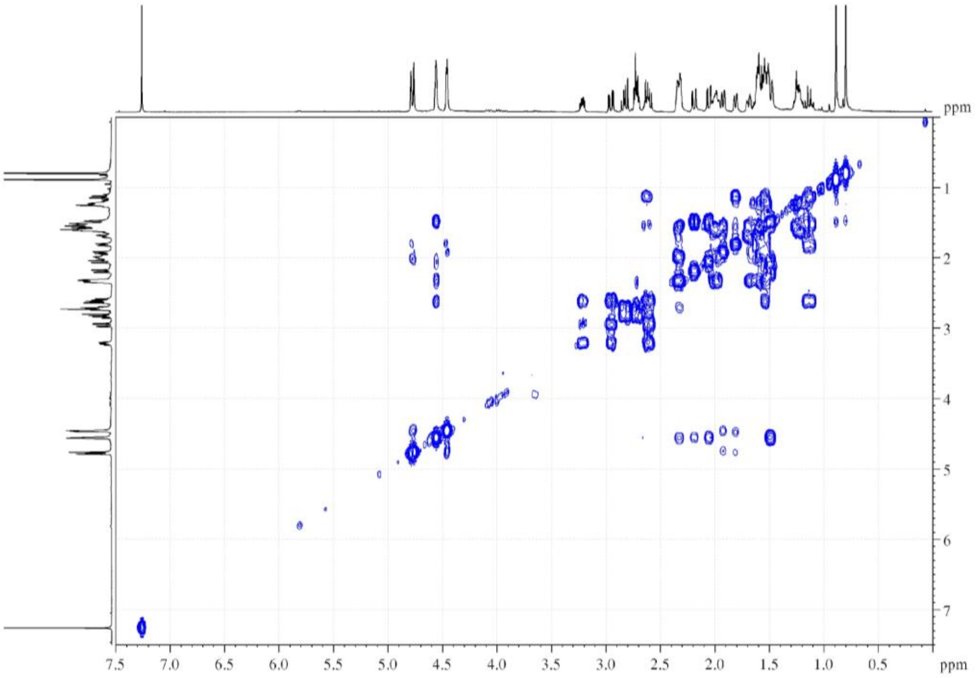

Figure S2.36: COSY spectrum (500 MHz, $CDCl_3$) of **8a**, **8b** mixture.

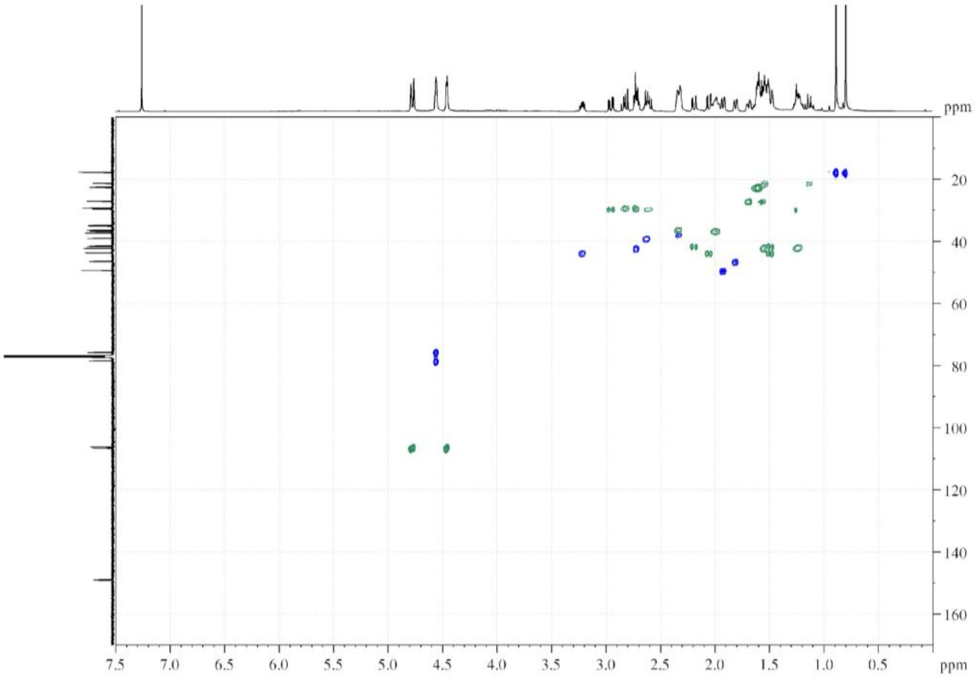

Figure S2.37: HSQC spectrum (500 MHz, $CDCl_3$) of **8a**, **8b** mixture.

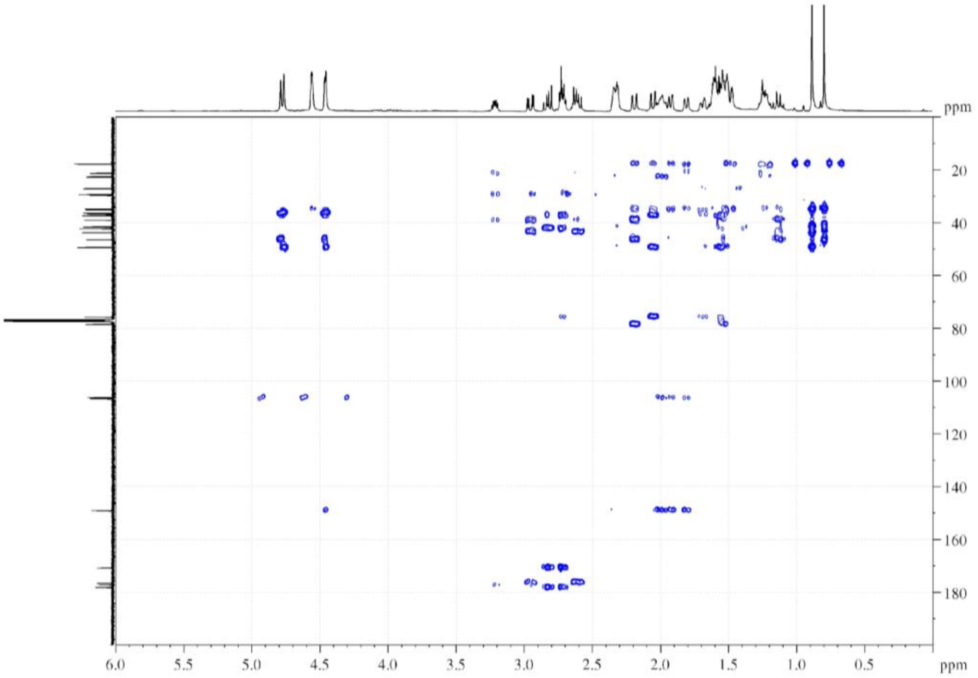

Figure S2.38: HMBC spectrum (500 MHz, $CDCl_3$) of **8a**, **8b** mixture.

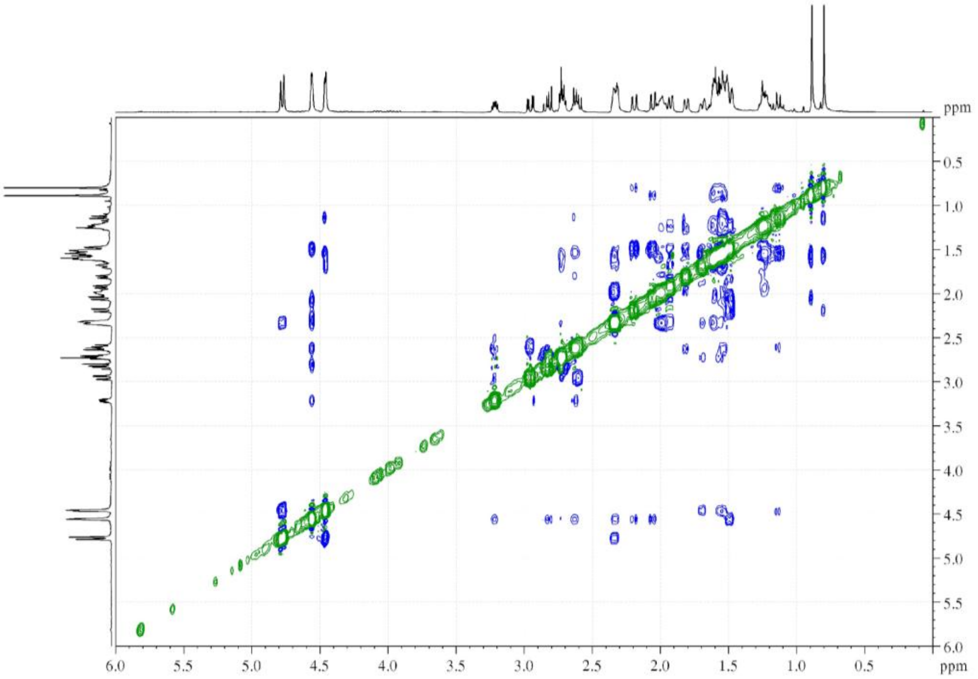

Figure S2.39: ^1H-^1H NOESY spectrum (500 MHz, $CDCl_3$) of **8a**, **8b** mixture.

Synthesis of 3-(azidomethyl)-5,8a-dimethyl-3a,5,6,7,8,8a,9,9a-octahydronaphtho[2,3-b]furan-2(3H) -one ***(9)***

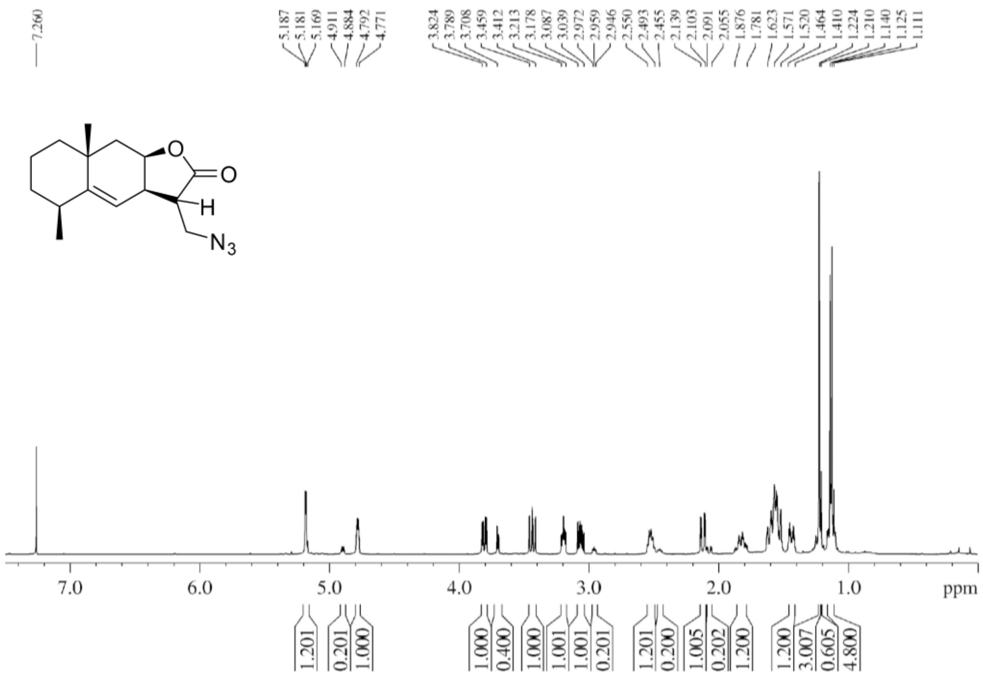

Figure S2.40: ^{1}H-NMR spectrum (500 MHz, $CDCl_3$) of **9a**, **9b** mixture.

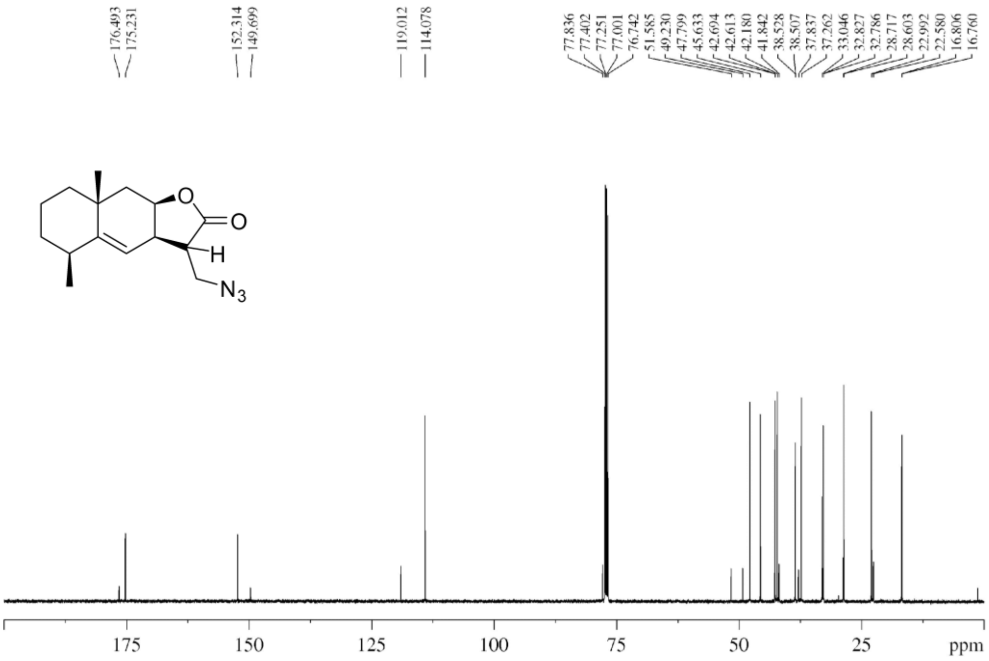

Figure S2.41: ^{13}C-NMR spectrum (500 MHz, $CDCl_3$) of **9a**, **9b** mixture.

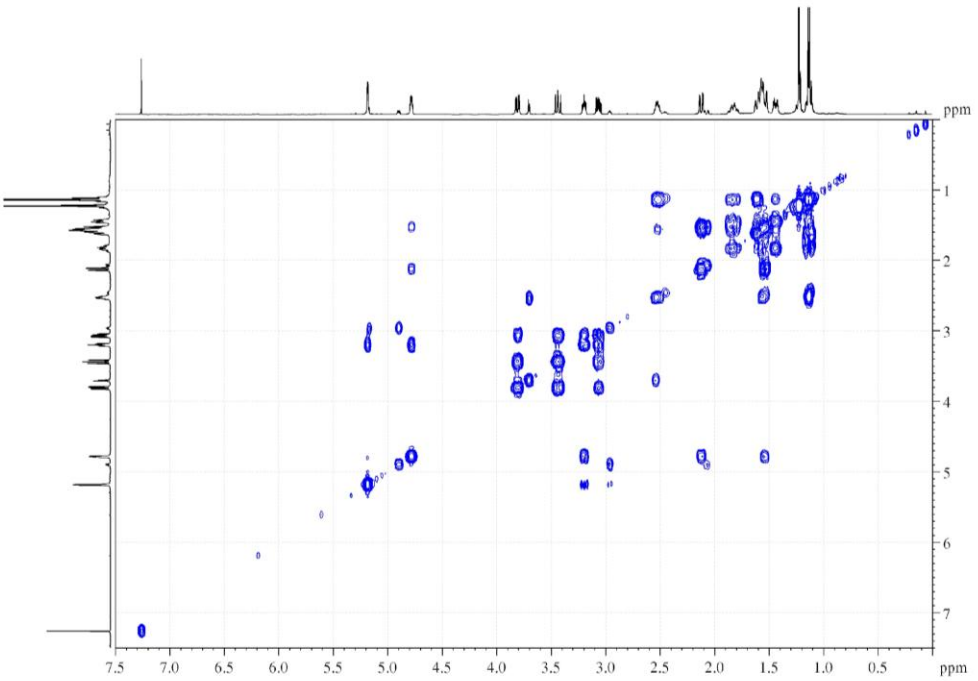

Figure S2.42: COSY spectrum (500 MHz, $CDCl_3$) of **9a**, **9b** mixture.

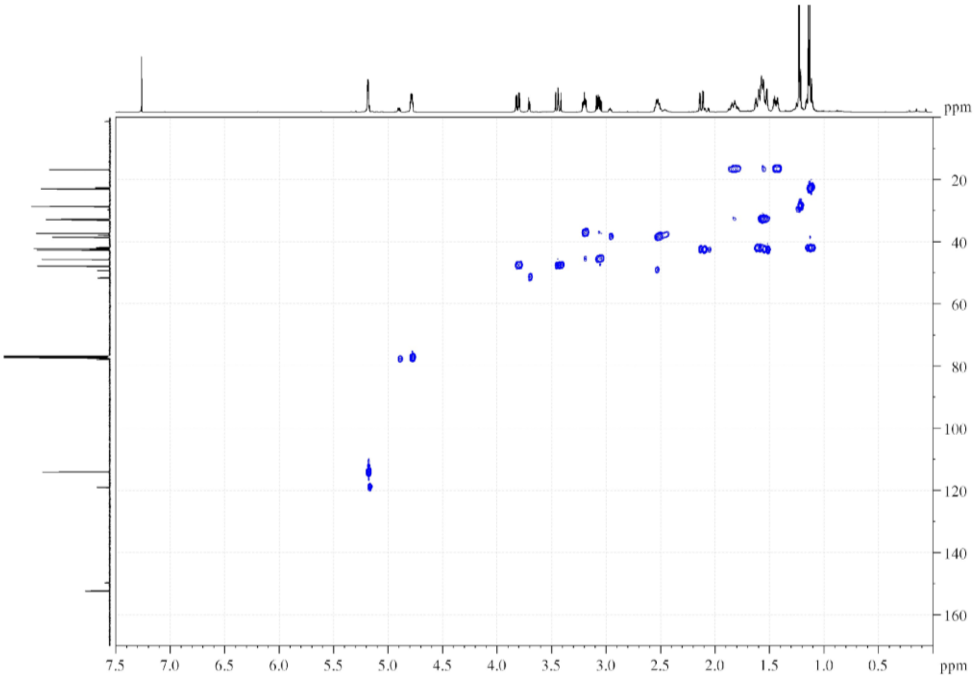

Figure S2.43: HSQC spectrum (500 MHz, $CDCl_3$) of **9a**, **9b** mixture.

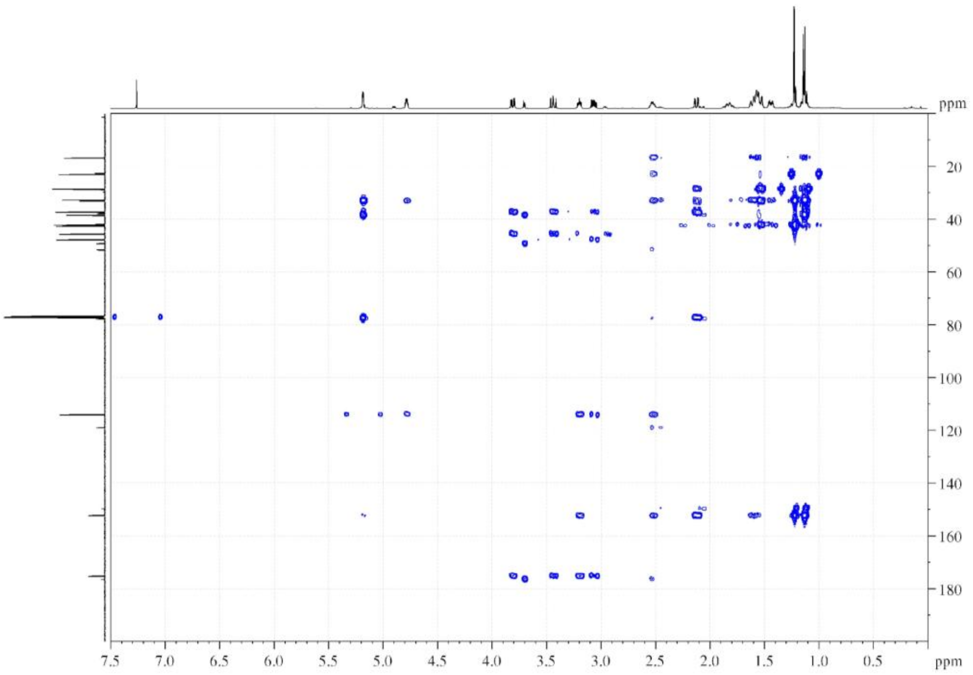

Figure S2.44: HMBC spectrum (500 MHz, $CDCl_3$) of **9a**, **9b** mixture.

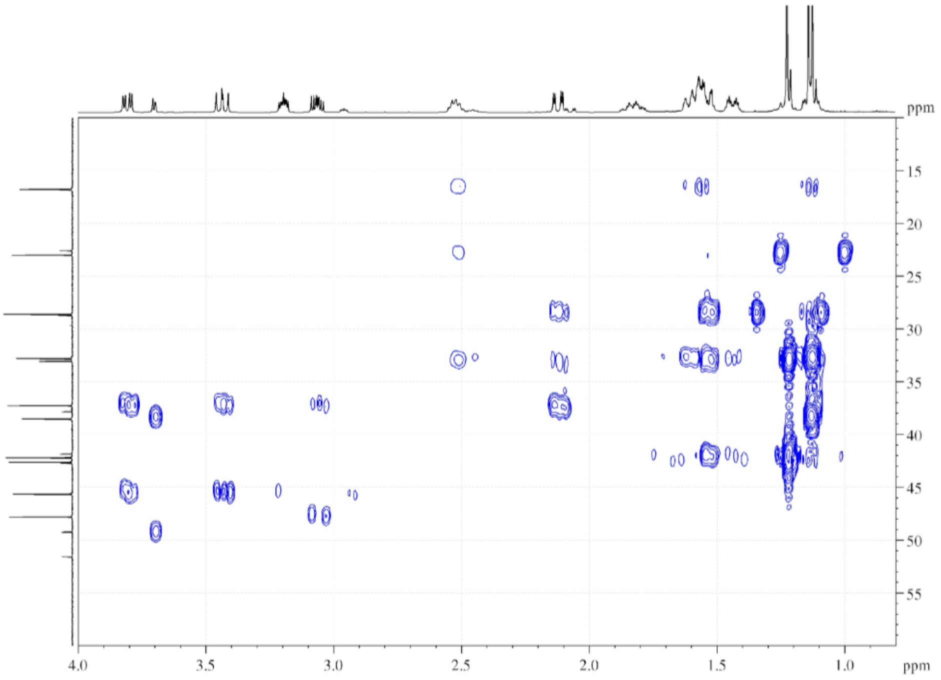

Figure S2.45: HMBC spectrum (500 MHz, $CDCl_3$) of **9a**, **9b** mixture with focus on the low ppm region.

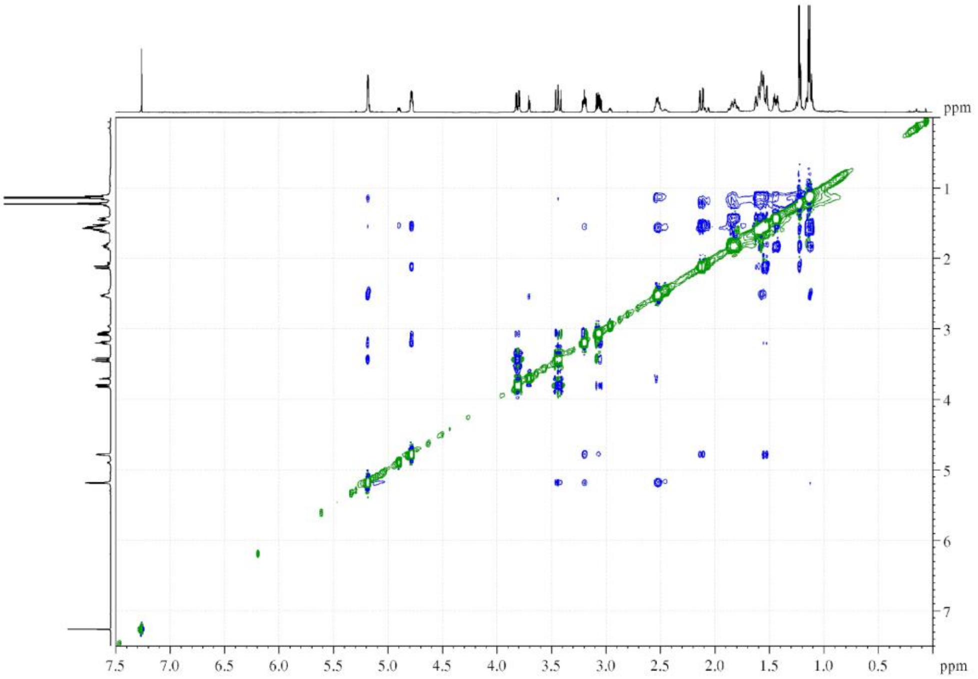

Figure S2.46: ^{1}H-^{1}H NOESY spectrum (500 MHz, $CDCl_3$) of **9a**, **9b** mixture.

Synthesis of (3R)-3-(aminomethyl)-5,8a-dimethyl-3a,5,6,7,8,8a,9,9a-octahydronaphtho[2,3-b]furan-2(3H)-one ***(10)***

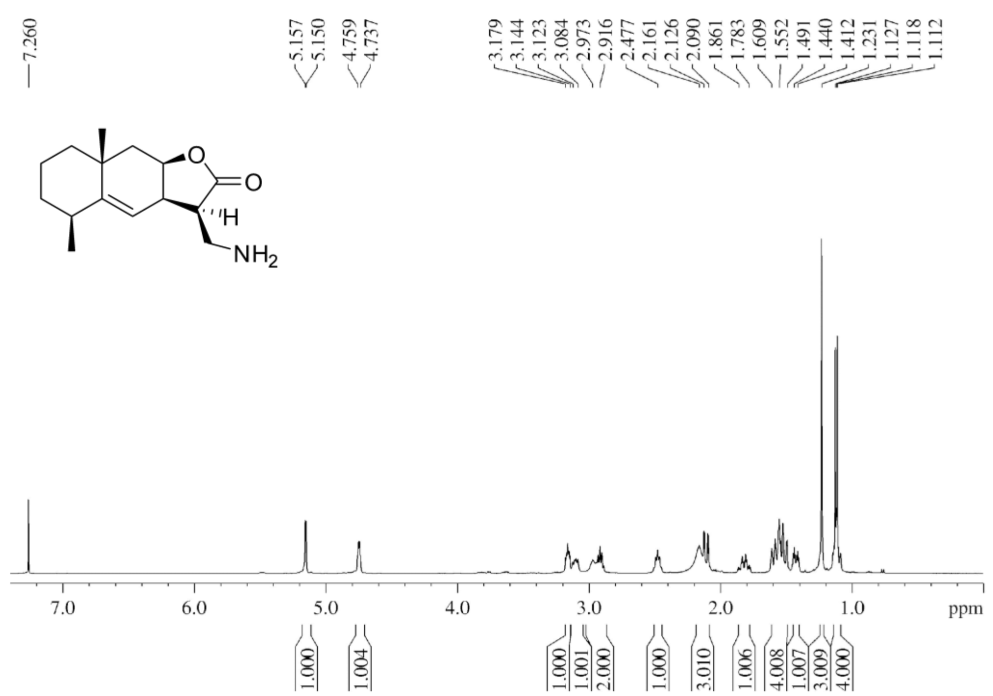

Figure S2.47: ^{1}H-NMR spectrum (500 MHz, $CDCl_3$) of diastereoisomer **10a**.

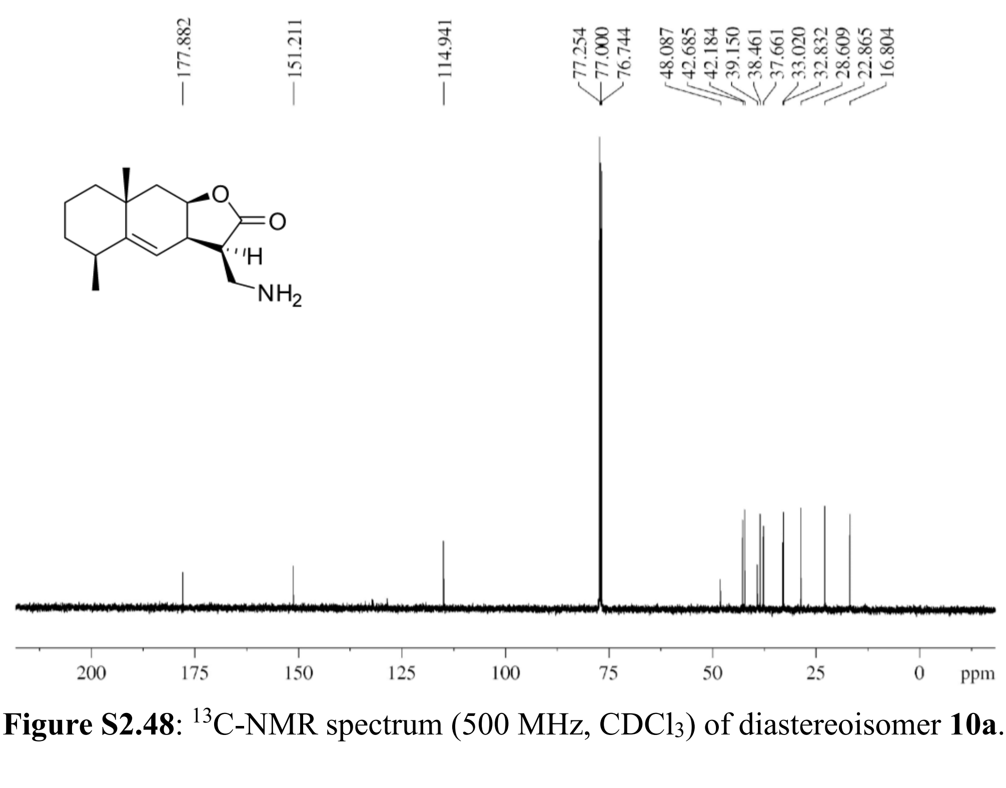

Figure S2.48: ^{13}C-NMR spectrum (500 MHz, $CDCl_3$) of diastereoisomer **10a**.

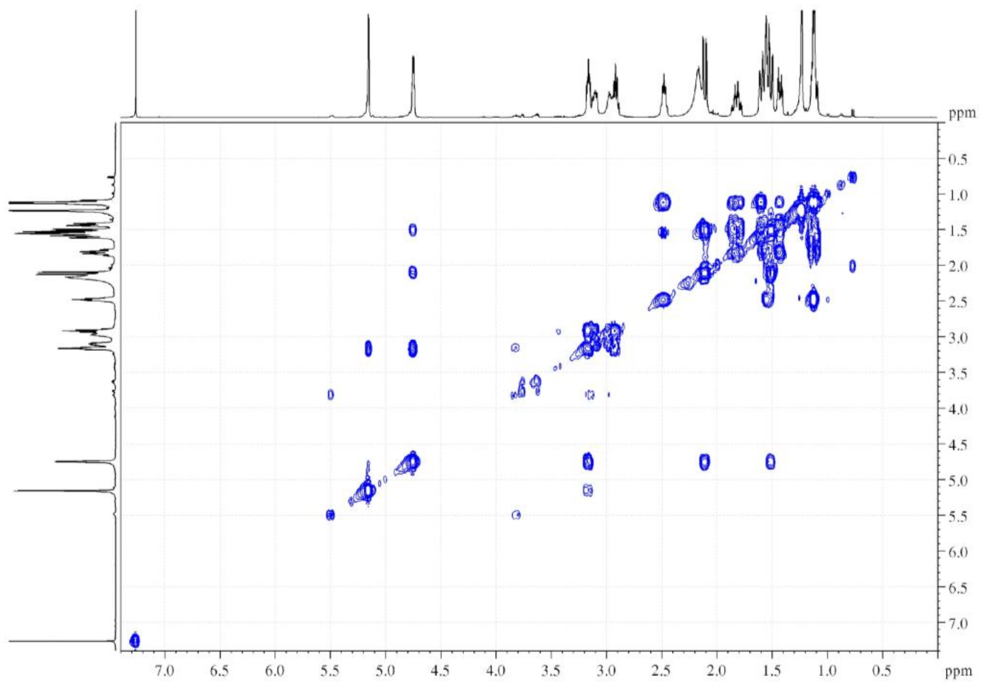

Figure S2.49: COSY spectrum (500 MHz, $CDCl_3$) of diastereoisomer **10a**.

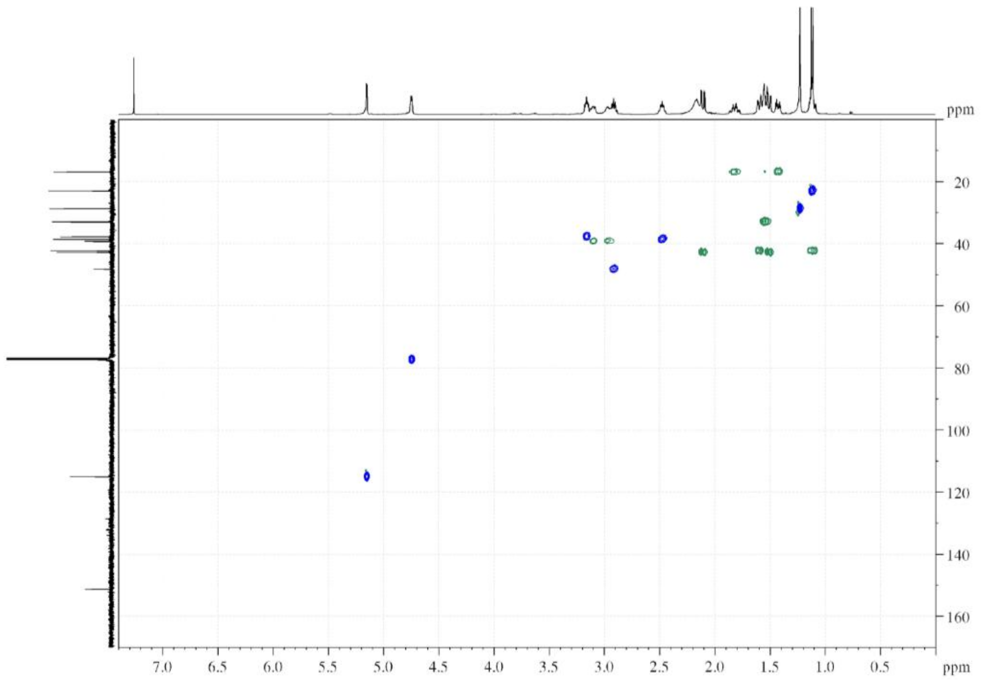

Figure S2.50: HSQC spectrum (500 MHz, $CDCl_3$) of diastereoisomer **10a**.

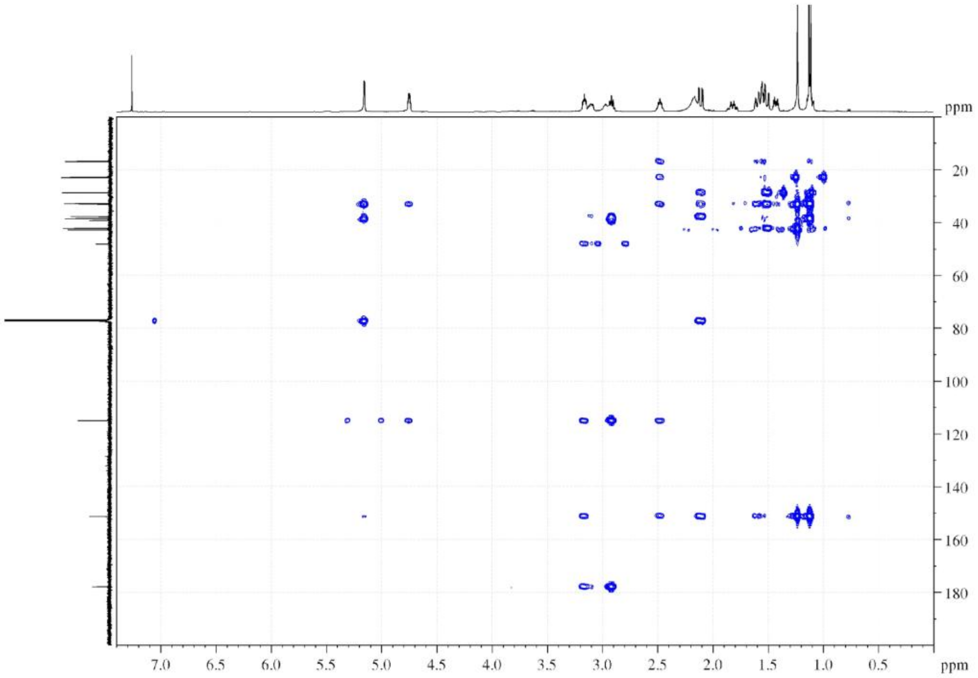

Figure S2.51: HMBC spectrum (500 MHz, $CDCl_3$) of diastereoisomer **10a**.

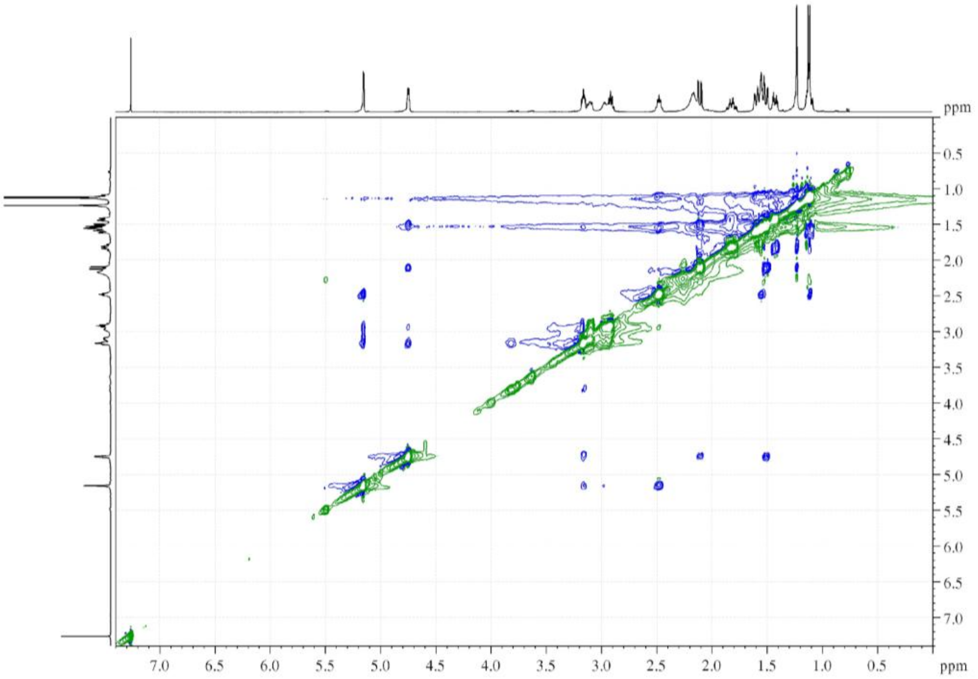

Figure S2.52: ^{1}H-^{1}H NOESY spectrum (500 MHz, $CDCl_3$) of diastereoisomer **10a**.

*Synthesis of (3R)-3-(azidomethyl)-8a-methyl-5-methylene-decahydronaphtho[2,3-b]furan-2(3H)-one **(11)***

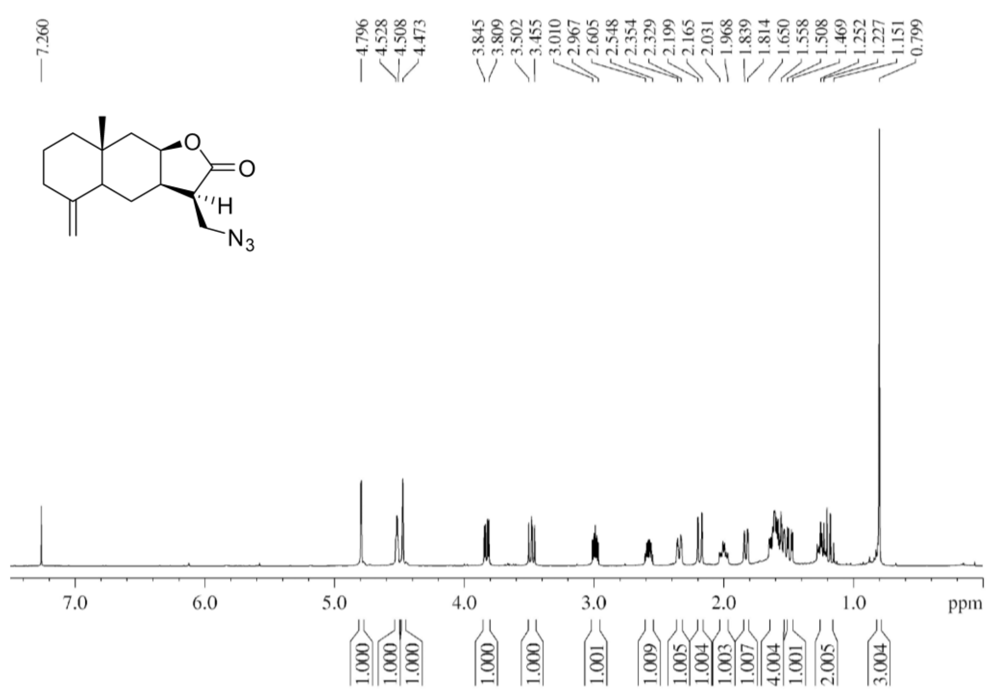

Figure S2.53: ^{1}H-NMR spectrum (500 MHz, $CDCl_3$) of diastereoisomer **11a**.

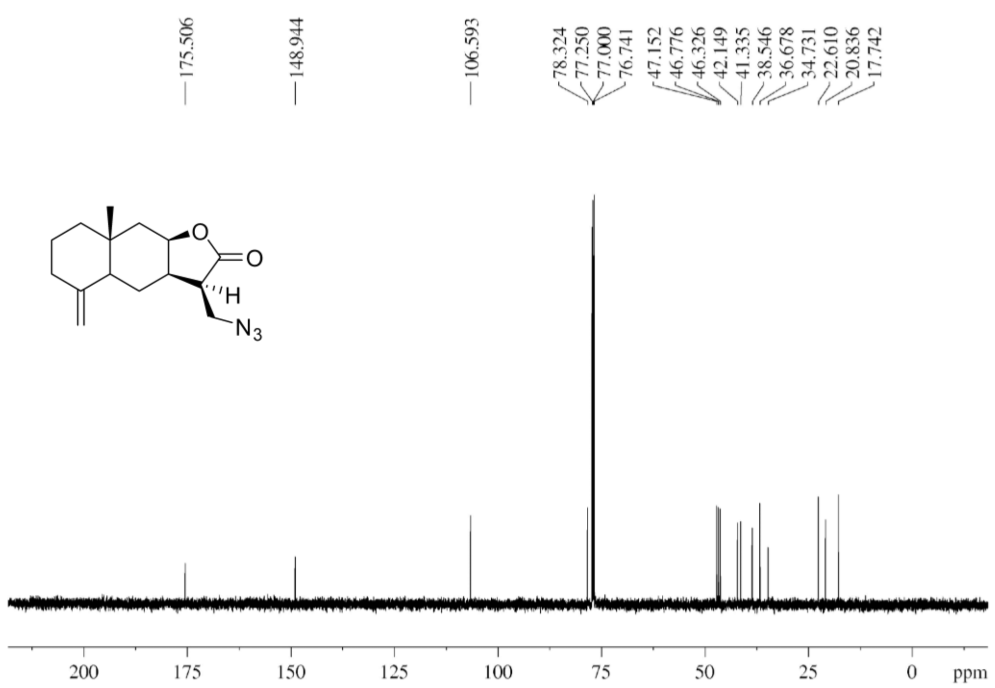

Figure S2.54: ^{13}C-NMR spectrum (500 MHz, $CDCl_3$) of diastereoisomer **11a**.

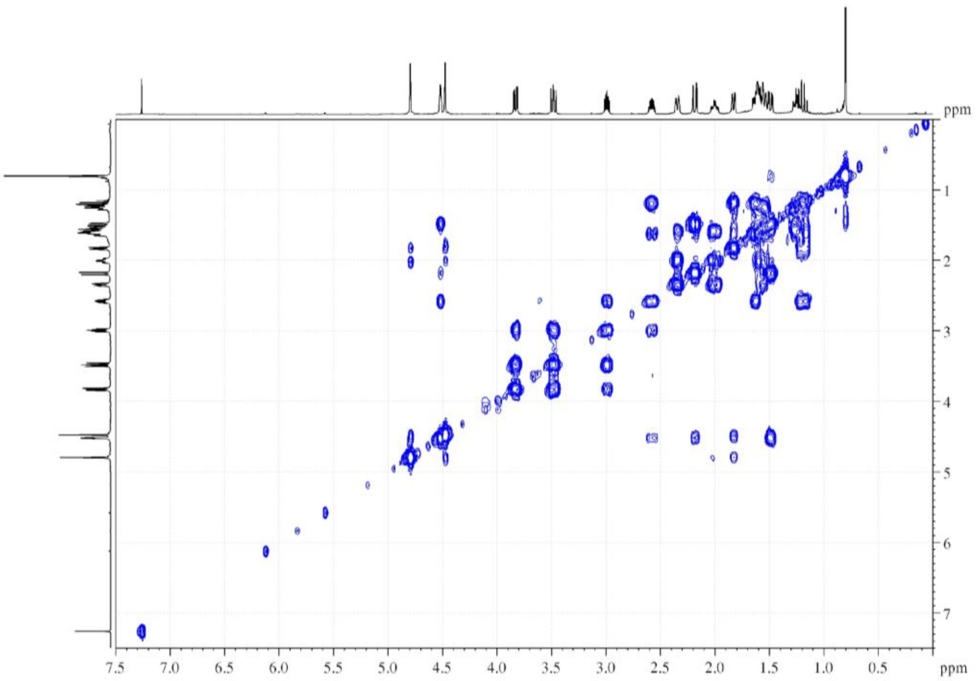

Figure S2.55: COSY spectrum (500 MHz, $CDCl_3$) of diastereoisomer **11a**.

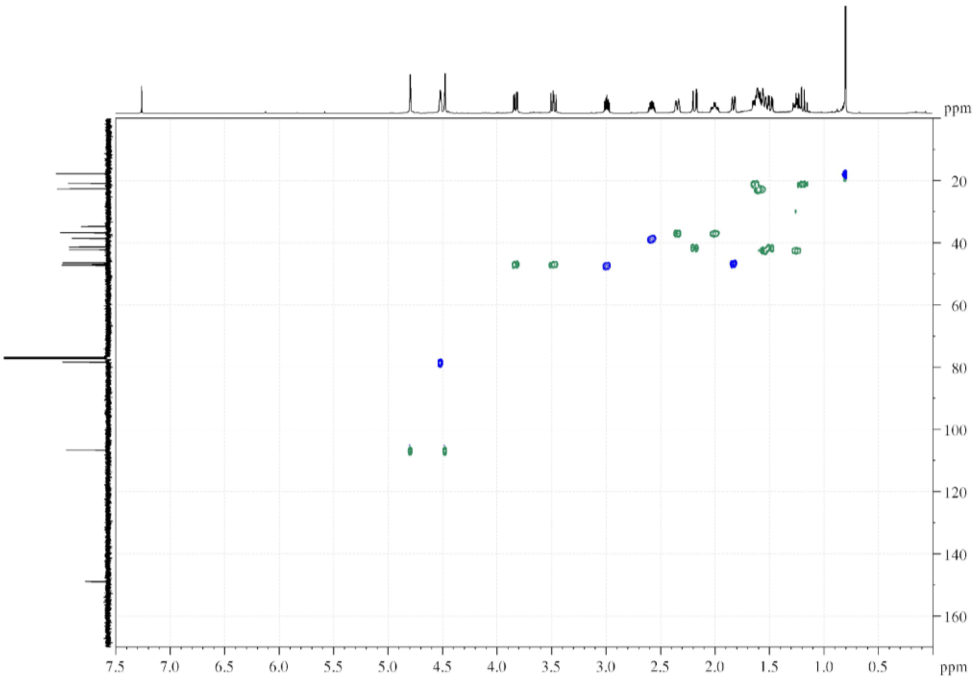

Figure S2.56: HSQC spectrum (500 MHz, $CDCl_3$) of diastereoisomer **11a**.

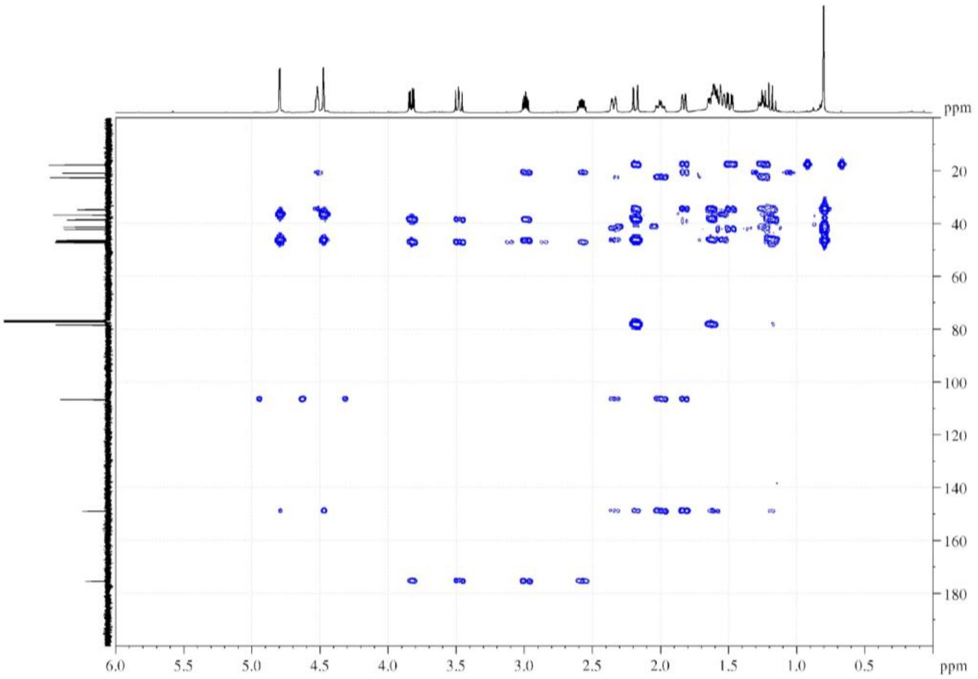

Figure S2.57: HMBC spectrum (500 MHz, $CDCl_3$) of diastereoisomer **11a**.

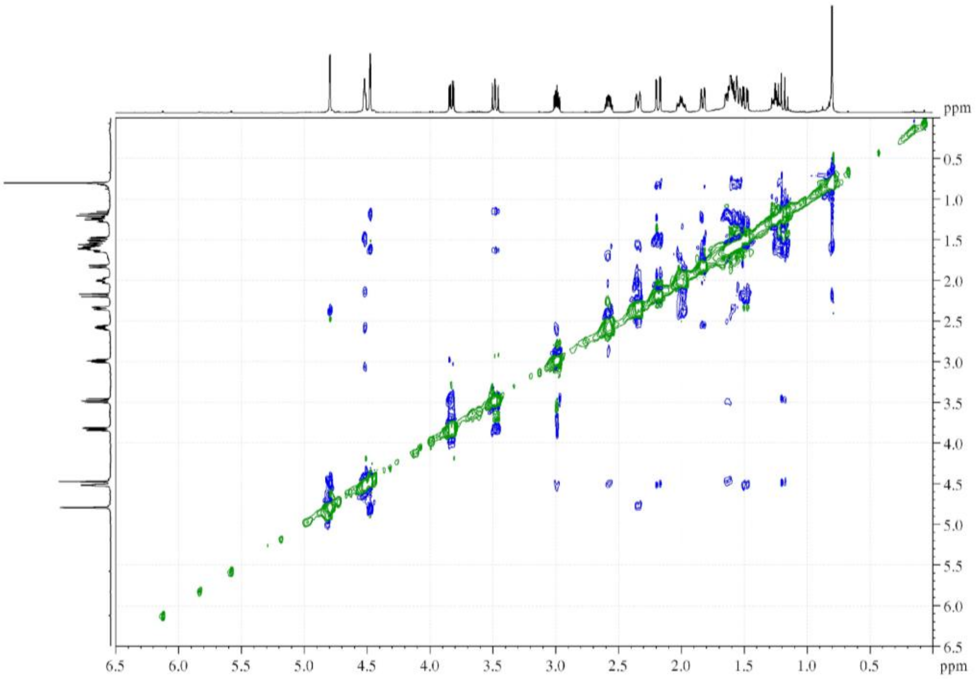

Figure S2.58: ^{1}H-^{1}H NOESY spectrum (500 MHz, $CDCl_3$) of diastereoisomer **11a**.

*Synthesis of (3R)-3-(aminomethyl)-8a-methyl-5-methylenedecahydronaphtho[2,3-b]furan-2(3H)-one **(12)***

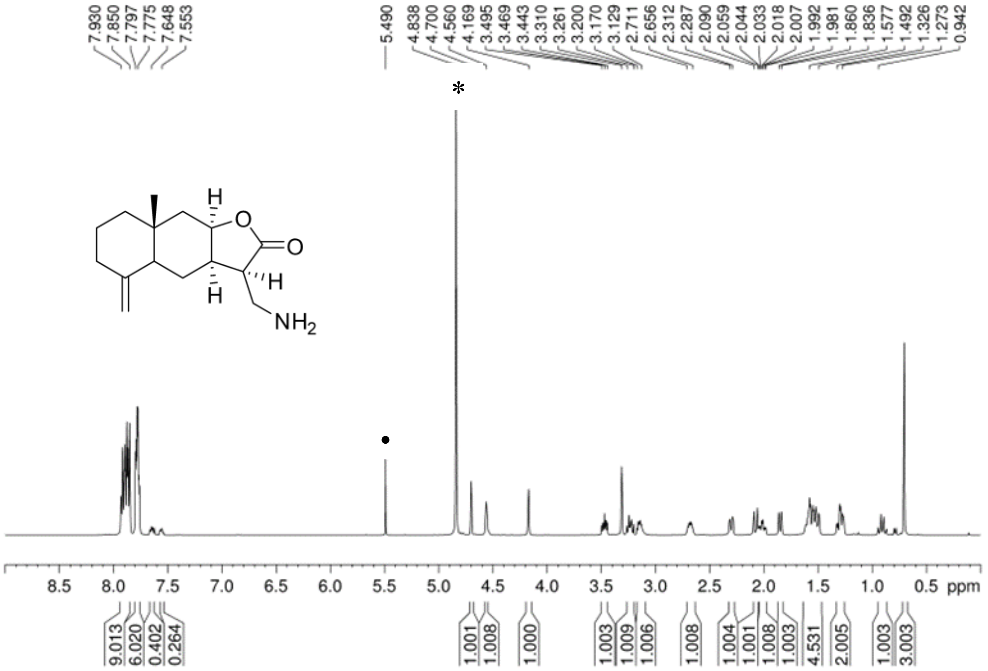

*H_2O, •CH_2Cl_2

Figure S2.59: ^{1}H-NMR spectrum (500 MHz, CD_3OD) of diastereoisomer **12a**.

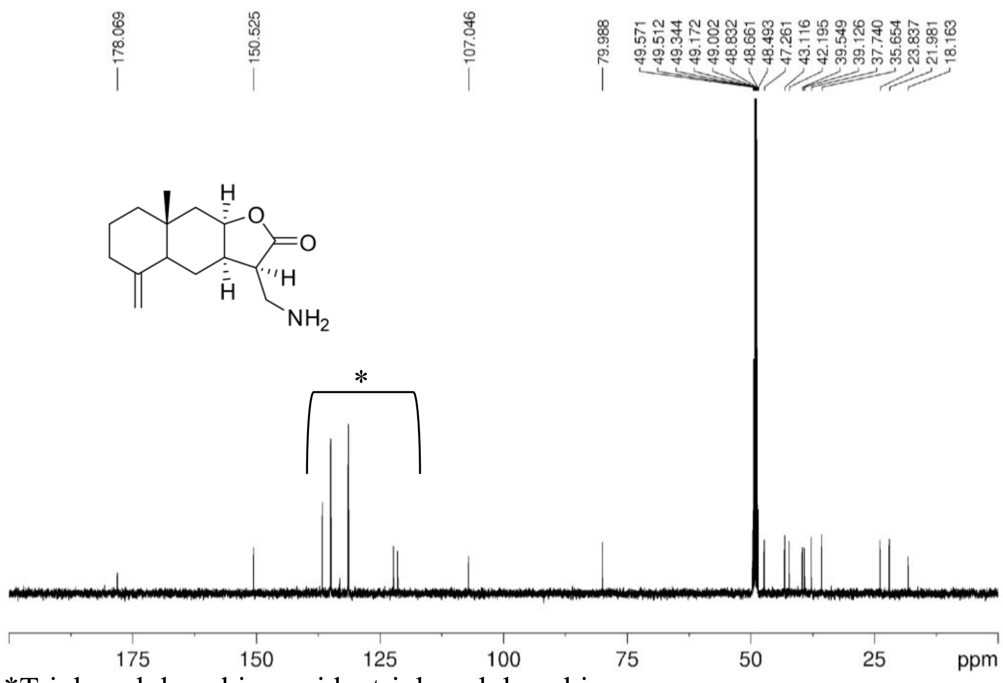

*Triphenylphosphine oxide, triphenylphosphine

Figure S2.60: ^{13}C-NMR spectrum (500 MHz, CD_3OD) of diastereoisomer **12a**.

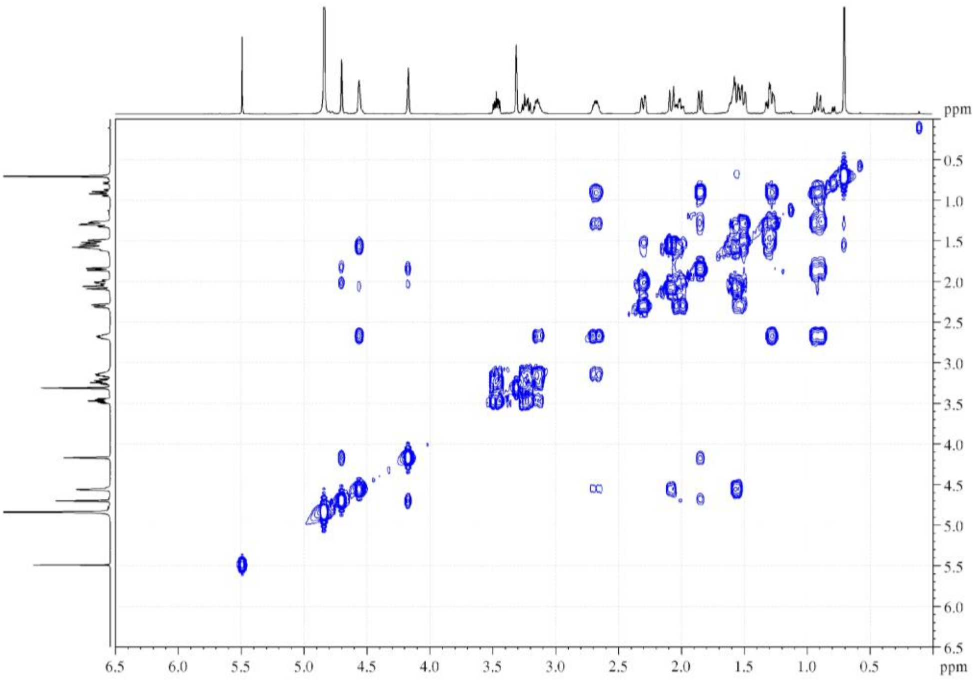

Figure S2.61: COSY spectrum (500 MHz, CD_3OD) of diastereoisomer **12a**.

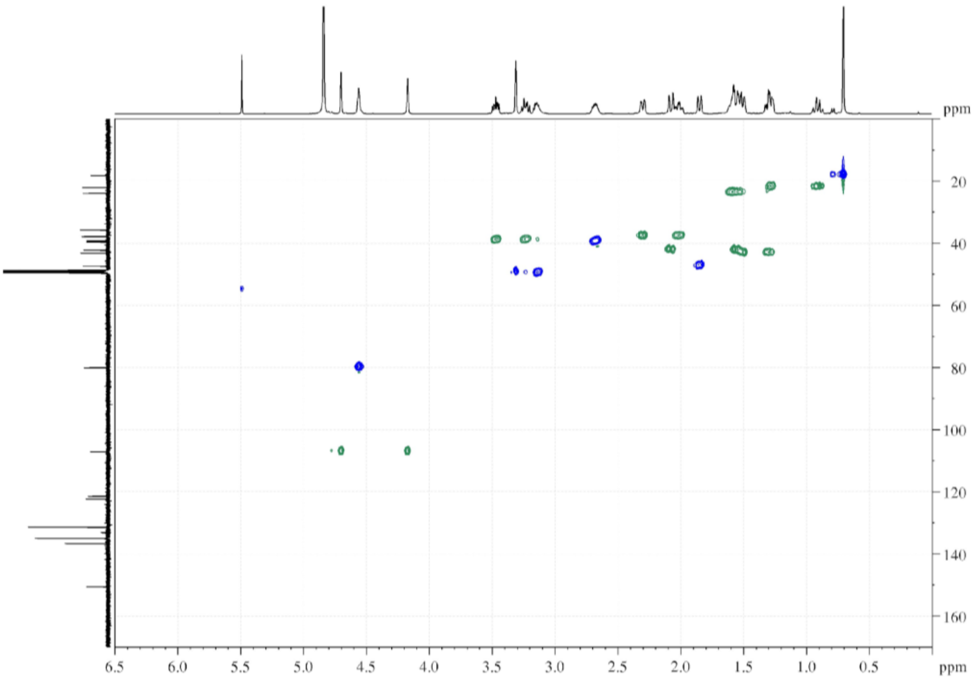

Figure S2.62: HSQC spectrum (500 MHz, CD_3OD) of diastereoisomer **12a**.

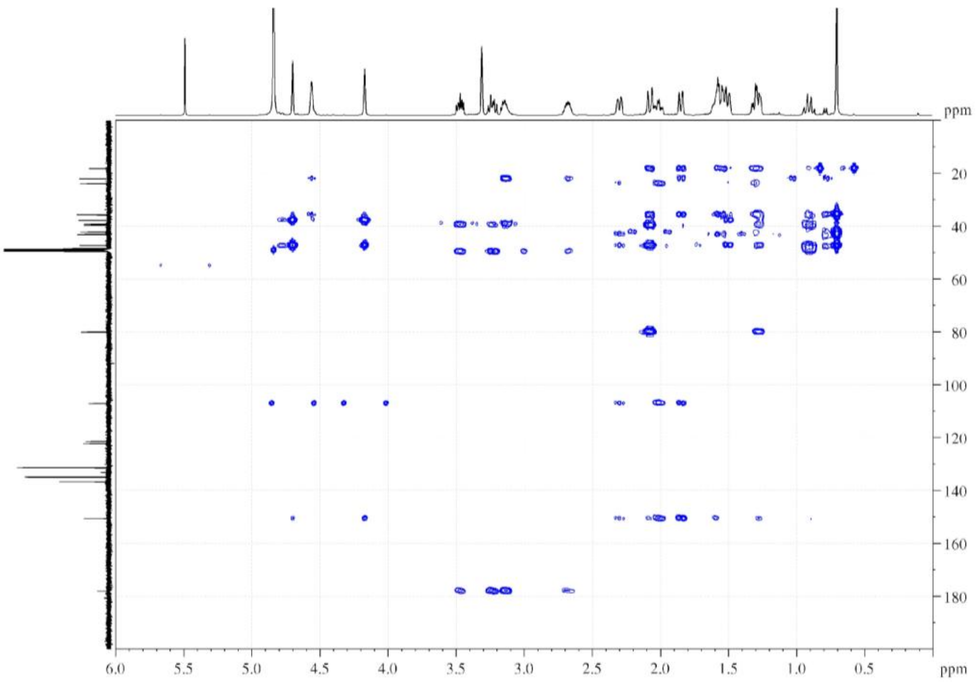

Figure S2.63: HMBC spectrum (500 MHz, CD_3OD) of diastereoisomer **12a**.

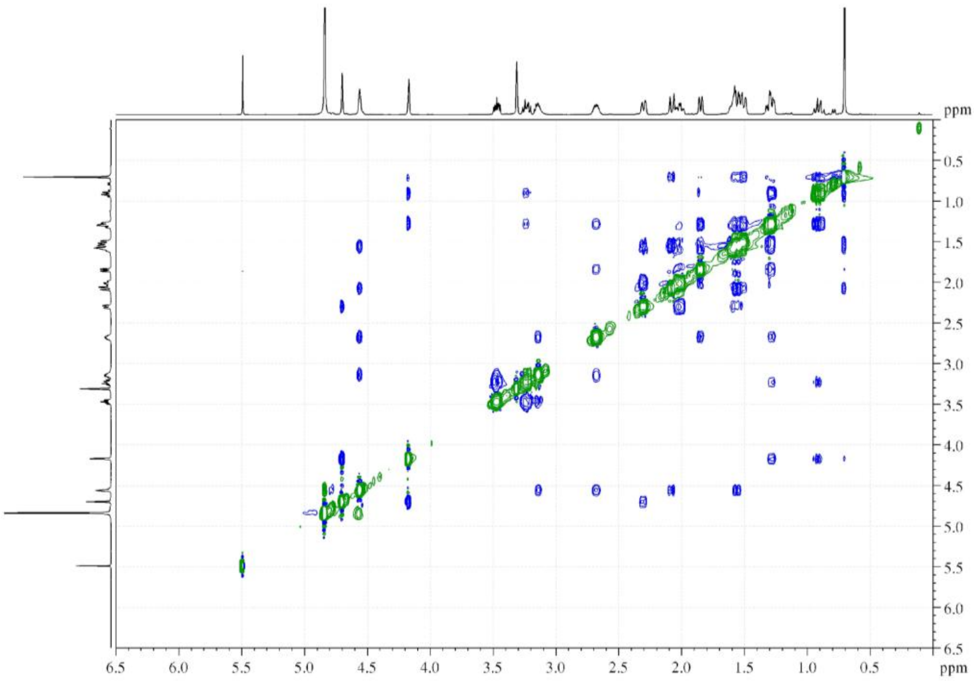

Figure S2.64: ^{1}H-^{1}H NOESY spectrum (500 MHz, CD_3OD) of diastereoisomer **12a**.

*11,13-dihydro-alantolactone (**3a**)*

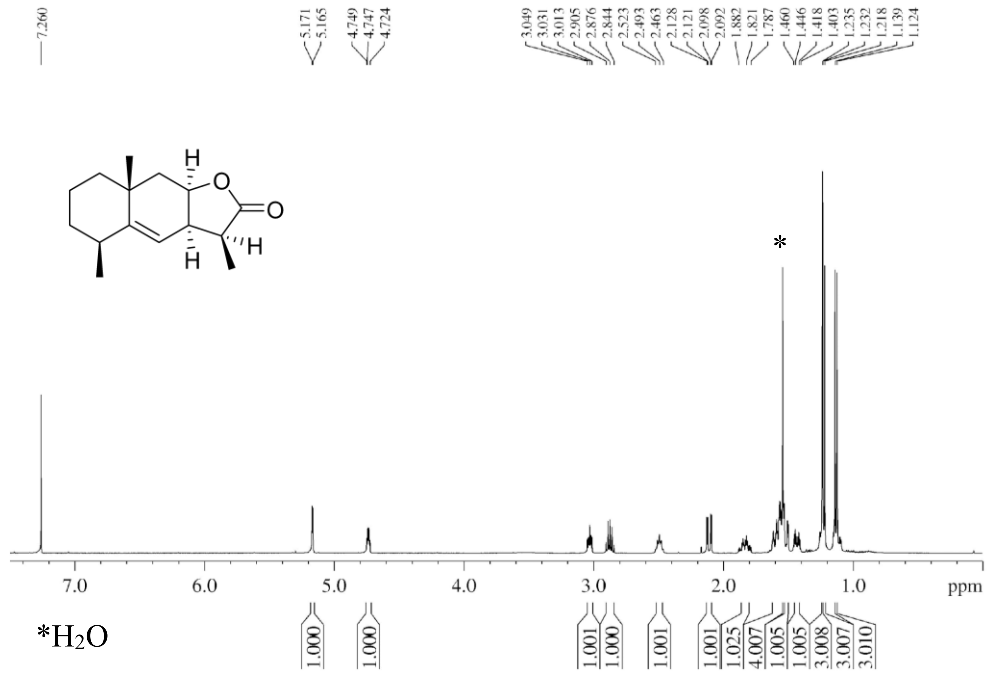

Figure S2.65: ^{1}H-NMR spectrum (500 MHz, $CDCl_3$) of 11,13-dihydro-alantolactone (**3a**).

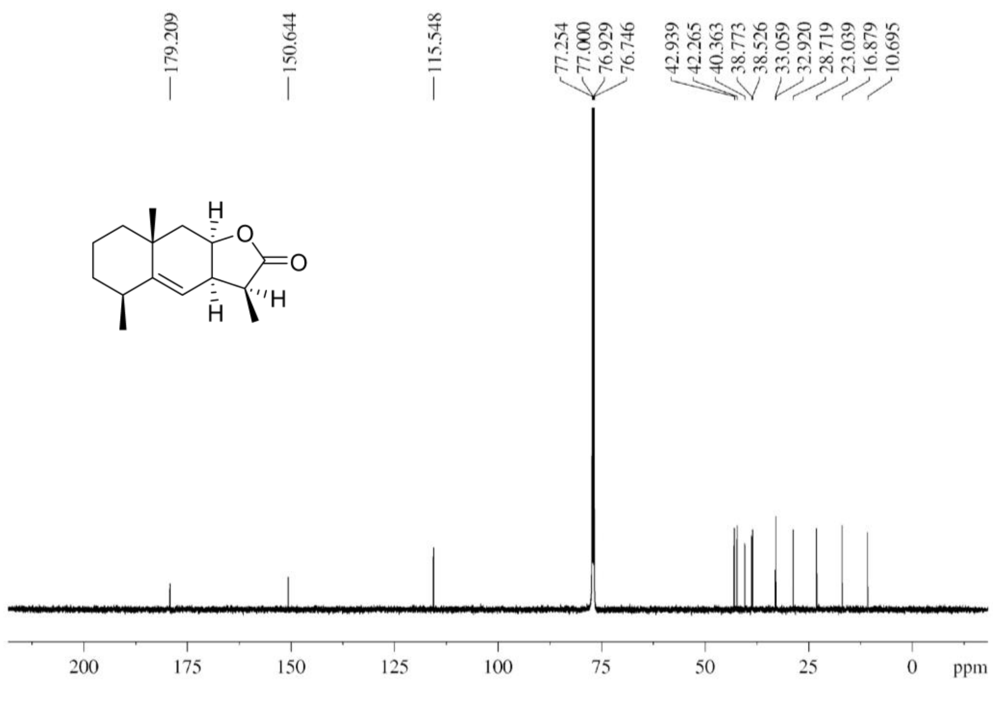

Figure S2.66: ^{13}C-NMR spectrum (500 MHz, $CDCl_3$) of 11,13-dihydro-alantolactone (**3a**).

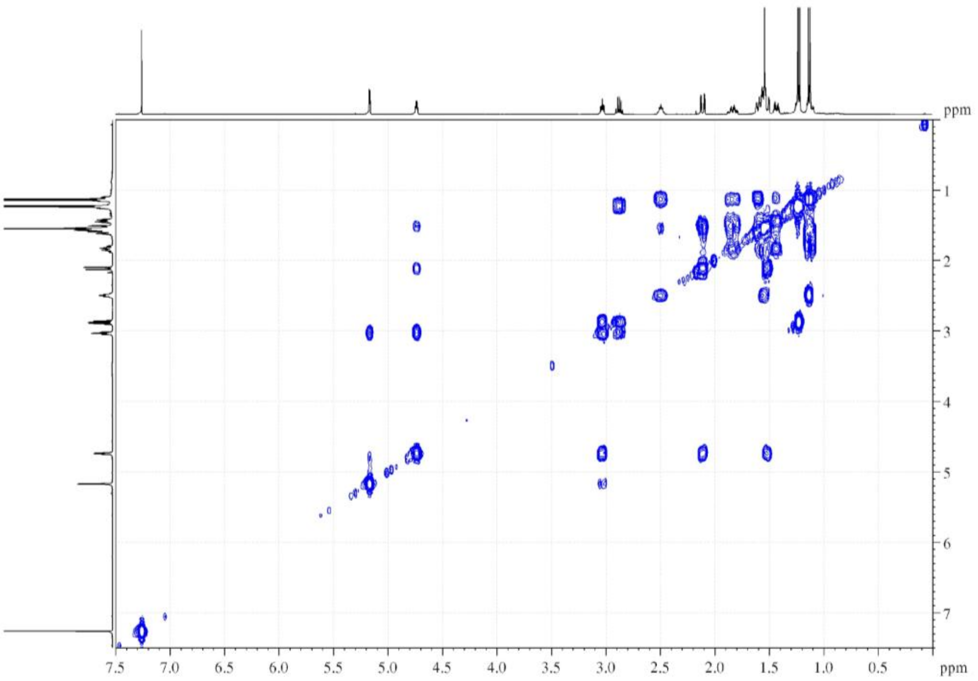

Figure S2.67: COSY spectrum (500 MHz, $CDCl_3$) of 11,13-dihydro-alantolactone (**3a**).

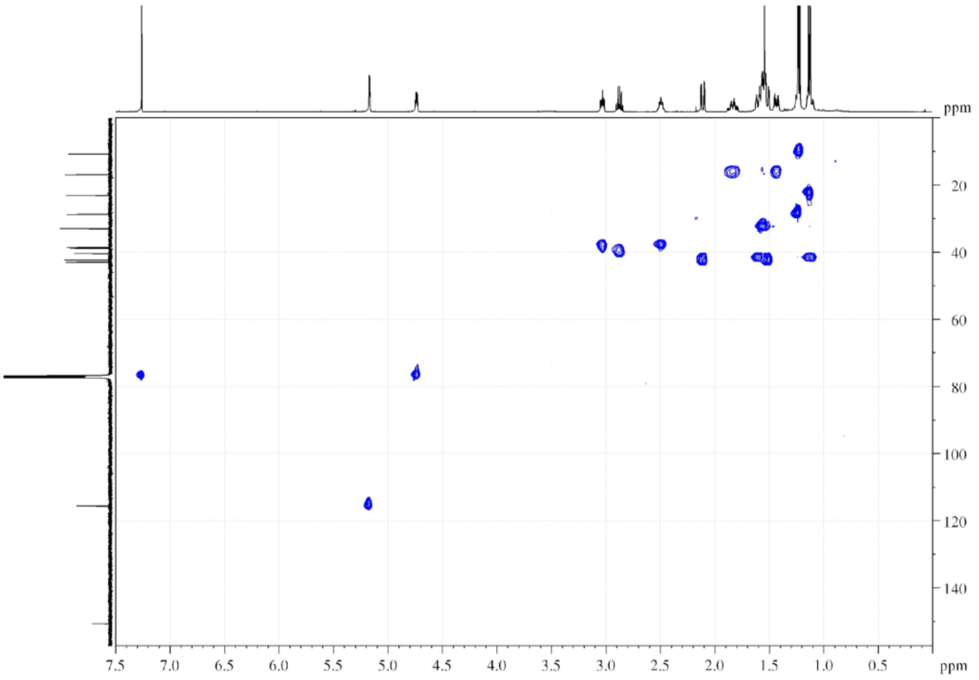

Figure S2.68: HSQC spectrum (500 MHz, $CDCl_3$) of 11,13-dihydro-alantolactone (**3a**).

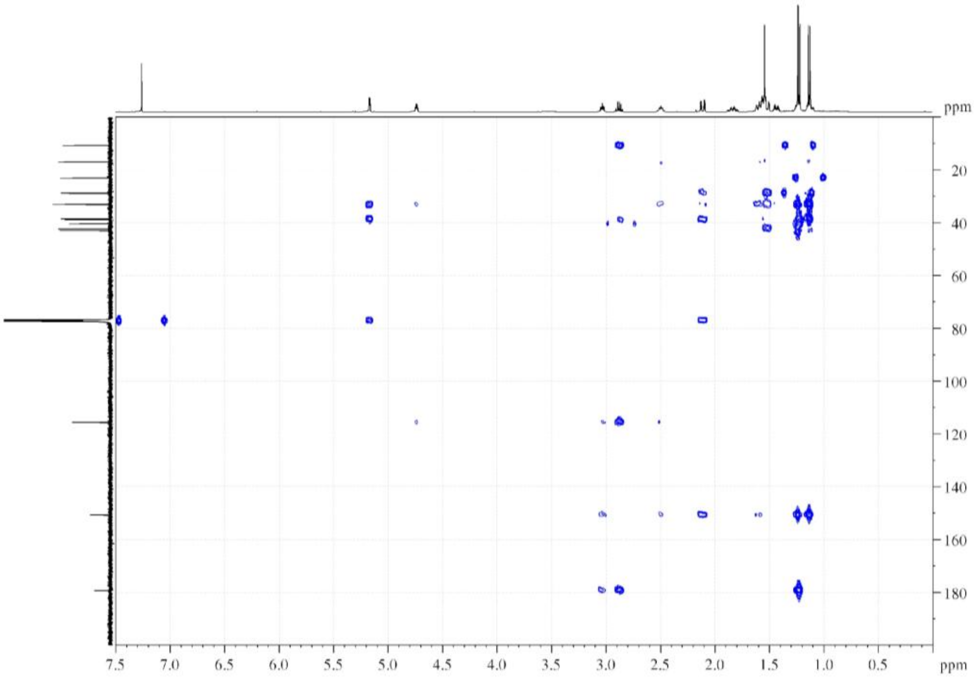

Figure S2.69: HMBC spectrum (500 MHz, $CDCl_3$) of 11,13-dihydro-alantolactone (**3a**).

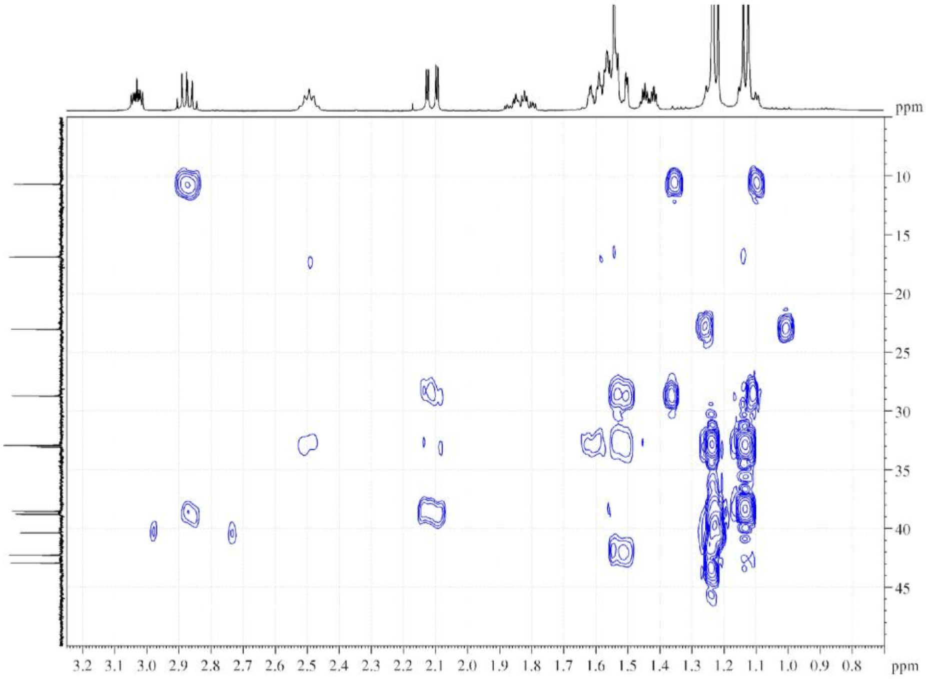

Figure S.270: HMBC spectrum (500 MHz, $CDCl_3$) of 11,13-dihydro-alantolactone (**3a**) with focus on the low ppm region.

*11,13-dihydro-isoalantolactone (**4a**)*

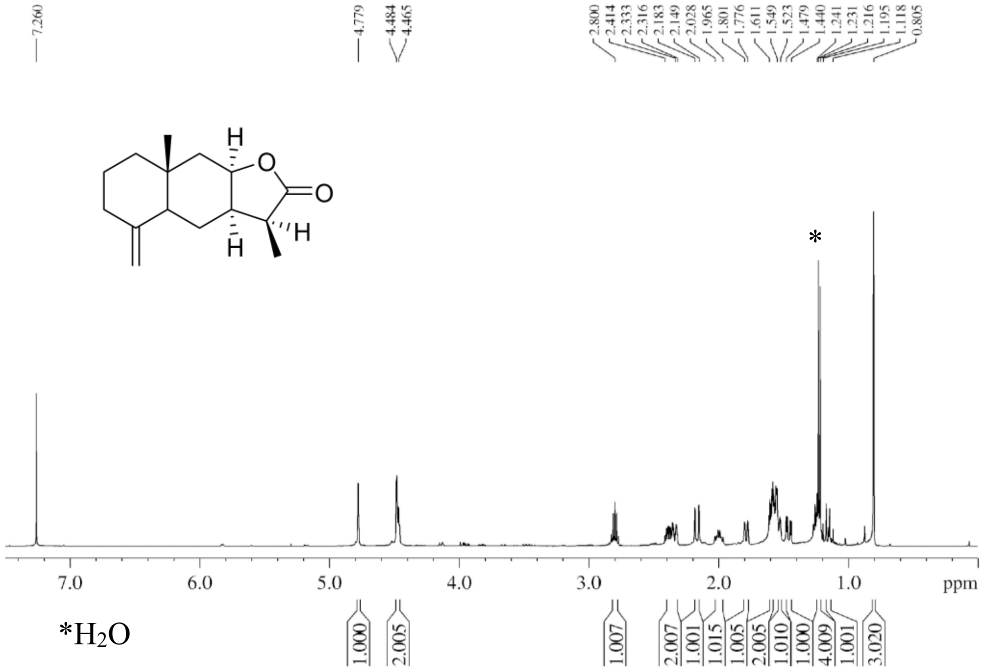

Figure S2.71 : ^{1}H-NMR spectrum (500 MHz, $CDCl_3$) of 11,13-dihydro-isoalantolactone (**4a**).

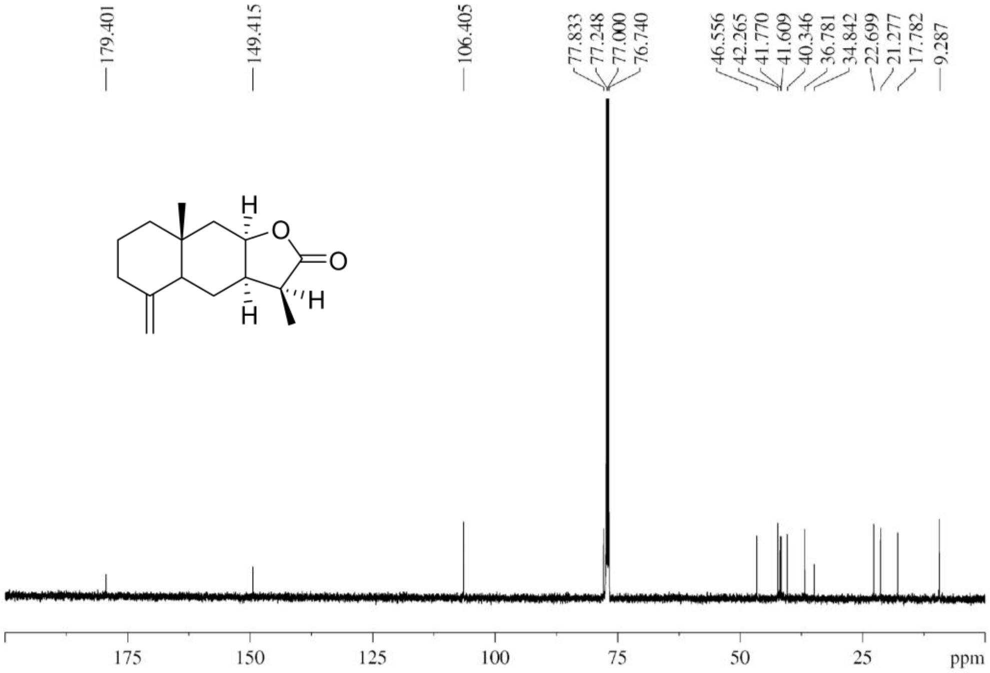

Figure S2.72: ^{13}C-NMR spectrum (500 MHz, $CDCl_3$) of 11,13-dihydro-isoalantolactone (**4a**).

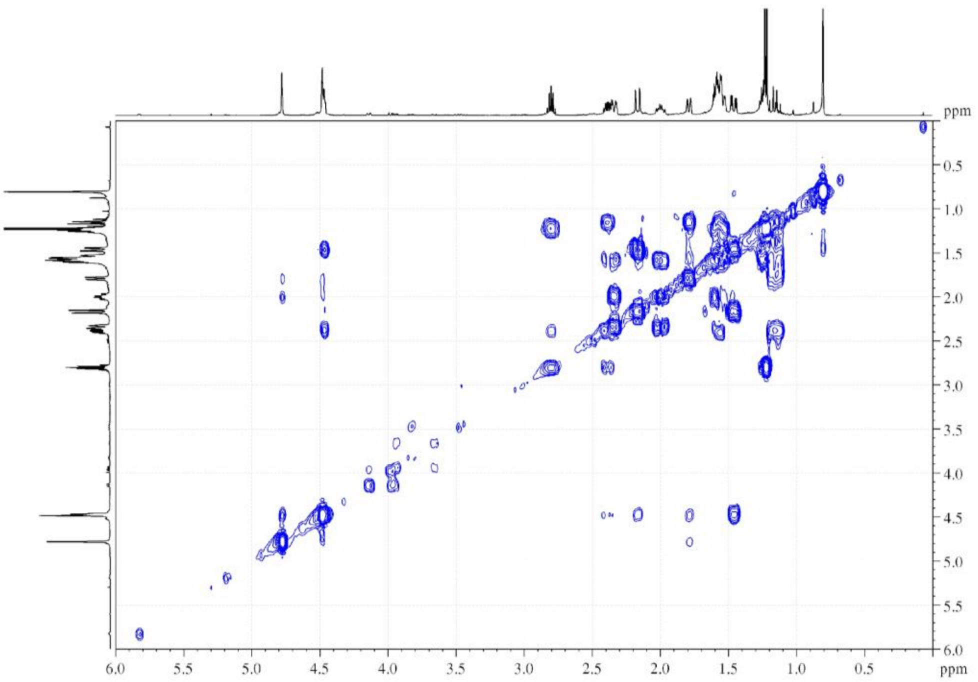

Figure S2.73: COSY spectrum (500 MHz, $CDCl_3$) of 11,13-dihydro-isoalantolactone (**4a**).

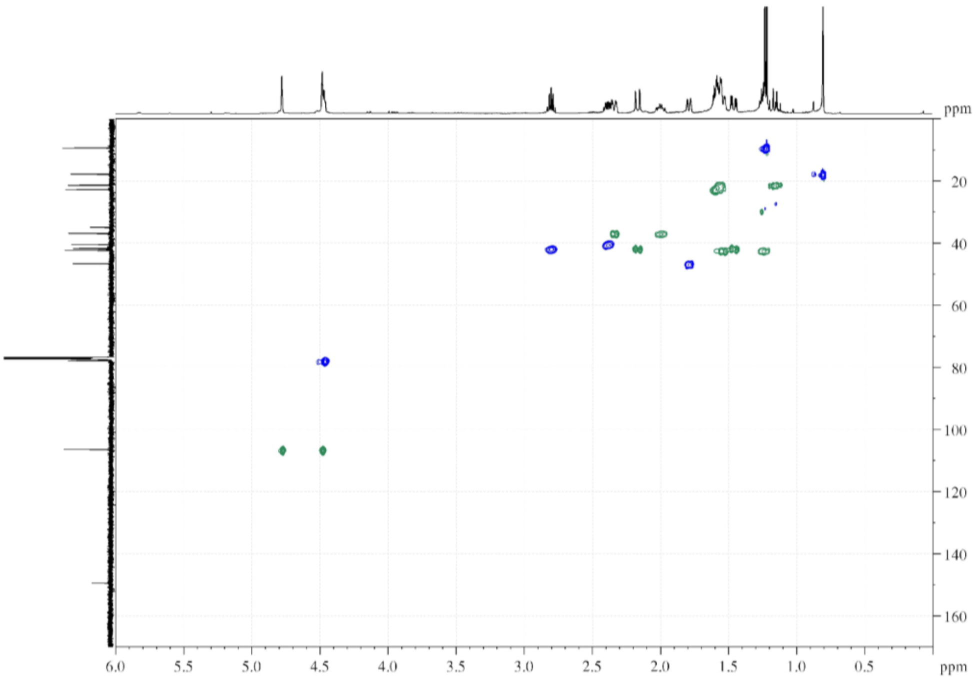

Figure S2.74: HSQC spectrum (500 MHz, $CDCl_3$) of 11,13-dihydro-isoalantolactone (**4a**).

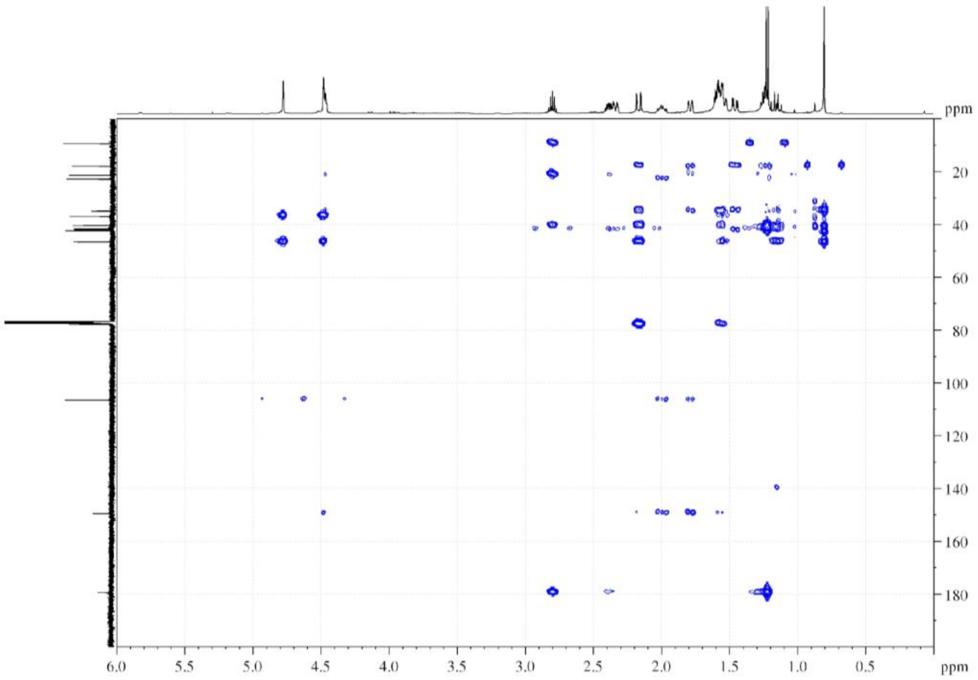

Figure S2.75: HMBC spectrum (500 MHz, $CDCl_3$) of 11,13-dihydro-isoalantolactone (**4a**).

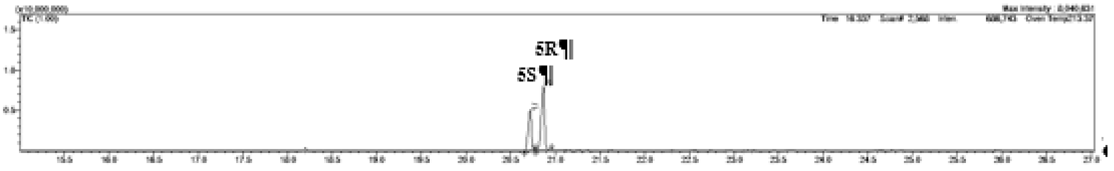

Figure S2.76: GC-MS chromatogram for diastereoisomer mixture of alantolactone nitriles **5R, 5S**

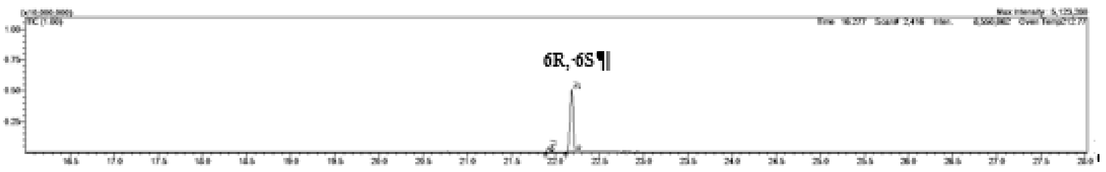

Figure S2.77: GC-MS chromatogram for diastereoisomer mixture of TMS derivatives of alantolactone acids **6R, 6S**.

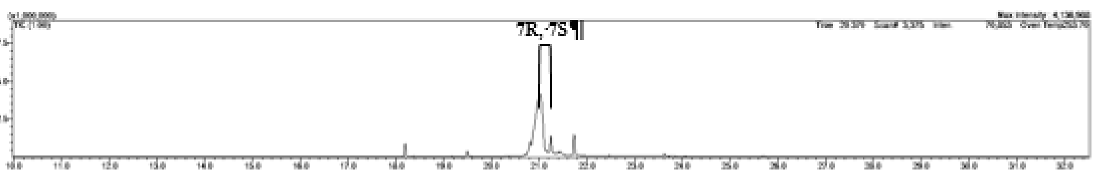

Figure S2.78: GC-MS chromatogram for diastereoisomer mixture of alantolactone azides **7R, 7S**.

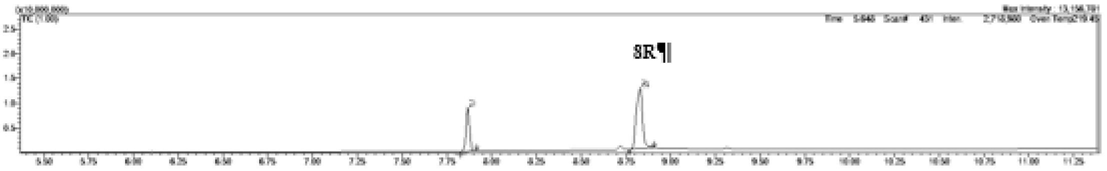

Figure S2.79: GC-MS chromatogram of alantolactone amine **8R**

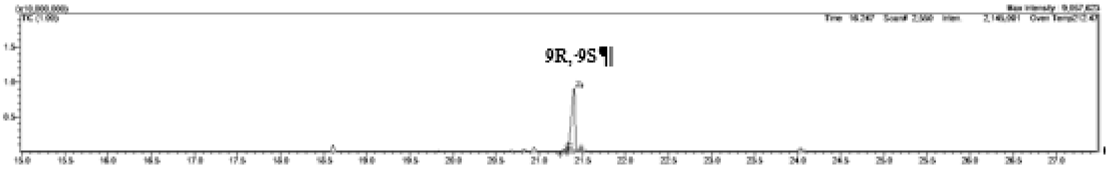

Figure S2.80: GC-MS chromatogram for diastereoisomer mixture of isoalantolactone nitriles **9R, 9S.**

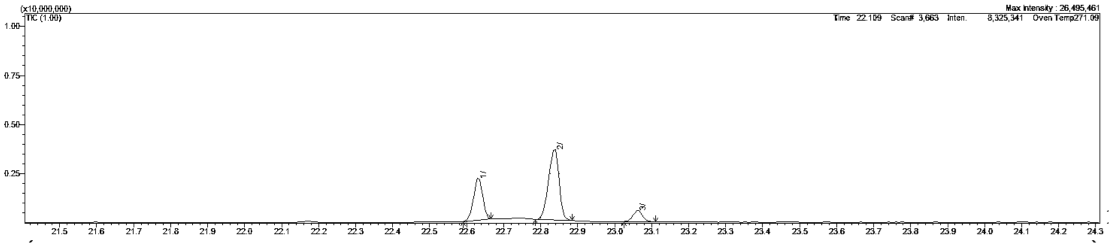

Figure S2.81: GC-MS chromatogram for diastereoisomer mixture of TMS derivatives of isoalantolactone acids **10R, 10S.**

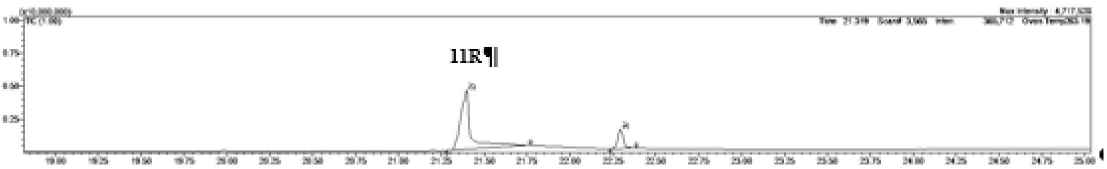

Figure S2.82: GC-MS chromatogram of isoalantolactone azide **11R**

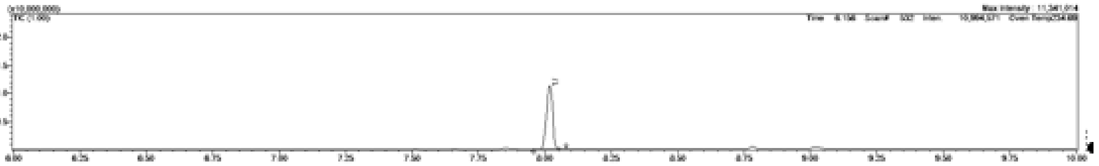

Figure S2.83: GC-MS chromatogram of isoalantolactone amine **12R**

3. Biological assays

A. Acaricidal Studies

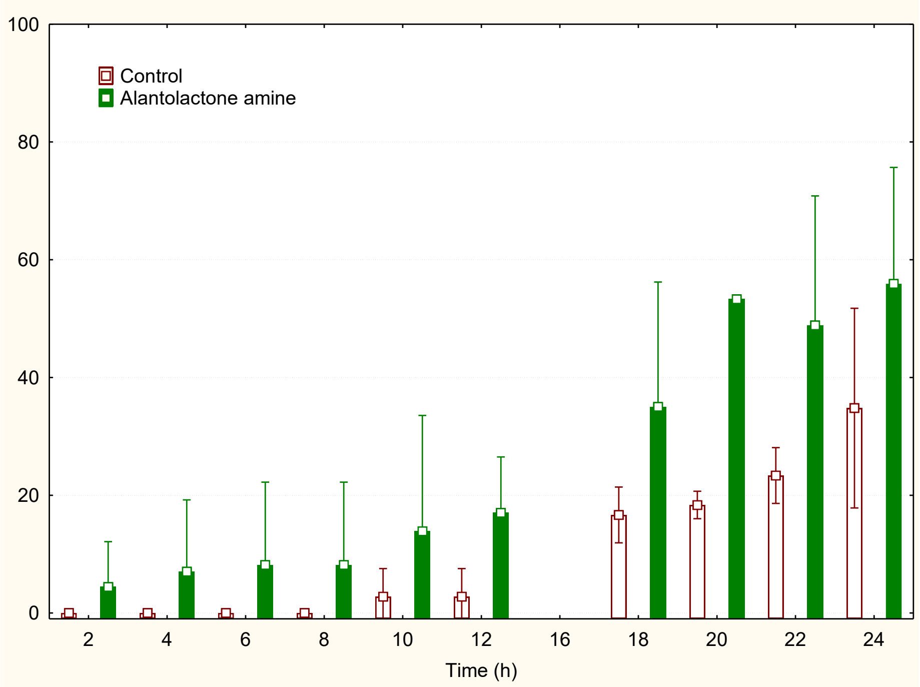

Figure S3.1: Percentage of varroa mortality upon exposure to compound **8.**

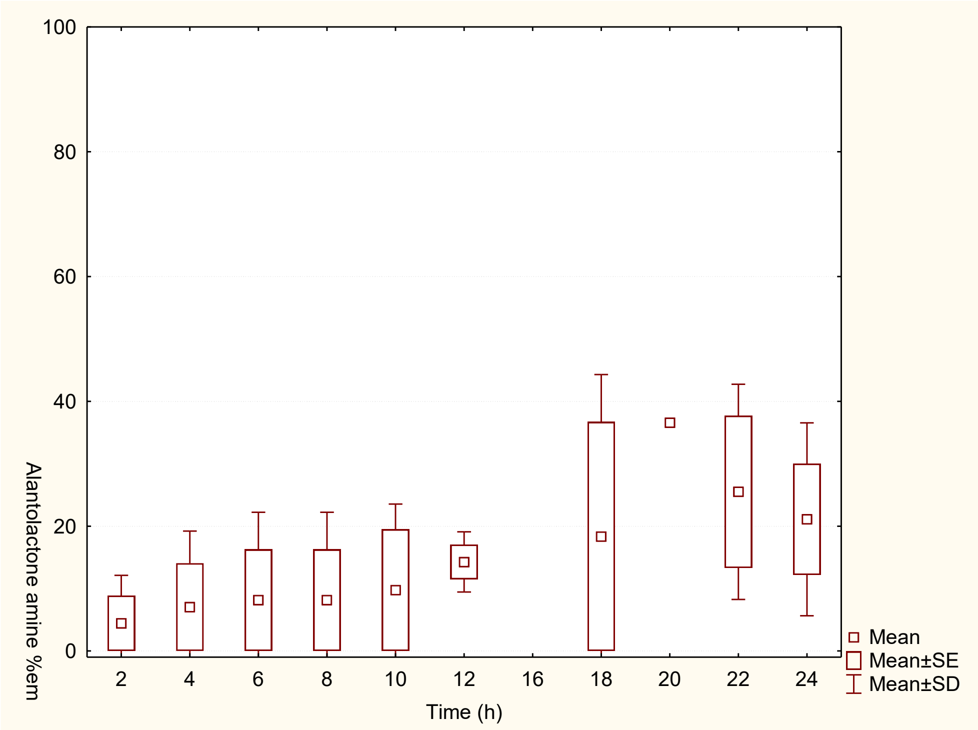

Figure S3.2: Percentage of mortality when control is subtracted from measurements.

Table S3.1: Average p values in the t-tests on pairs of natural and/or synthetic compounds

Pair of acaricides	Average p value
t-test alantolactone-isoalantolactone	0.23
t-test alantolactone acid - isoalantolactone acid	0.20
t-test alantolactone - alantolactone acid	0.31
t-test isoalantolactone - isoalantolactone acid	0.23

Table S3.2: Results on t-test of alantolactone activity vs control values

Time (hr)	P values
2	-
4	0.19
6	0.19
8	0.06
10	-
12	0.03
16	-
18	0.03
20	0.03
22	0.02
24	0.03

Table S.3.3: Results on t-test of isoalantolactone activity vs control values

Time (hr)	P values
2	0.19
4	0.11
6	0.06
8	0.07
10	-
12	0.04
16	-
18	0.03
20	0.08
22	0.07
24	0.07

Table S.3.4: Results on t-test of alantolactone acid activity vs control values

Time (hr)	P values
2	-
4	-

6	0.18
8	0.07
10	0.16
12	0.02
16	-
18	0.02
20	0.01
22	0.02
24	0.04

Table S.3.5: Results on t-test of isoalantolactone acid activity vs control values

Time (hr)	P values
2	-
4	0.18
6	0.13
8	0.09
10	0.34
12	0.25
16	-
18	0.02
20	0.01
22	0.01
24	0.02

B. Antioxidant Studies

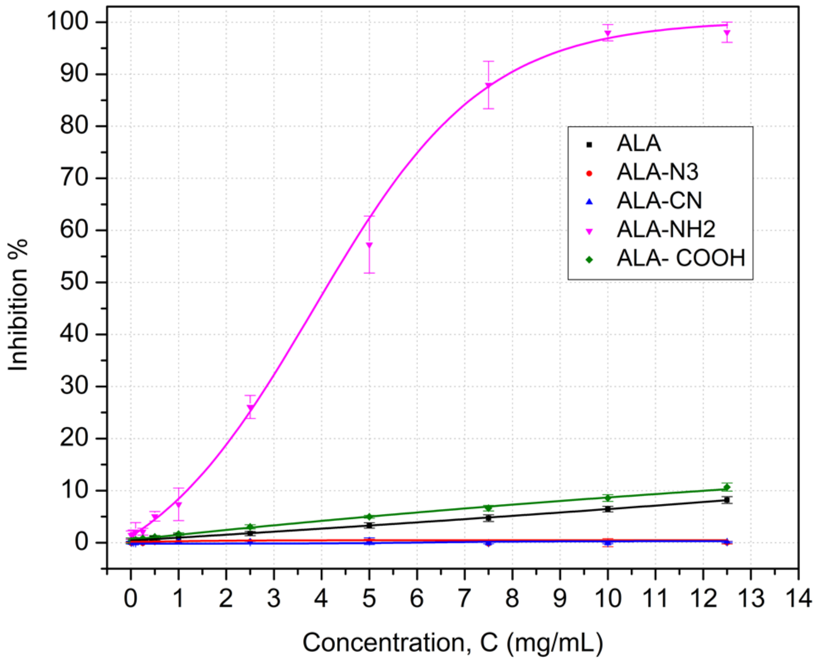

Figure S3.3: Antioxidant activity of alantolactone derivatives, measuring percentage of DPPH radical inhibition.

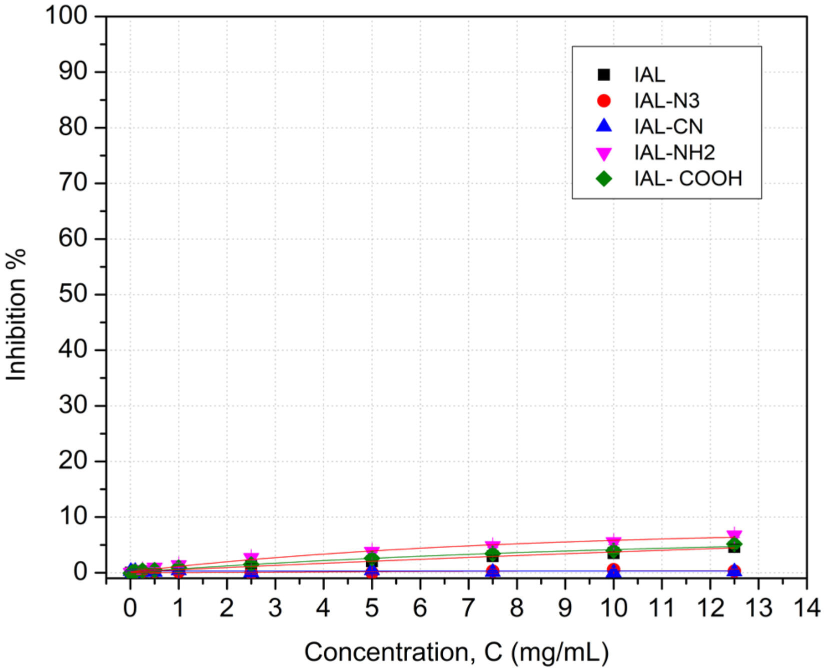

Figure S3.4: Antioxidant activity of alantolactone derivatives, measuring percentage of DPPH radical inhibition.

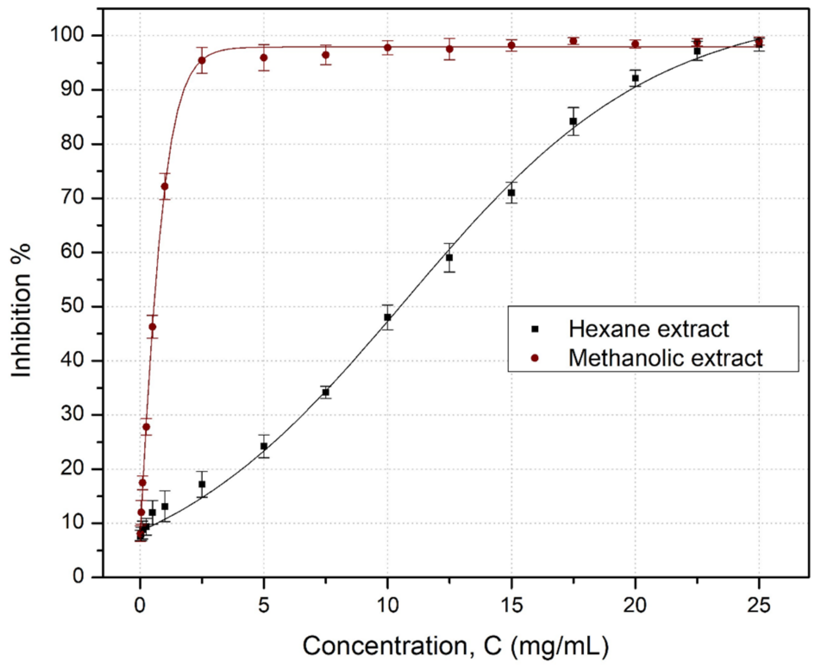

Figure S3.5: Antioxidant activity of *I. Helenium* extracts, measuring percentage of DPPH radical inhibition.
